# Supplementary material for: Neobacillus driksii sp. nov. isolated from a Mars 2020 spacecraft assembly facility and genomic potential for lasso peptide production in Neobacillus
Source: Microbiol Spectr. 2024 Nov 29;13(1):e01376-24. doi: 10.1128/spectrum.01376-24 (PMC11705953; doi:10.1128/spectrum.01376-24)
Supplement: Supplemental material — Supplemental figures and tables. [file spectrum.01376-24-s0001.pdf]

Supplemental File S1. ANI matrix of all genomes of *Neobacillus* strains.

GenBank Accession #	Scientific Name	*Neobacillus driksii 179-C4-2-HS(T)*	*Neobacillus driksii 179-J1A1-HS*	*Neobacillus driksii AT2.8*	*Neobacillus driksii V4I25*	*Neobacillus niacini NBRC 15566*	*Neobacillus terrae*	*Neobacillus mesonae*	*Neobacillus niacini*	*Neobacillus sp. PS3-34*	*Neobacillus sp. SuZ13*	*Neobacillus cucumis*	*Neobacillus vireti*	*Neobacillus sp. OS1-33*	*Neobacillus cucumis*	*Neobacillus drentensis*	*Neobacillus drentensis*	*Neobacillus vireti*	*Neobacillus cucumis*	*Neobacillus thermocopriae*	*Neobacillus bataviensis LMG 21833*	*Neobacillus vireti*	*Neobacillus drentensis*	*Neobacillus cucumis*	*Neobacillus piezotolerans*	*Neobacillus endophyticus*	*Neobacillus sp. YX16*	*Neobacillus drentensis*	*Neobacillus sp. DY30*	*Neobacillus drentensis*	*Neobacillus sp. YIM B06451*	*Neobacillus paridis*
**GCA_030732025.1**	***Neobacillus driksii 179-C4-2-HS(T)***	**100.0**																														
**JBDZYE000000000**	***Neobacillus driksii 179-J1A1-HS***	**97.1**	**100.0**																													
**GCA_013409995.1**	***Neobacillus driksii AT2.8***	**96.0**	**96.1**	**100.0**																												
**GCA_030817595.1**	***Neobacillus driksii V4I25***	**95.1**	**95.1**	**95.1**	**100.0**																											
**GCA_001591505.1**	***Neobacillus niacini NBRC 15566***	**86.4**	**86.4**	**86.8**	**86.6**	**100.0**																										
GCA_011393025.1	*Neobacillus terrae*	**< 77**	**< 77**	**< 77**	**< 77**	**< 77**	100.0																									
GCA_023715865.1	*Neobacillus mesonae*	**< 77**	**< 77**	**< 77**	**< 77**	**< 77**	< 77	100.0																								
GCA_035809615.1	*Neobacillus niacini*	**86.5**	**86.4**	**86.9**	**86.6**	**100.0**	< 77	< 77	100.0																							
GCA_030915465.1	*Neobacillus sp. PS3-34*	**< 77**	**< 77**	**< 77**	**< 77**	**< 77**	78.8	< 77	< 77	100.0																						
GCA_030123365.1	*Neobacillus sp. SuZ13*	**79.5**	**78.8**	**79.6**	**79.6**	**78.8**	< 77	< 77	79.0	< 77	100.0																					
GCA_036223585.1	*Neobacillus cucumis*	**78.9**	**78.6**	**78.8**	**78.8**	**< 77**	< 77	< 77	< 77	< 77	79.8	100.0																				
GCA_036962345.1	*Neobacillus vireti*	**82.5**	**82.3**	**82.6**	**82.4**	**82.7**	< 77	< 77	82.9	< 77	78.9	78.6	100.0																			
GCA_030915405.1	*Neobacillus sp. OS1-33*	**79.3**	**78.8**	**78.9**	**79.5**	**78.4**	< 77	< 77	78.4	< 77	83.0	78.8	78.5	100.0																		
GCA_023715505.1	*Neobacillus cucumis*	**78.7**	**78.8**	**78.7**	**78.6**	**78.3**	< 77	< 77	78.4	< 77	79.4	83.1	78.7	79.2	100.0																	
GCA_036962725.1	*Neobacillus drentensis*	**78.5**	**78.4**	**78.6**	**78.5**	**78.4**	< 77	< 77	78.5	< 77	79.5	79.0	78.5	79.4	79.3	100.0																
GCA_036962365.1	*Neobacillus drentensis*	**78.9**	**78.8**	**78.9**	**78.9**	**78.5**	< 77	< 77	78.5	< 77	82.9	78.9	78.4	97.2	79.3	79.2	100.0															
GCA_036965785.1	*Neobacillus vireti*	**78.7**	**78.7**	**78.9**	**78.9**	**78.2**	< 77	< 77	78.3	< 77	79.9	83.5	78.6	79.2	93.6	79.2	79.4	100.0														
GCA_002860255.1	*Neobacillus cucumis*	**78.6**	**78.1**	**78.5**	**78.3**	**78.4**	< 77	< 77	78.5	< 77	79.8	82.7	78.5	78.9	83.7	79.4	79.0	84.2	100.0													
GCA_036219545.1	*Neobacillus thermocopriae*	**77.8**	**77.6**	**77.6**	**77.6**	**77.6**	< 77	< 77	77.9	< 77	77.8	77.9	77.8	77.7	77.7	78.1	77.8	77.9	78.4	100.0												
GCA_000307875.1	*Neobacillus bataviensis LMG 21833*	**78.3**	**78.3**	**78.3**	**78.2**	**78.5**	< 77	< 77	78.6	< 77	79.0	79.1	78.6	79.3	79.4	79.3	79.2	79.4	79.3	77.9	100.0											
GCA_036961715.1	*Neobacillus vireti*	**82.5**	**82.5**	**82.6**	**82.6**	**82.9**	< 77	< 77	83.0	< 77	78.8	78.4	98.7	78.2	78.6	78.4	78.3	78.6	78.5	< 77	78.5	100.0										
GCA_036962765.1	*Neobacillus drentensis*	**78.9**	**78.5**	**78.9**	**79.0**	**78.6**	< 77	< 77	78.6	< 77	85.5	79.0	78.6	82.7	79.2	79.7	82.7	79.5	79.3	77.6	79.2	78.5	100.0									
GCA_031324245.1	*Neobacillus cucumis*	**79.0**	**78.4**	**78.8**	**79.2**	**< 77**	< 77	< 77	< 77	< 77	80.5	95.4	78.7	79.4	83.0	79.0	79.1	83.5	82.9	77.7	79.2	78.7	79.1	100.0								
GCA_003362805.1	*Neobacillus piezotolerans*	**< 77**	**< 77**	**< 77**	**< 77**	**< 77**	< 77	< 77	< 77	< 77	< 77	< 77	< 77	< 77	< 77	< 77	< 77	< 77	< 77	< 77	< 77	< 77	< 77	< 77	100.0							
GCA_013248975.1	*Neobacillus endophyticus*	**< 77**	**< 77**	**< 77**	**< 77**	**< 77**	< 77	< 77	< 77	< 77	79.2	78.5	< 77	78.9	78.2	78.2	78.0	78.5	78.5	78.0	78.1	< 77	< 77	79.1	< 77	100.0						
GCA_030123505.1	*Neobacillus sp. YX16*	**84.5**	**84.2**	**84.5**	**84.6**	**83.4**	< 77	< 77	83.5	< 77	79.7	78.5	82.6	79.7	78.7	78.9	78.7	78.7	78.6	77.6	78.5	82.5	79.5	79.1	< 77	< 77	100.0					
GCA_036962905.1	*Neobacillus drentensis*	**78.9**	**78.7**	**78.7**	**78.8**	**78.5**	< 77	< 77	78.6	< 77	83.1	78.9	78.4	97.2	79.2	79.2	99.1	79.4	79.1	77.5	79.2	78.2	82.8	79.2	< 77	78.1	78.8	100.0				
GCA_030123065.1	*Neobacillus sp. DY30*	**86.5**	**86.1**	**86.5**	**86.6**	**87.0**	< 77	< 77	87.0	< 77	79.8	78.9	82.4	79.4	78.6	78.8	78.7	78.7	78.7	77.7	78.3	82.3	79.0	79.5	< 77	< 77	83.7	78.7	100.0			
GCA_031457825.1	*Neobacillus drentensis*	**78.6**	**78.7**	**78.9**	**78.8**	**78.4**	< 77	< 77	78.4	< 77	82.8	79.0	78.9	93.1	79.2	79.4	93.3	79.4	79.0	77.6	79.5	78.9	83.2	78.9	< 77	< 77	79.4	93.3	78.8	100.0		
GCA_032667185.1	*Neobacillus sp. YIM B06451*	**< 77**	**< 77**	**< 77**	**< 77**	**< 77**	< 77	< 77	< 77	< 77	< 77	< 77	< 77	< 77	< 77	< 77	< 77	< 77	< 77	< 77	< 77	< 77	< 77	< 77	85.0	< 77	< 77	< 77	< 77	< 77	100.0	
GCA_016765675.1	*Neobacillus paridis*	**< 77**	**< 77**	**< 77**	**< 77**	**< 77**	< 77	< 77	< 77	< 77	< 77	< 77	< 77	77.7	< 77	< 77	< 77	< 77	< 77	77.8	77.7	< 77	< 77	< 77	< 77	< 77	< 77	< 77	< 77	< 77	< 77	100.0
GCA_036962645.1	*Neobacillus niacini*	**82.0**	**82.0**	**81.9**	**82.0**	**82.1**	< 77	< 77	82.1	< 77	78.6	< 77	83.1	78.4	78.2	78.3	78.4	78.1	< 77	77.5	78.4	83.2	78.2	< 77	< 77	< 77	82.6	78.4	81.6	78.6	< 77	< 77
GCA_023715785.1	*Neobacillus niacini*	**< 77**	**< 77**	**< 77**	**< 77**	**< 77**	< 77	< 77	< 77	< 77	< 77	< 77	< 77	< 77	< 77	78.2	77.8	< 77	< 77	77.9	78.5	< 77	< 77	< 77	< 77	< 77	< 77	77.9	< 77	< 77	< 77	78.0
GCA_000508325.2	*Neobacillus vireti LMG 21834*	**78.2**	**78.2**	**78.2**	**78.1**	**78.2**	< 77	< 77	78.3	< 77	79.2	78.7	78.3	79.4	78.8	79.4	79.3	79.0	79.0	77.9	80.9	78.4	79.2	78.8	< 77	78.2	78.4	79.5	78.3	79.5	< 77	78.0
GCA_030915525.1	*Neobacillus sp. PS2-9*	**79.0**	**78.6**	**78.8**	**79.5**	**78.6**	< 77	< 77	78.7	< 77	79.9	79.1	78.6	79.8	79.2	95.9	79.3	79.2	79.4	78.3	79.3	78.5	79.6	79.5	< 77	78.9	79.6	79.4	79.4	79.4	< 77	< 77
GCA_016107705.1	*Neobacillus cucumis*	**78.4**	**78.3**	**78.4**	**78.4**	**78.1**	< 77	< 77	78.2	< 77	79.0	78.6	78.3	78.4	78.8	78.5	78.2	78.7	78.8	77.7	78.6	78.3	78.5	78.8	< 77	78.2	78.5	78.4	78.7	78.6	< 77	< 77
GCA_036961885.1	*Neobacillus niacini*	**82.0**	**82.0**	**81.9**	**81.9**	**82.1**	< 77	< 77	82.1	< 77	78.4	< 77	83.0	78.5	78.2	78.6	78.4	78.1	78.2	77.5	78.5	83.1	78.2	78.1	< 77	< 77	82.6	78.4	81.6	78.7	< 77	< 77
GCA_001591485.1	*Neobacillus fumarioli NBRC 102428*	**77.3**	**< 77**	**77.2**	**77.3**	**77.4**	< 77	< 77	77.6	< 77	77.5	77.9	< 77	77.6	77.6	77.8	77.6	77.8	77.7	77.8	77.9	< 77	77.6	77.9	< 77	79.1	77.4	77.5	< 77	77.4	< 77	77.7
GCA_000759675.1	*Neobacillus niacini*	**85.2**	**85.1**	**85.4**	**85.4**	**84.6**	< 77	< 77	84.7	< 77	79.4	78.6	83.1	79.1	79.0	78.8	79.0	78.6	78.6	77.5	78.7	83.2	80.5	78.7	< 77	< 77	86.0	79.1	84.4	79.3	< 77	< 77
GCA_036963385.1	*Neobacillus niacini*	**85.2**	**85.2**	**85.5**	**85.6**	**84.7**	< 77	< 77	84.8	< 77	79.3	78.6	83.1	79.0	78.7	78.9	78.8	78.6	78.6	77.4	78.6	83.3	80.7	78.7	< 77	< 77	86.1	79.0	84.6	79.2	< 77	< 77
GCA_017353195.1	*Neobacillus sp. MM2021_6*	**78.4**	**78.2**	**78.4**	**78.3**	**78.2**	< 77	< 77	78.3	< 77	79.1	79.1	78.4	79.4	79.3	79.3	79.2	79.4	79.2	77.8	87.4	78.4	79.2	79.2	< 77	78.3	78.5	79.2	78.2	79.1	< 77	77.7
GCA_023715155.1	*Neobacillus mesonae*	**< 77**	**< 77**	**< 77**	**< 77**	**< 77**	< 77	< 77	< 77	< 77	77.9	< 77	< 77	< 77	77.9	77.9	77.8	77.7	< 77	77.8	78.4	< 77	< 77	78.0	< 77	< 77	< 77	77.7	< 77	77.9	< 77	79.0
GCA_001591665.1	*Neobacillus soli NBRC 102451*	**78.1**	**78.2**	**78.3**	**78.1**	**78.3**	< 77	< 77	78.5	< 77	79.8	78.5	78.4	79.9	78.6	79.3	79.9	78.9	78.6	77.6	80.8	78.3	79.8	78.5	< 77	78.1	78.4	79.9	78.2	80.2	< 77	77.5
GCA_010975035.1	*Neobacillus thermocopriae*	**77.8**	**77.6**	**77.7**	**77.6**	**77.6**	< 77	< 77	77.7	< 77	77.8	77.9	77.7	77.8	77.8	78.1	77.7	77.9	78.4	99.3	77.8	< 77	77.5	77.9	< 77	78.0	77.7	77.8	77.8	77.7	< 77	77.9
GCA_019969725.1	*Neobacillus bataviensis*	**79.0**	**78.7**	**79.1**	**79.0**	**78.5**	< 77	< 77	78.6	< 77	80.2	79.4	78.6	79.1	79.2	79.2	79.0	79.3	79.1	78.2	79.3	78.3	79.9	79.5	< 77	78.7	78.8	79.0	79.2	79.0	< 77	78.1
GCA_036223565.1	*Neobacillus mesonae*	**< 77**	**< 77**	**< 77**	**< 77**	**< 77**	< 77	< 77	< 77	< 77	78.2	< 77	< 77	78.0	78.2	78.1	78.0	78.4	78.1	78.1	78.5	< 77	78.0	< 77	< 77	< 77	< 77	77.9	< 77	77.9	< 77	79.4
GCA_030813055.1	*Neobacillus ginsengisoli*	**79.1**	**78.9**	**78.9**	**78.9**	**78.4**	< 77	< 77	78.7	78.2	79.3	79.2	78.7	79.1	79.0	79.0	79.2	79.1	78.9	77.9	79.0	78.4	79.6	79.5	< 77	78.4	79.1	79.1	79.0	79.1	< 77	< 77
GCA_014656545.1	*Neobacillus kokaensis*	**< 77**	**< 77**	**77.9**	**78.1**	**77.9**	< 77	< 77	78.0	< 77	77.8	< 77	< 77	78.0	78.0	78.2	78.1	78.1	78.2	78.0	78.5	77.9	78.0	78.2	< 77	< 77	< 77	78.1	< 77	78.0	< 77	79.6
GCA_036965485.1	*Neobacillus drentensis*	**78.9**	**78.9**	**79.3**	**79.1**	**78.7**	< 77	< 77	78.8	< 77	83.5	79.6	78.8	85.4	80.0	79.6	85.5	80.1	79.4	77.5	79.6	78.9	84.9	79.5	< 77	78.0	79.1	85.5	79.0	86.2	< 77	< 77
GCA_010614825.1	*Neobacillus sedimentimangrovi*	**77.7**	**77.6**	**77.7**	**77.7**	**77.6**	< 77	< 77	77.5	< 77	77.8	78.1	77.6	77.6	77.9	78.1	77.6	78.0	78.1	95.3	77.9	77.5	77.7	78.1	< 77	78.1	77.7	77.6	77.6	77.8	< 77	77.7
GCA_036962665.1	*Neobacillus niacini*	**82.0**	**82.0**	**81.9**	**82.1**	**82.0**	< 77	< 77	82.2	< 77	78.4	78.1	83.4	78.3	78.4	78.5	78.1	78.4	78.3	< 77	78.6	83.5	78.4	78.1	< 77	< 77	82.8	78.3	81.6	79.0	< 77	< 77
GCA_030316255.1	*Neobacillus mesonae*	**< 77**	**< 77**	**< 77**	**< 77**	**< 77**	< 77	< 77	< 77	< 77	79.0	< 77	< 77	78.7	78.2	78.1	78.0	78.4	< 77	78.1	78.5	< 77	78.0	< 77	< 77	< 77	< 77	77.9	< 77	77.9	< 77	79.4
GCA_036961795.1	*Neobacillus vireti*	**78.7**	**78.4**	**78.7**	**78.6**	**78.1**	< 77	< 77	78.3	< 77	79.6	82.1	78.5	78.9	85.1	79.2	79.2	85.6	83.2	77.8	79.2	78.3	79.2	82.5	< 77	< 77	78.4	79.2	78.6	78.8	< 77	< 77
GCA_030123465.1	*Neobacillus cucumis*	**78.7**	**78.3**	**78.5**	**79.0**	**78.1**	< 77	< 77	78.2	< 77	79.8	78.9	78.7	78.9	78.9	78.6	78.4	78.7	78.7	77.8	78.6	78.4	78.5	79.7	< 77	78.7	79.1	78.4	79.3	78.6	< 77	< 77
GCA_001636315.1	*Neobacillus mesonae*	**< 77**	**< 77**	**< 77**	**< 77**	**< 77**	< 77	< 77	< 77	< 77	78.9	< 77	< 77	78.6	< 77	78.2	77.9	78.2	78.0	78.1	78.6	< 77	77.9	< 77	< 77	< 77	< 77	77.9	< 77	78.1	< 77	79.3
GCA_036965145.1	*Neobacillus drentensis*	**78.8**	**78.7**	**78.9**	**78.9**	**78.5**	< 77	< 77	78.7	< 77	83.9	78.9	78.5	85.2	79.4	80.0	85.2	79.5	79.2	77.8	79.5	78.4	86.3	79.2	< 77	< 77	79.7	85.2	79.1	85.8	< 77	77.6
GCA_004116995.1	*Neobacillus thermocopriae*	**< 77**	**< 77**	**< 77**	**< 77**	**< 77**	< 77	< 77	< 77	< 77	< 77	< 77	< 77	< 77	< 77	< 77	< 77	< 77	< 77	< 77	< 77	< 77	< 77	< 77	< 77	< 77	< 77	< 77	< 77	< 77	< 77	< 77
GCA_001636415.1	*Neobacillus drentensis*	**79.1**	**78.9**	**78.9**	**79.3**	**78.4**	< 77	< 77	78.6	< 77	83.0	79.0	78.8	92.8	79.5	79.3	92.8	79.3	79.0	77.7	79.7	78.9	83.0	79.5	< 77	79.0	79.7	92.9	79.2	96.6	< 77	77.5

GenBank Accession #	Scientific Name	Neobacillus driksii 179-C4-2-HS(T)	Neobacillus driksii 179-J1A1-HS	Neobacillus driksii AT2.8	Neobacillus driksii V4I25	Neobacillus niacini NBRC 15566	Neobacillus terrae	Neobacillus mesonae	Neobacillus niacini	Neobacillus sp. PS3-34	Neobacillus sp. SuZ13	Neobacillus cucumis	Neobacillus vireti	Neobacillus sp. OS1-33	Neobacillus cucumis	Neobacillus drentensis	Neobacillus drentensis	Neobacillus vireti	Neobacillus cucumis	Neobacillus thermocopriae	Neobacillus bataviensis LMG 21833	Neobacillus vireti	Neobacillus drentensis	Neobacillus cucumis	Neobacillus piezotolerans	Neobacillus endophyticus	Neobacillus sp. YX16	Neobacillus drentensis	Neobacillus sp. DY30	Neobacillus drentensis	Neobacillus sp. YIM B06451	Neobacillus paridis
GCA_036965425.1	*Neobacillus drentensis*	78.8	78.6	78.9	78.8	78.6	< 77	< 77	78.7	< 77	79.9	79.3	78.6	79.0	79.2	79.3	78.9	79.4	79.3	78.1	79.3	78.5	80.1	79.4	< 77	78.6	78.7	79.0	78.8	79.1	< 77	78.0
GCA_023714155.1	*Neobacillus sp. MER 74*	78.6	78.7	78.9	78.7	78.5	< 77	< 77	78.5	< 77	90.6	79.6	78.5	82.6	79.8	79.5	82.5	79.8	79.8	77.8	79.1	78.4	85.0	80.0	< 77	< 77	78.9	82.6	78.8	82.6	< 77	< 77
GCA_036965445.1	*Neobacillus niacini*	90.3	90.2	90.3	90.4	86.8	< 77	< 77	86.8	< 77	78.8	78.6	82.4	78.4	78.2	78.5	78.6	78.5	78.4	77.8	78.2	82.4	78.7	78.5	< 77	< 77	84.6	78.5	86.4	78.7	< 77	< 77
GCA_031456445.1	*Neobacillus niacini*	78.4	78.2	78.3	78.5	78.1	< 77	< 77	78.2	< 77	79.0	79.0	78.5	78.8	78.9	78.6	78.7	78.8	78.7	77.6	78.7	78.4	78.8	79.2	< 77	78.2	78.5	78.7	78.7	78.7	< 77	77.6
GCA_003515685.1	*Neobacillus notoginsengisoli*	< 77	< 77	< 77	< 77	< 77	< 77	< 77	< 77	< 77	< 77	< 77	< 77	< 77	< 77	< 77	< 77	< 77	< 77	< 77	< 77	< 77	< 77	< 77	79.2	< 77	< 77	< 77	< 77	< 77	79.1	< 77
GCA_000612665.1	*Neobacillus dielmonensis*	< 77	< 77	< 77	< 77	< 77	< 77	< 77	< 77	< 77	78.1	78.3	< 77	< 77	78.3	77.9	< 77	78.1	78.3	77.8	78.2	< 77	< 77	78.5	< 77	< 77	< 77	< 77	< 77	< 77	< 77	< 77
GCA_001591805.1	*Neobacillus novalis NBRC 102450*	78.2	78.1	78.3	78.4	78.1	< 77	< 77	78.1	< 77	79.2	78.7	78.3	79.5	78.7	79.4	79.4	79.1	78.9	78.0	81.1	78.1	79.4	78.7	< 77	78.2	78.3	79.5	78.3	79.5	< 77	77.9
GCA_030915505.1	*Neobacillus sp. OS1-2*	78.8	78.2	78.5	79.0	78.2	< 77	< 77	78.3	< 77	79.6	79.2	78.4	79.7	79.3	79.3	79.2	79.4	79.2	77.9	87.4	78.3	79.1	79.6	< 77	78.9	79.0	79.3	78.8	79.2	< 77	77.8
GCA_937468385.1	*Neobacillus rhizosphaerae*	78.3	78.3	78.4	78.5	78.2	< 77	< 77	78.4	< 77	79.6	78.9	78.3	79.7	79.0	79.5	79.5	79.1	78.8	77.8	80.3	78.3	79.7	79.0	< 77	78.2	78.6	79.5	78.3	79.8	< 77	78.2
GCA_021109295.1	*Neobacillus sedimentimangrovi*	77.8	77.6	77.8	77.7	77.5	< 77	< 77	77.6	< 77	77.7	78.0	77.6	77.7	78.0	78.1	77.6	78.2	78.1	95.3	77.9	77.5	77.6	78.2	< 77	78.1	77.7	77.6	77.5	77.7	< 77	77.8
GCA_030348765.1	*Neobacillus sp. CF12*	84.1	83.8	84.2	84.3	83.1	< 77	< 77	83.4	< 77	79.5	78.7	82.2	79.7	78.7	78.8	78.9	78.7	78.6	77.7	78.5	82.1	78.8	79.1	< 77	< 77	85.6	78.8	83.9	78.8	< 77	< 77
GCA_001048695.1	*Neobacillus massiliamazoniensis*	< 77	< 77	< 77	78.3	< 77	< 77	< 77	< 77	< 77	78.7	78.6	< 77	78.5	78.3	78.5	78.2	78.3	< 77	77.9	78.5	< 77	78.5	78.8	< 77	78.7	78.4	78.3	< 77	78.5	< 77	< 77
GCA_023702235.1	*Neobacillus pocheonensis*	79.1	78.6	78.6	79.2	78.3	< 77	< 77	78.5	78.8	79.9	79.3	78.4	79.6	79.2	78.9	79.0	79.6	79.2	77.8	79.0	78.4	79.5	80.0	< 77	79.1	79.6	79.0	79.4	79.0	< 77	< 77
GCA_001591445.1	*Neobacillus drentensis NBRC 102427*	78.5	78.8	78.7	78.8	78.4	< 77	< 77	78.4	< 77	82.8	78.8	78.6	92.8	79.3	79.2	92.9	79.2	78.9	77.5	79.7	78.7	83.0	78.8	< 77	78.1	79.2	92.9	78.5	96.6	< 77	< 77
GCA_018343545.2	*Neobacillus citreus*	< 77	< 77	< 77	< 77	< 77	< 77	< 77	< 77	< 77	78.5	78.1	< 77	78.1	78.5	78.3	78.2	78.2	78.7	78.1	78.5	< 77	78.1	78.4	< 77	< 77	< 77	78.2	< 77	78.2	< 77	77.9
GCA_036963125.1	*Neobacillus drentensis*	78.8	78.7	78.7	78.7	78.5	< 77	< 77	78.5	< 77	82.8	79.0	78.3	97.1	79.2	79.2	99.1	79.4	79.0	77.7	79.2	78.2	82.7	79.1	< 77	78.0	78.8	99.0	78.5	93.3	< 77	77.4
GCA_036961895.1	*Neobacillus drentensis*	79.0	78.9	79.5	79.2	78.7	< 77	< 77	78.8	< 77	94.0	80.1	78.7	82.9	79.7	79.3	82.9	80.2	79.6	77.7	79.1	78.5	86.0	80.1	< 77	< 77	79.2	83.0	79.3	83.0	< 77	< 77
GCA_036963445.1	*Neobacillus drentensis*	84.4	84.2	84.5	84.4	83.4	< 77	< 77	83.5	< 77	79.0	78.6	82.5	78.9	78.7	78.7	78.8	78.7	78.4	77.7	78.6	82.6	80.1	78.4	< 77	< 77	95.1	78.8	83.4	79.4	< 77	< 77
GCA_029256785.1	*Neobacillus sp.*	78.7	78.9	78.9	78.8	78.9	< 77	< 77	78.9	< 77	78.0	< 77	78.8	78.4	< 77	< 77	78.5	< 77	77.6	< 77	< 77	78.7	78.1	< 77	< 77	< 77	79.0	78.1	78.5	78.3	< 77	< 77
GCA_018343535.1	*Neobacillus rhizophilus*	< 77	< 77	< 77	< 77	< 77	< 77	< 77	< 77	< 77	78.9	78.5	< 77	78.7	78.7	78.6	78.3	78.5	78.7	78.2	78.7	78.3	78.3	79.0	< 77	78.6	78.5	78.3	< 77	78.5	< 77	78.1
GCA_036962125.1	*Neobacillus drentensis*	78.6	78.5	78.5	78.6	78.2	< 77	< 77	78.3	< 77	83.5	78.9	78.4	82.7	78.9	79.2	83.2	79.0	79.0	77.7	79.1	78.4	83.1	78.8	< 77	78.3	78.7	82.8	78.6	82.4	< 77	< 77
GCA_000820865.2	*Neobacillus jeddahensis*	78.6	78.3	78.4	78.8	78.3	< 77	< 77	78.5	< 77	79.6	79.4	78.4	79.6	79.7	79.4	79.3	79.6	79.6	78.0	80.1	78.3	79.3	79.7	< 77	78.5	78.6	79.3	78.7	79.3	< 77	< 77
GCA_036966985.1	*Neobacillus drentensis*	78.9	78.9	79.5	79.2	78.7	< 77	< 77	78.8	< 77	93.9	80.2	78.6	82.9	79.8	79.3	83.0	80.3	79.7	77.6	79.1	78.6	85.9	80.3	< 77	< 77	79.3	83.1	79.4	83.1	< 77	< 77
GCA_946151075.1	*Neobacillus sp. Marseille-Q6967*	79.8	79.4	79.5	80.1	79.3	< 77	< 77	79.5	< 77	79.6	78.6	79.2	79.1	78.8	78.5	78.4	78.9	78.6	77.8	78.6	79.2	78.6	79.2	< 77	< 77	79.8	78.5	80.0	78.5	< 77	< 77
GCA_036965245.1	*Neobacillus drentensis*	78.7	78.6	78.6	78.7	78.3	< 77	< 77	78.5	< 77	79.3	79.6	78.5	79.4	79.5	80.0	79.4	79.7	79.7	78.3	83.0	78.4	79.7	79.5	< 77	78.5	78.6	79.4	78.5	79.6	< 77	78.3
GCA_036965285.1	*Neobacillus drentensis*	78.8	78.9	79.1	79.1	78.6	< 77	< 77	78.7	< 77	83.7	79.9	78.6	85.4	79.9	79.6	85.5	80.1	79.5	77.7	79.6	78.6	84.8	79.5	< 77	78.2	79.1	85.6	79.0	86.2	< 77	< 77
GCA_030915385.1	*Neobacillus sp. PS3-12*	< 77	< 77	< 77	< 77	< 77	< 77	< 77	< 77	< 77	< 77	79.2	< 77	79.1	< 77	< 77	78.0	< 77	< 77	< 77	< 77	< 77	< 77	79.7	< 77	< 77	< 77	78.1	< 77	< 77	< 77	< 77
GCA_003999735.1	*Neobacillus mesonae*	< 77	< 77	< 77	< 77	< 77	< 77	< 77	< 77	< 77	79.1	< 77	< 77	78.7	< 77	78.1	77.9	< 77	< 77	78.1	78.6	< 77	77.9	< 77	< 77	< 77	< 77	77.9	< 77	78.1	< 77	79.3
GCA_036496045.1	*Neobacillus sp.*	77.9	77.8	77.8	77.9	77.7	< 77	< 77	77.8	< 77	78.0	77.8	77.6	78.1	77.7	77.8	78.1	78.1	77.7	77.0	78.0	77.7	78.2	77.7	< 77	77.4	77.8	78.1	77.6	78.1	< 77	77.1
GCA_023715415.1	*Neobacillus niacini*	83.6	83.5	83.5	83.6	83.1	< 77	< 77	83.1	< 77	78.6	78.2	82.1	78.6	78.3	78.5	78.5	78.2	78.2	77.7	78.2	82.2	78.7	78.4	< 77	< 77	89.8	78.6	83.0	78.9	< 77	< 77
GCA_016908975.1	*Neobacillus cucumis*	78.6	78.5	78.6	78.7	< 77	< 77	< 77	< 77	< 77	79.6	97.7	78.4	78.8	82.8	78.9	78.9	83.1	82.5	77.7	79.0	78.4	79.0	95.4	< 77	78.5	78.6	79.0	78.6	78.9	< 77	< 77
GCA_943193175.1	*Neobacillus muris*	< 77	< 77	< 77	< 77	< 77	< 77	< 77	< 77	< 77	77.8	< 77	< 77	< 77	77.8	77.9	< 77	< 77	78.1	77.7	77.8	< 77	< 77	< 77	< 77	< 77	< 77	< 77	< 77	< 77	< 77	< 77
GCA_036219525.1	*Neobacillus thermocopriae*	77.8	77.6	77.7	77.6	77.7	< 77	< 77	77.9	< 77	77.8	77.9	77.7	77.7	77.7	78.1	77.7	77.9	78.3	100.0	77.9	< 77	77.6	77.8	< 77	78.1	77.6	77.8	77.8	77.6	< 77	77.7
GCA_001636395.1	*Neobacillus novalis*	78.7	78.2	78.4	78.8	78.1	< 77	< 77	78.2	< 77	79.6	78.7	78.5	80.0	78.8	79.4	79.4	79.2	79.0	78.2	81.2	78.2	79.4	79.1	< 77	78.8	78.8	79.6	78.8	79.6	< 77	78.0
GCA_001026695.1	*Neobacillus vireti*	78.4	78.2	78.4	78.4	78.3	< 77	< 77	78.4	< 77	79.3	78.9	78.5	79.6	78.9	79.5	79.4	79.1	79.1	78.2	81.0	78.5	79.3	79.0	< 77	78.4	78.6	79.6	78.5	79.6	< 77	78.0
GCA_038592905.1	*Neobacillus sp. FSL H8-0543*	79.6	79.1	79.3	79.6	79.1	< 77	< 77	79.2	< 77	78.9	< 77	79.3	78.9	78.1	78.4	78.4	< 77	78.2	77.4	78.5	79.2	78.6	< 77	< 77	< 77	80.1	78.5	79.5	78.7	< 77	< 77
GCA_030127045.1	*Neobacillus sp. 114*	< 77	< 77	< 77	< 77	< 77	< 77	< 77	< 77	< 77	79.0	78.5	78.1	79.0	78.6	78.6	78.3	78.5	78.7	78.2	78.7	78.0	78.3	79.3	< 77	78.9	< 77	78.2	< 77	78.5	< 77	78.0
GCA_036962465.1	*Neobacillus vireti*	78.5	78.2	78.5	78.5	78.1	< 77	< 77	78.2	< 77	79.3	82.2	78.6	79.2	85.1	79.3	79.2	85.6	82.8	78.0	79.2	78.6	79.1	82.4	< 77	77.9	78.3	79.3	78.4	78.9	< 77	< 77
GCA_030123445.1	*Neobacillus novalis*	78.9	78.2	78.5	79.0	78.2	< 77	< 77	78.4	< 77	79.7	78.7	78.5	79.9	78.8	79.4	79.4	79.2	79.1	78.0	81.2	78.2	79.4	79.3	< 77	78.9	79.0	79.6	79.0	79.6	< 77	78.1
GCA_021560175.1	*Neobacillus drentensis*	78.9	78.4	78.5	79.0	78.2	< 77	< 77	78.4	< 77	80.0	82.4	78.7	79.6	85.4	79.3	79.4	85.9	82.9	78.0	79.4	78.6	79.0	82.7	< 77	78.8	79.3	79.3	79.1	79.1	< 77	< 77
GCA_002335815.1	*Neobacillus soli*	78.6	78.3	78.5	78.8	78.3	< 77	< 77	78.6	< 77	80.1	78.7	78.6	80.3	78.7	79.2	79.9	78.9	78.7	77.8	80.7	78.5	79.8	79.0	< 77	78.7	79.0	80.0	78.8	80.2	< 77	77.8
GCA_000612625.1	*Neobacillus jeddahensis*	78.3	78.2	78.4	78.4	78.4	< 77	< 77	78.4	< 77	79.3	79.4	78.3	79.4	79.6	79.4	79.2	79.7	79.6	77.8	80.0	78.3	79.1	79.5	< 77	78.1	78.4	79.2	78.5	79.2	< 77	< 77
GCA_030915485.1	*Neobacillus sp. PS3-40*	< 77	< 77	< 77	< 77	< 77	< 77	< 77	< 77	< 77	78.4	< 77	< 77	78.5	< 77	< 77	< 77	< 77	< 77	< 77	< 77	< 77	77.6	78.4	< 77	< 77	< 77	< 77	< 77	77.9	< 77	< 77
GCA_031988165.1	*Neobacillus sp.*	78.3	78.2	78.2	78.4	78.1	< 77	< 77	78.2	< 77	79.5	78.7	78.3	79.6	79.1	79.5	79.6	79.2	78.7	77.7	80.2	78.3	79.6	79.0	< 77	78.4	78.5	79.6	78.3	79.8	< 77	78.0
GCA_030817695.1	*Neobacillus niacini*	88.7	88.5	88.7	88.7	86.9	< 77	< 77	87.0	< 77	79.7	78.7	82.8	79.4	78.9	78.6	78.6	78.8	78.8	77.4	78.5	82.7	78.7	79.5	< 77	< 77	84.8	78.8	87.0	78.9	< 77	< 77
GCA_007828895.1	*Neobacillus bataviensis*	78.6	78.7	79.0	79.0	78.3	< 77	< 77	78.4	< 77	79.9	83.6	78.4	79.3	93.8	79.3	79.6	96.8	84.2	77.7	79.4	78.5	79.3	83.6	< 77	78.4	78.9	79.4	78.9	79.3	< 77	< 77
GCA_030123405.1	*Neobacillus sp. WH10*	79.0	78.5	78.5	79.2	78.3	< 77	< 77	78.4	< 77	79.7	79.4	78.5	79.9	79.6	79.8	79.6	79.6	79.7	78.1	82.7	78.4	79.9	79.8	< 77	78.9	79.1	79.6	79.0	79.7	< 77	78.0
GCA_029240335.1	*Neobacillus sp.*	90.6	90.6	90.8	90.9	86.8	< 77	< 77	86.9	< 77	78.8	78.5	82.6	78.6	78.2	78.4	78.7	78.5	78.1	< 77	77.9	82.5	78.4	78.6	< 77	< 77	84.9	78.6	86.4	78.9	< 77	< 77
GCA_030915425.1	*Neobacillus sp. OS1-32*	< 77	< 77	< 77	< 77	< 77	< 77	< 77	< 77	< 77	78.5	< 77	< 77	78.5	< 77	< 77	< 77	< 77	< 77	77.8	77.8	< 77	< 77	< 77	< 77	79.0	< 77	< 77	< 77	< 77	< 77	98.8
GCA_000768265.1	*Neobacillus niacini*	86.3	86.2	86.3	86.3	99.5	< 77	< 77	99.5	< 77	79.1	78.7	82.9	78.9	78.7	< 77	79.0	78.6	78.8	< 77	79.0	83.1	78.9	78.9	< 77	< 77	83.3	79.1	86.8	78.9	< 77	< 77
GCA_902375305.1	*Neobacillus jeddahensis*	78.3	78.2	78.4	78.4	78.4	< 77	< 77	78.4	< 77	79.3	79.4	78.3	79.4	79.6	79.4	79.2	79.7	79.6	77.8	80.0	78.3	79.1	79.5	< 77	78.1	78.4	79.2	78.5	79.2	< 77	< 77
GCA_019749295.1	*Neobacillus niacini*	83.7	83.8	83.6	83.8	82.9	< 77	< 77	82.9	< 77	78.4	78.3	81.9	78.4	78.4	78.3	78.3	78.3	78.3	77.7	78.1	81.9	78.4	78.2	< 77	< 77	84.9	78.3	83.0	78.4	< 77	< 77
GCA_036965565.1	*Neobacillus drentensis*	78.5	78.4	78.5	78.7	78.4	< 77	< 77	78.5	< 77	79.4	78.9	78.5	79.4	79.3	95.0	79.3	79.3	79.3	78.1	79.4	78.4	79.6	79.0	< 77	78.3	78.9	79.4	78.8	79.5	< 77	< 77
GCA_031457145.1	*Neobacillus niacini*	82.9	82.8	82.9	82.9	82.9	< 77	< 77	83.0	< 77	79.0	< 77	86.1	78.6	78.6	78.7	78.4	78.3	78.4	77.5	78.6	86.0	79.0	78.2	< 77	< 77	83.9	78.4	82.4	79.3	< 77	< 77
GCA_036962325.1	*Neobacillus drentensis*	79.1	79.1	78.5	78.7	78.3	< 77	< 77	78.4	< 77	83.6	78.9	78.4	82.5	78.9	79.2	82.7	79.0	79.1	77.7	79.1	78.4	83.1	78.8	< 77	78.2	78.8	82.6	78.6	82.5	< 77	< 77

Supplemental File S1. ANI matrix of all genomes of *Neobacillus*

GenBank Accession #	Scientific Name	*Neobacillus niacini*	*Neobacillus niacini*	*Neobacillus vireti LMG 21834*	*Neobacillus sp. PS2-9*	*Neobacillus cucumis*	*Neobacillus niacini*	*Neobacillus fumarioli NBRC 102428*	*Neobacillus niacini*	*Neobacillus niacini*	*Neobacillus sp. MM2021_6*	*Neobacillus mesonae*	*Neobacillus soli NBRC 102451*	*Neobacillus thermocopriae*	*Neobacillus bataviensis*	*Neobacillus mesonae*	*Neobacillus ginsengisoli*	*Neobacillus kokaensis*	*Neobacillus drentensis*	*Neobacillus sedimentimangrovi*	*Neobacillus niacini*	*Neobacillus mesonae*	*Neobacillus vireti*	*Neobacillus cucumis*	*Neobacillus mesonae*	*Neobacillus drentensis*	*Neobacillus thermocopriae*	*Neobacillus drentensis*	*Neobacillus drentensis*	*Neobacillus sp. MER 74*	*Neobacillus niacini*	*Neobacillus niacini*
**GCA_030732025.1**	***Neobacillus driksii 179-C4-2-HS(T)***																															
**JBDZYE000000000**	***Neobacillus driksii 179-J1A1-HS***																															
**GCA_013409995.1**	***Neobacillus driksii AT2.8***																															
**GCA_030817595.1**	***Neobacillus driksii V4I25***																															
**GCA_001591505.1**	***Neobacillus niacini NBRC 15566***																															
GCA_011393025.1	*Neobacillus terrae*																															
GCA_023715865.1	*Neobacillus mesonae*																															
GCA_035809615.1	*Neobacillus niacini*																															
GCA_030915465.1	*Neobacillus sp. PS3-34*																															
GCA_030123365.1	*Neobacillus sp. SuZ13*																															
GCA_036223585.1	*Neobacillus cucumis*																															
GCA_036962345.1	*Neobacillus vireti*																															
GCA_030915405.1	*Neobacillus sp. OS1-33*																															
GCA_023715505.1	*Neobacillus cucumis*																															
GCA_036962725.1	*Neobacillus drentensis*																															
GCA_036962365.1	*Neobacillus drentensis*																															
GCA_036965785.1	*Neobacillus vireti*																															
GCA_002860255.1	*Neobacillus cucumis*																															
GCA_036219545.1	*Neobacillus thermocopriae*																															
GCA_000307875.1	*Neobacillus bataviensis LMG 21833*																															
GCA_036961715.1	*Neobacillus vireti*																															
GCA_036962765.1	*Neobacillus drentensis*																															
GCA_031324245.1	*Neobacillus cucumis*																															
GCA_003362805.1	*Neobacillus piezotolerans*																															
GCA_013248975.1	*Neobacillus endophyticus*																															
GCA_030123505.1	*Neobacillus sp. YX16*																															
GCA_036962905.1	*Neobacillus drentensis*																															
GCA_030123065.1	*Neobacillus sp. DY30*																															
GCA_031457825.1	*Neobacillus drentensis*																															
GCA_032667185.1	*Neobacillus sp. YIM B06451*																															
GCA_016765675.1	*Neobacillus paridis*																															
GCA_036962645.1	*Neobacillus niacini*	100.0																														
GCA_023715785.1	*Neobacillus niacini*	< 77	100.0																													
GCA_000508325.2	*Neobacillus vireti LMG 21834*	78.3	78.5	100.0																												
GCA_030915525.1	*Neobacillus sp. PS2-9*	78.3	78.1	79.3	100.0																											
GCA_016107705.1	*Neobacillus cucumis*	78.2	< 77	78.5	78.7	100.0																										
GCA_036961885.1	*Neobacillus niacini*	98.4	< 77	78.3	78.6	78.2	100.0																									
GCA_001591485.1	*Neobacillus fumarioli NBRC 102428*	< 77	77.9	78.0	77.7	77.9	< 77	100.0																								
GCA_000759675.1	*Neobacillus niacini*	83.3	< 77	78.5	78.8	78.5	83.3	77.4	100.0																							
GCA_036963385.1	*Neobacillus niacini*	83.3	< 77	78.5	78.7	78.4	83.4	77.3	96.1	100.0																						
GCA_017353195.1	*Neobacillus sp. MM2021_6*	78.1	77.9	80.1	79.4	78.3	78.1	77.8	78.4	78.4	100.0																					
GCA_023715155.1	*Neobacillus mesonae*	< 77	< 77	78.1	< 77	78.3	< 77	78.0	< 77	< 77	78.0	100.0																				
GCA_001591665.1	*Neobacillus soli NBRC 102451*	78.4	78.1	81.7	79.3	78.4	78.5	77.8	79.0	78.9	79.8	78.0	100.0																			
GCA_010975035.1	*Neobacillus thermocopriae*	77.4	78.1	78.0	78.4	77.9	77.4	77.8	77.4	77.3	77.9	77.9	77.7	100.0																		
GCA_019969725.1	*Neobacillus bataviensis*	78.3	78.4	79.1	79.4	79.0	78.2	78.1	79.0	79.0	79.3	78.3	79.2	78.2	100.0																	
GCA_036223565.1	*Neobacillus mesonae*	< 77	78.3	78.4	78.2	78.0	< 77	77.9	78.2	< 77	78.2	80.0	78.3	78.0	78.6	100.0																
GCA_030813055.1	*Neobacillus ginsengisoli*	78.4	< 77	78.9	79.1	79.5	78.5	78.0	79.4	79.2	78.6	< 77	79.1	77.9	79.1	78.2	100.0															
GCA_014656545.1	*Neobacillus kokaensis*	< 77	78.2	78.4	78.3	78.2	< 77	78.0	78.2	78.3	78.2	80.1	78.3	77.9	78.5	84.2	78.1	100.0														
GCA_036965485.1	*Neobacillus drentensis*	78.4	78.1	79.4	79.5	78.6	78.6	77.5	79.6	79.6	79.1	77.6	80.4	77.5	79.5	78.0	79.4	78.1	100.0													
GCA_010614825.1	*Neobacillus sedimentimangrovi*	77.6	78.2	78.1	78.2	77.7	77.4	77.7	77.5	77.4	77.9	78.0	77.6	95.4	78.3	78.0	77.9	78.0	77.6	100.0												
GCA_036962665.1	*Neobacillus niacini*	86.5	< 77	78.6	78.6	78.4	86.5	< 77	83.6	83.8	78.3	< 77	78.5	77.4	78.4	< 77	78.6	77.9	78.6	77.5	100.0											
GCA_030316255.1	*Neobacillus mesonae*	< 77	78.4	78.4	78.8	78.2	< 77	77.9	78.0	< 77	78.3	79.9	78.3	78.2	78.7	98.8	78.3	84.3	78.0	78.1	< 77	100.0										
GCA_036961795.1	*Neobacillus vireti*	< 77	< 77	78.8	79.3	78.7	77.9	77.6	78.4	78.3	79.1	< 77	78.7	77.8	79.2	< 77	78.8	< 77	79.5	77.9	77.9	< 77	100.0									
GCA_030123465.1	*Neobacillus cucumis*	78.2	< 77	78.6	79.3	97.7	78.3	77.7	78.5	78.4	78.4	78.1	78.4	78.0	79.0	78.2	79.4	78.3	78.7	77.8	78.5	79.0	78.7	100.0								
GCA_001636315.1	*Neobacillus mesonae*	< 77	78.5	78.5	78.8	78.2	< 77	78.0	< 77	< 77	78.2	80.1	78.4	78.1	78.6	97.5	78.3	84.3	78.2	78.1	< 77	97.9	< 77	79.0	100.0							
GCA_036965145.1	*Neobacillus drentensis*	78.2	< 77	79.5	80.0	78.7	78.2	77.5	80.8	80.8	79.2	77.8	80.7	77.8	79.7	78.4	79.9	78.3	88.0	77.6	78.6	78.4	79.1	78.7	78.4	100.0						
GCA_004116995.1	*Neobacillus thermocopriae*	< 77	< 77	< 77	< 77	< 77	< 77	< 77	< 77	< 77	< 77	< 77	< 77	< 77	< 77	< 77	< 77	< 77	< 77	< 77	< 77	< 77	< 77	< 77	< 77	< 77	100.0					
GCA_001636415.1	*Neobacillus drentensis*	78.9	78.1	79.5	79.6	78.6	79.0	77.6	79.0	79.1	79.1	77.9	80.1	77.8	79.2	78.1	79.0	77.9	85.8	77.7	79.0	78.6	79.0	79.3	78.8	85.4	< 77	100.0				

GenBank Accession #	Scientific Name	*Neobacillus niacini*	*Neobacillus niacini*	*Neobacillus vireti LMG 21834*	*Neobacillus sp. PS2-9*	*Neobacillus cucumis*	*Neobacillus niacini*	*Neobacillus fumarioli NBRC 102428*	*Neobacillus niacini*	*Neobacillus niacini*	*Neobacillus sp. MM2021_6*	*Neobacillus mesonae*	*Neobacillus soli NBRC 102451*	*Neobacillus thermocopriae*	*Neobacillus bataviensis*	*Neobacillus mesonae*	*Neobacillus ginsengisoli*	*Neobacillus kokaensis*	*Neobacillus drentensis*	*Neobacillus sedimentimangrovi*	*Neobacillus niacini*	*Neobacillus mesonae*	*Neobacillus vireti*	*Neobacillus cucumis*	*Neobacillus mesonae*	*Neobacillus drentensis*	*Neobacillus thermocopriae*	*Neobacillus drentensis*	*Neobacillus drentensis*	*Neobacillus sp. MER 74*	*Neobacillus niacini*	*Neobacillus niacini*
GCA_036965425.1	*Neobacillus drentensis*	78.4	78.6	79.2	79.4	79.1	78.6	77.9	79.2	79.2	79.3	78.2	79.1	78.1	95.0	78.6	79.1	78.4	79.9	78.1	78.5	78.7	79.1	79.1	78.7	79.5	< 77	79.1	100.0			
GCA_023714155.1	*Neobacillus sp. MER 74*	78.3	< 77	79.1	79.6	78.4	78.3	77.5	79.0	79.1	79.1	< 77	79.6	77.8	79.5	78.0	79.3	77.8	83.2	77.7	78.2	78.0	79.5	78.5	78.0	83.5	< 77	82.6	79.4	100.0		
GCA_036965445.1	*Neobacillus niacini*	82.0	< 77	< 77	78.6	78.3	82.1	< 77	85.6	85.6	78.4	< 77	78.2	77.8	78.5	< 77	78.8	< 77	78.5	77.8	82.1	< 77	78.5	78.4	< 77	78.5	< 77	78.5	78.4	78.5	100.0	
GCA_031456445.1	*Neobacillus niacini*	78.1	< 77	78.6	78.6	87.5	78.2	77.6	79.0	78.7	78.5	< 77	78.4	77.5	79.2	78.2	79.5	78.1	78.7	77.9	78.4	78.4	78.6	87.3	78.4	79.1	< 77	78.6	79.3	78.6	78.4	100.0
GCA_003515685.1	*Neobacillus notoginsengisoli*	< 77	< 77	< 77	< 77	< 77	< 77	< 77	< 77	< 77	< 77	< 77	< 77	< 77	< 77	< 77	< 77	< 77	< 77	< 77	< 77	< 77	< 77	< 77	< 77	< 77	< 77	< 77	< 77	< 77	< 77	< 77
GCA_000612665.1	*Neobacillus dielmonensis*	< 77	78.3	< 77	< 77	< 77	< 77	77.7	< 77	< 77	< 77	78.1	< 77	77.9	78.5	78.1	< 77	78.1	< 77	77.6	< 77	78.1	< 77	< 77	78.2	< 77	< 77	< 77	78.1	< 77	< 77	< 77
GCA_001591805.1	*Neobacillus novalis NBRC 102450*	78.4	78.3	90.3	79.3	78.6	78.3	78.0	78.5	78.5	80.3	78.2	82.8	78.1	79.1	78.5	79.1	78.6	79.7	78.0	78.6	78.5	78.9	78.6	78.7	80.0	< 77	79.5	79.2	79.1	78.2	78.5
GCA_030915505.1	*Neobacillus sp. OS1-2*	78.1	78.0	80.2	79.7	78.4	78.1	77.8	78.5	78.4	97.5	78.1	79.8	78.1	79.3	78.1	78.7	78.2	79.2	78.0	78.4	78.7	79.1	78.8	78.6	79.5	< 77	79.4	79.2	79.1	78.4	78.6
GCA_937468385.1	*Neobacillus rhizosphaerae*	78.3	78.0	82.0	79.5	78.5	78.3	77.8	79.0	79.1	79.8	78.1	80.7	77.8	79.3	78.4	79.2	78.3	80.0	78.0	78.5	78.6	79.0	78.7	78.5	80.8	< 77	79.5	79.4	79.3	78.3	78.8
GCA_021109295.1	*Neobacillus sedimentimangrovi*	77.5	78.1	78.1	78.1	77.7	77.3	77.7	77.6	77.3	77.9	77.9	77.7	95.5	78.4	78.1	77.9	78.1	77.6	99.3	< 77	78.2	77.9	77.9	78.1	77.6	< 77	77.7	78.3	77.8	78.0	77.8
GCA_030348765.1	*Neobacillus sp. CF12*	82.2	< 77	78.2	79.6	78.5	82.2	77.3	84.9	85.2	78.4	< 77	78.3	77.8	78.9	< 77	78.9	< 77	79.1	77.6	82.3	< 77	78.5	78.9	< 77	79.2	< 77	79.2	78.7	78.8	84.1	78.7
GCA_001048695.1	*Neobacillus massiliamazoniensis*	< 77	< 77	78.3	78.8	79.1	< 77	77.6	78.6	78.6	78.2	< 77	78.5	77.8	78.8	< 77	79.8	< 77	78.6	77.8	78.0	78.4	< 77	79.2	< 77	79.2	< 77	78.5	78.9	78.4	< 77	78.9
GCA_023702235.1	*Neobacillus pocheonensis*	78.5	< 77	78.8	79.6	79.3	78.3	77.9	79.2	79.3	78.7	< 77	79.0	77.9	79.2	< 77	85.9	< 77	79.9	77.9	78.7	< 77	79.1	79.7	< 77	80.0	< 77	79.4	79.0	79.3	78.5	79.5
GCA_001591445.1	*Neobacillus drentensis NBRC 102427*	78.8	78.0	79.5	79.2	78.5	78.9	77.7	79.0	79.0	79.0	77.8	80.2	77.5	79.1	77.8	78.9	77.9	85.8	77.6	78.8	77.8	78.9	78.6	78.0	85.3	< 77	100.0	79.0	82.5	78.5	78.5
GCA_018343545.2	*Neobacillus citreus*	< 77	79.2	78.6	78.4	78.6	< 77	77.6	< 77	< 77	78.2	78.1	78.2	78.3	78.9	78.7	78.2	78.5	78.3	78.2	< 77	78.7	78.1	78.6	79.0	78.3	< 77	78.4	79.0	78.4	< 77	78.4
GCA_036963125.1	*Neobacillus drentensis*	78.4	77.8	79.4	79.3	78.4	78.4	77.6	78.9	78.8	79.2	77.7	79.9	77.6	78.9	77.9	79.0	78.0	85.4	77.4	78.2	77.9	79.3	78.4	77.9	85.2	< 77	92.8	78.9	82.5	78.4	78.6
GCA_036961895.1	*Neobacillus drentensis*	78.2	< 77	79.2	79.5	78.8	78.4	77.5	79.4	79.5	79.0	< 77	79.9	77.7	80.3	78.0	79.1	77.9	83.8	77.7	78.5	78.0	79.5	78.8	< 77	84.0	< 77	82.9	80.2	90.8	78.7	78.7
GCA_036963445.1	*Neobacillus drentensis*	82.6	< 77	78.3	78.7	78.2	82.7	< 77	86.5	86.5	78.6	< 77	78.5	77.7	78.7	< 77	79.2	< 77	80.0	77.7	82.9	< 77	78.4	78.2	< 77	79.4	< 77	79.2	78.9	78.9	84.6	78.5
GCA_029256785.1	*Neobacillus sp.*	79.0	< 77	77.9	< 77	< 77	79.0	< 77	79.0	79.0	< 77	< 77	77.9	< 77	78.0	< 77	78.2	< 77	< 77	< 77	79.0	< 77	< 77	< 77	< 77	78.2	< 77	78.1	78.1	78.0	78.8	< 77
GCA_018343535.1	*Neobacillus rhizophilus*	< 77	79.4	78.8	78.8	78.9	78.1	77.7	< 77	< 77	78.2	78.4	78.3	78.4	79.1	79.0	78.4	78.6	78.6	78.4	78.1	79.3	78.4	79.0	79.4	78.7	< 77	78.6	79.1	78.7	< 77	78.6
GCA_036962125.1	*Neobacillus drentensis*	78.2	< 77	79.1	79.2	78.6	78.2	77.6	78.6	78.6	79.1	< 77	79.6	77.8	79.1	78.0	78.8	78.0	82.6	77.8	78.3	78.0	78.9	78.6	78.0	82.9	< 77	82.2	79.2	83.0	78.4	78.3
GCA_000820865.2	*Neobacillus jeddahensis*	78.4	78.2	79.5	79.6	78.6	78.4	78.0	78.9	78.6	79.9	78.1	79.4	78.1	79.1	78.2	78.9	78.1	79.5	78.1	78.3	78.4	79.6	78.8	78.5	79.6	< 77	79.3	79.2	79.3	78.5	78.7
GCA_036966985.1	*Neobacillus drentensis*	78.2	< 77	79.3	79.5	78.6	78.3	77.5	79.3	79.6	79.0	< 77	79.8	77.7	80.2	77.9	79.2	77.9	83.7	77.7	78.7	78.0	79.9	78.6	78.1	84.1	< 77	83.0	80.1	90.7	78.7	78.6
GCA_946151075.1	*Neobacillus sp. Marseille-Q6967*	79.1	< 77	78.4	79.2	78.3	79.0	77.6	79.8	79.8	78.4	< 77	78.3	78.0	78.8	< 77	78.9	< 77	78.8	77.9	79.3	< 77	78.6	79.1	< 77	79.1	< 77	79.3	78.8	78.7	79.3	78.5
GCA_036965245.1	*Neobacillus drentensis*	78.4	78.2	80.9	79.8	78.8	78.3	78.0	79.1	79.3	82.2	78.2	80.3	78.4	79.7	78.7	79.3	78.6	79.7	78.4	78.5	78.6	79.3	78.7	78.6	80.8	< 77	79.5	79.7	79.2	78.7	79.0
GCA_036965285.1	*Neobacillus drentensis*	78.4	77.8	79.3	79.6	78.6	78.4	77.6	80.0	80.0	79.1	77.7	80.3	77.7	79.6	78.1	79.5	78.2	96.9	77.9	78.7	78.0	79.3	78.8	78.2	88.2	< 77	85.8	79.7	83.2	78.5	78.9
GCA_030915385.1	*Neobacillus sp. PS3-12*	< 77	< 77	< 77	< 77	< 77	< 77	77.4	< 77	< 77	< 77	< 77	< 77	< 77	< 77	< 77	78.5	< 77	< 77	< 77	< 77	< 77	< 77	79.0	< 77	< 77	< 77	< 77	< 77	< 77	< 77	< 77
GCA_003999735.1	*Neobacillus mesonae*	< 77	78.4	78.5	78.8	78.0	< 77	77.9	< 77	< 77	78.2	80.2	78.4	78.1	78.6	97.4	78.3	84.4	78.1	78.1	< 77	97.8	< 77	78.8	99.0	78.3	< 77	78.6	78.6	78.0	< 77	78.3
GCA_036496045.1	*Neobacillus sp.*	77.9	77.1	78.8	78.0	77.9	78.0	< 77	78.0	78.2	78.1	77.1	78.4	< 77	78.0	77.1	78.5	77.5	78.3	77.1	77.8	77.1	77.8	77.9	77.4	78.8	< 77	78.4	77.9	78.2	77.4	78.1
GCA_023715415.1	*Neobacillus niacini*	82.3	< 77	78.3	78.9	78.3	82.2	< 77	84.6	84.7	78.4	< 77	78.1	77.7	78.6	< 77	78.7	< 77	78.6	77.8	82.4	< 77	78.2	78.3	< 77	78.7	< 77	78.8	78.5	78.3	83.9	78.2
GCA_016908975.1	*Neobacillus cucumis*	< 77	< 77	78.7	79.0	78.7	< 77	77.8	78.5	78.5	79.1	< 77	78.5	77.9	79.3	< 77	79.0	78.1	79.1	78.1	78.0	< 77	82.1	79.1	< 77	79.0	< 77	78.8	79.3	79.4	78.5	78.9
GCA_943193175.1	*Neobacillus muris*	< 77	78.3	< 77	< 77	< 77	< 77	77.9	< 77	< 77	78.0	< 77	< 77	77.9	78.4	78.0	< 77	78.0	< 77	78.0	< 77	78.0	< 77	< 77	77.9	77.9	< 77	< 77	78.3	< 77	< 77	< 77
GCA_036219525.1	*Neobacillus thermocopriae*	77.6	78.0	78.0	78.2	77.8	77.5	77.9	77.5	77.4	77.8	77.7	77.7	99.3	78.2	78.2	77.9	78.0	77.5	95.4	< 77	78.1	77.8	77.9	78.1	77.9	< 77	77.8	78.1	77.7	77.7	77.6
GCA_001636395.1	*Neobacillus novalis*	78.5	78.3	90.3	79.6	78.7	78.5	78.0	78.6	78.6	80.4	78.2	82.8	78.3	79.3	78.5	79.1	78.6	79.8	78.1	78.7	78.9	78.9	79.0	79.0	80.1	< 77	79.9	79.4	79.3	78.3	78.6
GCA_001026695.1	*Neobacillus vireti*	78.4	78.5	100.0	79.4	78.6	78.4	78.1	78.6	78.5	80.2	78.2	81.7	78.3	79.3	78.5	79.0	78.4	79.5	78.2	78.6	78.6	78.9	78.8	78.8	79.7	< 77	79.7	79.3	79.2	78.3	78.7
GCA_038592905.1	*Neobacillus sp. FSL H8-0543*	79.7	< 77	78.4	79.1	78.1	79.7	< 77	79.8	79.8	78.1	< 77	78.3	77.7	78.3	< 77	78.8	< 77	78.7	77.4	80.1	< 77	< 77	78.9	< 77	79.0	< 77	79.0	78.3	78.5	79.0	78.1
GCA_030127045.1	*Neobacillus sp. 114*	< 77	79.5	78.8	78.9	78.7	< 77	77.6	< 77	78.1	78.2	78.4	78.4	78.4	79.0	79.0	78.4	78.7	78.5	78.5	78.0	79.4	78.1	79.4	79.6	78.6	< 77	78.8	79.1	78.7	< 77	78.6
GCA_036962465.1	*Neobacillus vireti*	78.3	< 77	79.0	79.3	78.5	78.2	77.7	78.4	78.3	79.2	< 77	78.8	78.0	79.2	78.2	78.8	78.2	79.0	77.9	78.2	< 77	83.8	78.7	78.1	79.2	< 77	79.0	79.2	79.3	78.4	78.6
GCA_030123445.1	*Neobacillus novalis*	78.4	78.3	90.3	79.7	78.7	78.4	77.9	78.7	78.6	80.4	78.2	82.8	78.1	79.3	78.5	79.1	78.6	79.8	78.1	78.6	78.9	79.0	79.2	79.1	80.1	< 77	80.0	79.4	79.3	78.2	78.7
GCA_021560175.1	*Neobacillus drentensis*	78.3	< 77	78.9	79.7	78.9	78.3	77.6	78.5	78.4	79.2	78.0	78.8	78.0	79.2	78.2	78.9	78.3	79.2	77.9	78.2	78.9	83.9	79.5	78.9	79.2	< 77	79.7	79.2	79.4	78.3	78.6
GCA_002335815.1	*Neobacillus soli*	78.5	78.2	81.7	79.7	78.5	78.6	77.9	79.1	78.9	80.0	78.2	100.0	77.9	79.3	78.3	79.2	78.3	80.4	77.8	78.5	78.7	78.8	78.8	78.8	80.8	< 77	80.5	79.1	79.6	78.1	78.5
GCA_000612625.1	*Neobacillus jeddahensis*	78.3	78.1	79.5	79.4	78.5	78.4	77.9	78.8	78.6	79.9	78.1	79.5	77.9	78.9	78.0	78.8	78.0	79.4	77.8	78.3	78.0	79.5	78.5	78.1	79.5	< 77	79.2	79.0	79.2	78.4	78.7
GCA_030915485.1	*Neobacillus sp. PS3-40*	< 77	< 77	< 77	78.5	77.6	< 77	< 77	< 77	< 77	< 77	< 77	77.6	< 77	< 77	< 77	78.2	< 77	< 77	< 77	< 77	< 77	< 77	78.2	< 77	78.0	< 77	78.4	< 77	< 77	< 77	77.9
GCA_031988165.1	*Neobacillus sp.*	78.3	77.9	81.8	79.6	78.6	78.3	77.8	78.8	78.9	79.9	78.0	80.6	77.8	79.3	78.2	79.2	78.3	80.1	78.0	78.4	78.4	79.0	78.6	78.3	80.6	< 77	79.5	79.3	79.3	78.3	78.8
GCA_030817695.1	*Neobacillus niacini*	82.2	< 77	78.3	79.6	78.3	82.3	77.4	85.5	85.6	78.6	< 77	78.4	77.6	78.8	< 77	79.1	< 77	79.1	77.7	82.2	< 77	78.8	79.0	< 77	79.0	< 77	79.3	78.6	78.9	88.3	78.4
GCA_007828895.1	*Neobacillus bataviensis*	78.0	< 77	78.9	79.4	78.8	78.1	77.7	78.7	78.6	79.4	77.7	78.8	77.7	79.5	78.3	79.3	78.2	80.2	77.9	78.3	78.4	85.6	78.9	78.3	79.5	< 77	79.4	79.5	80.2	78.4	78.9
GCA_030123405.1	*Neobacillus sp. WH10*	78.4	78.3	80.8	80.1	78.7	78.4	78.0	79.3	79.2	82.0	78.1	80.2	78.1	79.7	78.5	79.3	78.4	79.7	78.2	78.5	79.1	79.3	79.2	79.1	80.6	< 77	79.8	79.8	79.3	78.4	78.9
GCA_029240335.1	*Neobacillus sp.*	82.2	< 77	78.0	78.2	77.9	82.2	< 77	85.6	85.8	77.8	< 77	78.0	< 77	78.3	< 77	78.5	< 77	78.4	< 77	82.2	< 77	78.7	77.9	< 77	78.2	< 77	78.7	78.2	78.4	95.3	78.3
GCA_030915425.1	*Neobacillus sp. OS1-32*	< 77	78.1	77.9	< 77	< 77	< 77	77.9	< 77	< 77	77.8	79.0	77.6	78.0	78.2	79.5	< 77	79.6	< 77	77.7	< 77	79.7	< 77	78.3	79.7	77.7	< 77	78.2	78.1	< 77	< 77	77.6
GCA_000768265.1	*Neobacillus niacini*	82.2	< 77	78.6	78.9	78.8	82.2	< 77	84.4	84.5	78.7	< 77	78.9	< 77	78.7	< 77	78.8	< 77	78.9	< 77	82.2	77.9	78.6	78.8	< 77	79.1	< 77	78.7	79.1	79.1	86.6	78.6
GCA_902375305.1	*Neobacillus jeddahensis*	78.3	78.1	79.5	79.4	78.5	78.4	77.9	78.8	78.6	79.9	78.1	79.5	77.9	78.9	78.0	78.8	78.0	79.4	77.8	78.3	78.0	79.5	78.5	78.1	79.5	< 77	79.2	79.0	79.2	78.4	78.7
GCA_019749295.1	*Neobacillus niacini*	81.9	< 77	78.3	78.5	78.1	81.9	77.2	83.9	84.0	78.2	< 77	78.2	77.7	78.4	< 77	78.7	< 77	78.6	77.5	81.9	< 77	78.3	78.2	< 77	78.5	< 77	78.4	78.5	78.4	83.7	78.3
GCA_036965565.1	*Neobacillus drentensis*	78.4	77.9	79.3	94.7	78.7	78.6	77.7	78.8	78.8	79.5	78.0	79.3	78.0	79.2	78.2	79.0	78.2	79.5	78.1	78.6	78.3	79.3	78.7	78.2	79.9	< 77	79.3	79.3	79.4	78.6	78.5
GCA_031457145.1	*Neobacillus niacini*	84.8	< 77	78.3	78.8	78.5	84.9	< 77	84.8	84.8	78.5	< 77	78.9	77.5	78.5	< 77	78.9	< 77	79.2	77.5	85.5	< 77	78.1	78.6	< 77	79.0	< 77	79.3	78.6	78.6	83.2	78.6
GCA_036962325.1	*Neobacillus drentensis*	78.2	77.9	79.1	79.2	78.6	78.4	77.6	78.6	78.6	79.0	77.6	79.7	77.7	79.2	77.9	78.8	77.9	82.6	77.9	78.4	77.9	78.8	78.4	78.0	83.0	< 77	82.1	79.2	83.1	78.5	78.4

Supplemental File S1. ANI matrix of all genomes of *Neobacillus* [illegible]

GenBank Accession #	Scientific Name	*Neobacillus notoginsengisoli*	*Neobacillus dielmonensis*	*Neobacillus novalis NBRC 102450*	*Neobacillus sp. OS1-2*	*Neobacillus rhizosphaerae*	*Neobacillus sedimentimangrovi*	*Neobacillus sp. CF12*	*Neobacillus massiliamazoniensis*	*Neobacillus pocheonensis*	*Neobacillus drentensis NBRC 102427*	*Neobacillus citreus*	*Neobacillus drentensis*	*Neobacillus drentensis*	*Neobacillus drentensis*	*Neobacillus sp.*	*Neobacillus rhizophilus*	*Neobacillus drentensis*	*Neobacillus jeddahensis*	*Neobacillus drentensis*	*Neobacillus sp. Marseille-Q6967*	*Neobacillus drentensis*	*Neobacillus drentensis*	*Neobacillus sp. PS3-12*	*Neobacillus mesonae*	*Neobacillus sp.*	*Neobacillus niacini*	*Neobacillus cucumis*	*Neobacillus muris*	*Neobacillus thermocopriae*	*Neobacillus novalis*	*Neobacillus vireti*
**GCA_030732025.1**	***Neobacillus driksii 179-C4-2-HS(T)***																															
**JBDZYE000000000**	***Neobacillus driksii 179-J1A1-HS***																															
**GCA_013409995.1**	***Neobacillus driksii AT2.8***																															
**GCA_030817595.1**	***Neobacillus driksii V4I25***																															
**GCA_001591505.1**	***Neobacillus niacini NBRC 15566***																															
GCA_011393025.1	*Neobacillus terrae*																															
GCA_023715865.1	*Neobacillus mesonae*																															
GCA_035809615.1	*Neobacillus niacini*																															
GCA_030915465.1	*Neobacillus sp. PS3-34*																															
GCA_030123365.1	*Neobacillus sp. SuZ13*																															
GCA_036223585.1	*Neobacillus cucumis*																															
GCA_036962345.1	*Neobacillus vireti*																															
GCA_030915405.1	*Neobacillus sp. OS1-33*																															
GCA_023715505.1	*Neobacillus cucumis*																															
GCA_036962725.1	*Neobacillus drentensis*																															
GCA_036962365.1	*Neobacillus drentensis*																															
GCA_036965785.1	*Neobacillus vireti*																															
GCA_002860255.1	*Neobacillus cucumis*																															
GCA_036219545.1	*Neobacillus thermocopriae*																															
GCA_000307875.1	*Neobacillus bataviensis LMG 21833*																															
GCA_036961715.1	*Neobacillus vireti*																															
GCA_036962765.1	*Neobacillus drentensis*																															
GCA_031324245.1	*Neobacillus cucumis*																															
GCA_003362805.1	*Neobacillus piezotolerans*																															
GCA_013248975.1	*Neobacillus endophyticus*																															
GCA_030123505.1	*Neobacillus sp. YX16*																															
GCA_036962905.1	*Neobacillus drentensis*																															
GCA_030123065.1	*Neobacillus sp. DY30*																															
GCA_031457825.1	*Neobacillus drentensis*																															
GCA_032667185.1	*Neobacillus sp. YIM B06451*																															
GCA_016765675.1	*Neobacillus paridis*																															
GCA_036962645.1	*Neobacillus niacini*																															
GCA_023715785.1	*Neobacillus niacini*																															
GCA_000508325.2	*Neobacillus vireti LMG 21834*																															
GCA_030915525.1	*Neobacillus sp. PS2-9*																															
GCA_016107705.1	*Neobacillus cucumis*																															
GCA_036961885.1	*Neobacillus niacini*																															
GCA_001591485.1	*Neobacillus fumarioli NBRC 102428*																															
GCA_000759675.1	*Neobacillus niacini*																															
GCA_036963385.1	*Neobacillus niacini*																															
GCA_017353195.1	*Neobacillus sp. MM2021_6*																															
GCA_023715155.1	*Neobacillus mesonae*																															
GCA_001591665.1	*Neobacillus soli NBRC 102451*																															
GCA_010975035.1	*Neobacillus thermocopriae*																															
GCA_019969725.1	*Neobacillus bataviensis*																															
GCA_036223565.1	*Neobacillus mesonae*																															
GCA_030813055.1	*Neobacillus ginsengisoli*																															
GCA_014656545.1	*Neobacillus kokaensis*																															
GCA_036965485.1	*Neobacillus drentensis*																															
GCA_010614825.1	*Neobacillus sedimentimangrovi*																															
GCA_036962665.1	*Neobacillus niacini*																															
GCA_030316255.1	*Neobacillus mesonae*																															
GCA_036961795.1	*Neobacillus vireti*																															
GCA_030123465.1	*Neobacillus cucumis*																															
GCA_001636315.1	*Neobacillus mesonae*																															
GCA_036965145.1	*Neobacillus drentensis*																															
GCA_004116995.1	*Neobacillus thermocopriae*																															
GCA_001636415.1	*Neobacillus drentensis*																															

GenBank Accession #	Scientific Name	*Neobacillus notoginsengisoli*	*Neobacillus dielmonensis*	*Neobacillus novalis NBRC 102450*	*Neobacillus sp. OS1-2*	*Neobacillus rhizosphaerae*	*Neobacillus sedimentimangrovi*	*Neobacillus sp. CF12*	*Neobacillus massiliamazoniensis*	*Neobacillus pocheonensis*	*Neobacillus drentensis NBRC 102427*	*Neobacillus citreus*	*Neobacillus drentensis*	*Neobacillus drentensis*	*Neobacillus drentensis*	*Neobacillus sp.*	*Neobacillus rhizophilus*	*Neobacillus drentensis*	*Neobacillus jeddahensis*	*Neobacillus drentensis*	*Neobacillus sp. Marseille-Q6967*	*Neobacillus drentensis*	*Neobacillus drentensis*	*Neobacillus sp. PS3-12*	*Neobacillus mesonae*	*Neobacillus sp.*	*Neobacillus niacini*	*Neobacillus cucumis*	*Neobacillus muris*	*Neobacillus thermocopriae*	*Neobacillus novalis*	*Neobacillus vireti*
GCA_036965425.1	*Neobacillus drentensis*																															
GCA_023714155.1	*Neobacillus sp. MER 74*																															
GCA_036965445.1	*Neobacillus niacini*																															
GCA_031456445.1	*Neobacillus niacini*																															
GCA_003515685.1	*Neobacillus notoginsengisoli*	100.0																														
GCA_000612665.1	*Neobacillus dielmonensis*	< 77	100.0																													
GCA_001591805.1	*Neobacillus novalis NBRC 102450*	< 77	78.2	100.0																												
GCA_030915505.1	*Neobacillus sp. OS1-2*	< 77	78.2	80.2	100.0																											
GCA_937468385.1	*Neobacillus rhizosphaerae*	< 77	78.1	82.3	79.7	100.0																										
GCA_021109295.1	*Neobacillus sedimentimangrovi*	< 77	77.7	77.9	77.8	77.9	100.0																									
GCA_030348765.1	*Neobacillus sp. CF12*	< 77	< 77	78.4	78.8	78.6	77.5	100.0																								
GCA_001048695.1	*Neobacillus massiliamazoniensis*	< 77	< 77	78.7	78.3	79.2	77.7	< 77	100.0																							
GCA_023702235.1	*Neobacillus pocheonensis*	< 77	< 77	79.2	79.1	79.3	77.9	79.3	79.5	100.0																						
GCA_001591445.1	*Neobacillus drentensis NBRC 102427*	< 77	< 77	79.5	79.0	79.4	77.6	78.5	78.2	78.9	100.0																					
GCA_018343545.2	*Neobacillus citreus*	< 77	78.4	78.4	78.2	78.4	78.2	< 77	< 77	77.9	78.4	100.0																				
GCA_036963125.1	*Neobacillus drentensis*	< 77	< 77	79.5	79.2	79.4	77.4	78.8	78.2	79.0	92.8	78.1	100.0																			
GCA_036961895.1	*Neobacillus drentensis*	< 77	< 77	79.3	79.0	79.7	77.7	79.0	78.3	79.3	82.8	78.4	82.9	100.0																		
GCA_036963445.1	*Neobacillus drentensis*	< 77	< 77	78.4	78.5	78.4	77.6	85.4	78.1	78.9	79.1	77.9	78.9	79.0	100.0																	
GCA_029256785.1	*Neobacillus sp.*	< 77	< 77	78.0	78.0	78.2	< 77	78.8	< 77	78.2	78.2	< 77	78.4	< 77	79.1	100.0																
GCA_018343535.1	*Neobacillus rhizophilus*	< 77	78.5	78.6	78.4	78.6	78.4	< 77	78.5	< 77	78.4	93.4	78.2	78.4	78.2	< 77	100.0															
GCA_036962125.1	*Neobacillus drentensis*	< 77	< 77	79.1	79.1	79.3	77.8	78.9	78.1	78.7	82.1	78.2	83.2	83.7	78.5	78.2	78.2	100.0														
GCA_000820865.2	*Neobacillus jeddahensis*	< 77	78.4	79.6	80.1	79.6	78.1	78.8	78.3	79.1	79.1	78.2	79.4	79.4	78.5	< 77	78.6	79.3	100.0													
GCA_036966985.1	*Neobacillus drentensis*	< 77	< 77	79.2	79.1	79.7	77.7	79.0	78.2	79.5	82.9	78.3	83.0	99.3	79.0	< 77	78.2	83.7	79.3	100.0												
GCA_946151075.1	*Neobacillus sp. Marseille-Q6967*	< 77	< 77	78.4	78.8	78.5	77.9	80.1	< 77	79.2	78.6	< 77	78.3	78.9	79.3	78.6	78.5	78.4	78.7	79.0	100.0											
GCA_036965245.1	*Neobacillus drentensis*	< 77	78.4	81.1	82.1	82.8	78.3	78.7	79.5	79.2	79.4	78.5	79.3	79.4	78.5	78.0	78.9	79.4	80.4	79.4	78.8	100.0										
GCA_036965285.1	*Neobacillus drentensis*	< 77	77.9	79.6	79.0	80.1	77.7	79.1	78.5	80.1	85.8	78.2	85.5	84.0	79.8	78.2	78.6	82.5	79.5	83.9	78.7	80.0	100.0									
GCA_030915385.1	*Neobacillus sp. PS3-12*	< 77	< 77	< 77	< 77	< 77	< 77	< 77	< 77	79.2	< 77	< 77	77.9	< 77	< 77	< 77	< 77	< 77	< 77	< 77	< 77	< 77	< 77	100.0								
GCA_003999735.1	*Neobacillus mesonae*	< 77	78.1	78.6	78.8	78.5	78.2	< 77	< 77	< 77	77.9	78.9	77.8	78.2	< 77	< 77	79.3	78.1	78.4	78.1	< 77	78.6	78.0	< 77	100.0							
GCA_036496045.1	*Neobacillus sp.*	< 77	77.0	78.9	78.2	78.7	< 77	77.7	78.2	78.6	78.4	77.2	78.0	77.8	78.0	77.3	77.3	77.9	77.9	77.8	77.7	78.4	78.2	77.2	77.3	100.0						
GCA_023715415.1	*Neobacillus niacini*	< 77	< 77	78.2	78.4	78.4	77.8	84.2	< 77	78.4	78.7	< 77	78.6	78.3	89.9	78.7	< 77	78.4	78.3	78.3	79.3	78.5	78.6	< 77	< 77	77.6	100.0					
GCA_016908975.1	*Neobacillus cucumis*	< 77	78.4	78.6	79.1	78.9	78.0	78.5	78.5	79.2	78.8	78.3	78.9	79.4	78.4	< 77	78.5	78.9	79.6	79.7	78.6	79.5	79.2	78.8	< 77	77.6	78.3	100.0				
GCA_943193175.1	*Neobacillus muris*	< 77	81.9	77.8	78.0	77.9	77.8	< 77	< 77	< 77	< 77	78.2	< 77	< 77	< 77	< 77	78.3	< 77	78.1	< 77	< 77	78.2	< 77	< 77	77.9	77.2	< 77	< 77	100.0			
GCA_036219525.1	*Neobacillus thermocopriae*	< 77	77.8	78.1	77.9	77.8	95.3	77.7	77.8	77.8	77.6	78.1	77.6	77.8	77.6	< 77	78.3	77.9	78.1	77.6	77.9	78.3	77.7	< 77	78.1	77.0	77.6	77.7	77.7	100.0		
GCA_001636395.1	*Neobacillus novalis*	< 77	78.4	100.0	80.5	82.3	78.1	78.9	78.9	79.7	79.6	78.5	79.5	79.2	78.5	78.2	79.0	79.2	79.8	79.3	79.0	81.1	79.6	< 77	78.9	78.9	78.3	78.6	77.8	78.2	100.0	
GCA_001026695.1	*Neobacillus vireti*	< 77	78.3	90.4	80.3	82.1	78.2	78.3	78.5	79.0	79.6	78.6	79.4	79.3	78.4	77.9	79.0	79.2	79.6	79.3	78.6	81.0	79.4	< 77	78.6	78.8	78.4	78.9	78.0	78.2	90.4	100.0
GCA_038592905.1	*Neobacillus sp. FSL H8-0543*	< 77	< 77	78.5	78.6	78.4	77.4	79.9	< 77	79.3	78.5	< 77	78.5	78.5	79.6	86.1	< 77	78.4	78.5	78.6	79.3	78.3	78.7	< 77	< 77	77.8	79.1	< 77	< 77	77.5	79.0	78.6
GCA_030127045.1	*Neobacillus sp. 114*	< 77	78.4	78.6	78.5	78.6	78.4	< 77	78.6	< 77	78.4	93.5	78.3	78.4	78.3	< 77	99.0	78.1	78.6	78.3	78.9	78.8	78.6	< 77	79.5	77.3	< 77	78.6	78.2	78.3	79.1	78.9
GCA_036962465.1	*Neobacillus vireti*	< 77	78.4	78.8	79.2	79.1	77.9	78.4	78.0	78.9	79.0	78.4	79.3	79.3	78.3	< 77	78.3	79.3	79.7	79.5	78.6	79.7	79.2	< 77	78.2	77.6	78.2	82.2	77.9	78.0	78.9	79.2
GCA_030123445.1	*Neobacillus novalis*	< 77	78.2	100.0	80.5	82.3	78.1	79.1	78.9	79.7	79.6	78.5	79.5	79.2	78.5	78.2	78.9	79.2	79.8	79.2	78.9	81.1	79.6	< 77	79.0	78.9	78.3	78.7	77.9	78.1	100.0	90.4
GCA_021560175.1	*Neobacillus drentensis*	< 77	78.3	78.8	79.7	79.0	77.8	79.2	78.4	79.4	79.1	78.6	79.5	79.4	78.5	< 77	78.9	79.1	79.8	79.5	79.3	79.7	79.3	< 77	78.9	77.7	78.3	82.2	77.9	78.0	79.3	79.2
GCA_002335815.1	*Neobacillus soli*	< 77	< 77	82.8	80.1	80.8	77.8	78.8	78.8	79.5	80.1	78.4	79.9	79.8	78.5	78.1	78.7	79.7	79.6	79.8	78.9	80.4	80.4	< 77	78.8	78.6	78.4	78.6	77.7	77.9	83.1	81.8
GCA_000612625.1	*Neobacillus jeddahensis*	< 77	78.4	79.6	80.0	79.5	78.0	78.4	78.2	78.7	79.2	78.2	79.3	79.2	78.6	< 77	78.4	79.3	99.9	79.3	78.3	80.3	79.4	< 77	78.1	77.9	78.3	79.4	78.1	77.9	79.6	79.5
GCA_030915485.1	*Neobacillus sp. PS3-40*	< 77	< 77	< 77	< 77	77.8	< 77	78.4	77.8	78.6	< 77	< 77	< 77	< 77	< 77	< 77	< 77	< 77	< 77	< 77	< 77	77.9	78.0	78.5	< 77	77.5	< 77	< 77	< 77	< 77	78.3	< 77
GCA_031988165.1	*Neobacillus sp.*	< 77	< 77	82.2	79.9	96.2	77.9	78.5	79.2	79.1	79.5	78.2	79.5	79.4	78.4	78.2	78.4	79.3	79.6	79.3	78.5	82.6	80.0	< 77	78.3	78.7	78.2	78.7	77.9	77.8	82.2	81.9
GCA_030817695.1	*Neobacillus niacini*	< 77	< 77	78.2	79.0	78.5	77.8	84.4	< 77	79.4	78.6	< 77	78.6	79.0	84.7	79.0	< 77	78.3	78.7	79.0	80.3	78.7	79.2	< 77	< 77	77.5	83.9	78.6	< 77	77.5	78.7	78.4
GCA_007828895.1	*Neobacillus bataviensis*	< 77	78.2	78.9	79.3	79.1	78.0	78.9	78.3	79.8	79.3	78.3	79.4	80.2	78.9	< 77	78.6	79.0	79.7	80.4	78.9	79.7	80.2	< 77	78.3	77.9	78.4	83.1	78.0	77.7	79.1	79.0
GCA_030123405.1	*Neobacillus sp. WH10*	< 77	78.4	81.0	82.2	82.5	78.2	79.2	79.6	79.8	79.5	78.4	79.5	79.5	78.7	78.1	79.0	79.4	80.3	79.4	79.3	94.8	79.8	< 77	79.1	78.4	78.5	79.5	78.2	78.1	81.2	80.8
GCA_029240335.1	*Neobacillus sp.*	< 77	< 77	78.2	77.8	78.1	< 77	84.1	< 77	78.3	78.7	77.5	78.6	78.8	84.8	79.3	77.7	78.3	78.0	78.7	79.3	78.2	78.3	< 77	< 77	77.6	84.0	78.3	< 77	< 77	78.2	78.1
GCA_030915425.1	*Neobacillus sp. OS1-32*	< 77	< 77	78.0	78.5	78.2	77.9	< 77	< 77	< 77	< 77	78.0	< 77	< 77	< 77	< 77	78.3	< 77	< 77	< 77	< 77	78.2	< 77	< 77	79.7	77.1	< 77	< 77	77.7	77.8	78.5	78.0
GCA_000768265.1	*Neobacillus niacini*	< 77	< 77	78.5	78.7	78.6	< 77	83.1	< 77	78.9	78.9	78.4	79.2	78.9	83.6	79.0	78.4	78.7	78.8	78.8	79.7	78.7	79.1	< 77	< 77	< 77	82.8	78.6	< 77	< 77	78.4	78.7
GCA_902375305.1	*Neobacillus jeddahensis*	< 77	78.4	79.6	80.0	79.5	78.0	78.4	78.2	78.7	79.2	78.2	79.3	79.2	78.6	< 77	78.4	79.3	99.9	79.3	78.3	80.3	79.4	< 77	78.1	77.9	78.3	79.4	78.1	77.9	79.6	79.5
GCA_019749295.1	*Neobacillus niacini*	< 77	< 77	77.9	78.2	78.2	77.6	84.5	< 77	78.6	78.1	< 77	78.3	78.2	85.0	78.9	< 77	78.3	78.5	78.3	79.3	78.5	78.6	< 77	< 77	77.7	84.2	78.2	< 77	77.6	78.1	78.4
GCA_036965565.1	*Neobacillus drentensis*	< 77	< 77	79.4	79.4	79.7	78.0	78.8	78.4	78.9	79.3	78.3	79.3	79.4	78.7	< 77	78.5	79.3	79.7	79.3	78.7	79.9	79.5	< 77	78.2	78.0	78.8	78.9	< 77	78.1	79.4	79.4
GCA_031457145.1	*Neobacillus niacini*	< 77	< 77	78.7	78.5	78.8	77.6	83.0	< 77	79.0	79.1	< 77	78.5	78.7	83.9	79.1	78.3	78.5	78.3	78.8	79.3	78.7	79.0	< 77	< 77	77.7	83.1	78.2	< 77	77.5	78.8	78.5
GCA_036962325.1	*Neobacillus drentensis*	< 77	< 77	79.2	78.9	79.3	77.8	78.9	78.1	78.9	82.0	78.2	82.6	83.7	78.6	78.2	78.1	99.7	79.2	83.7	78.5	79.3	82.6	< 77	78.1	78.0	78.3	78.9	< 77	77.8	79.2	79.3

Supplemental File S1. ANI matrix of all genomes of *Neobacillus* :

GenBank Accession #	Scientific Name	*Neobacillus sp. FSL H8-0543*	*Neobacillus sp. 114*	*Neobacillus vireti*	*Neobacillus novalis*	*Neobacillus drentensis*	*Neobacillus soli*	*Neobacillus jeddahensis*	*Neobacillus sp. PS3-40*	*Neobacillus sp.*	*Neobacillus niacini*	*Neobacillus bataviensis*	*Neobacillus sp. WH10*	*Neobacillus sp.*	*Neobacillus sp. OS1-32*	*Neobacillus niacini*	*Neobacillus jeddahensis*	*Neobacillus niacini*	*Neobacillus drentensis*	*Neobacillus niacini*	*Neobacillus drentensis*
**GCA_030732025.1**	***Neobacillus driksii 179-C4-2-HS(T)***																				
**JBDZYE000000000**	***Neobacillus driksii 179-J1A1-HS***																				
**GCA_013409995.1**	***Neobacillus driksii AT2.8***																				
**GCA_030817595.1**	***Neobacillus driksii V4I25***																				
**GCA_001591505.1**	***Neobacillus niacini NBRC 15566***																				
GCA_011393025.1	*Neobacillus terrae*																				
GCA_023715865.1	*Neobacillus mesonae*																				
GCA_035809615.1	*Neobacillus niacini*																				
GCA_030915465.1	*Neobacillus sp. PS3-34*																				
GCA_030123365.1	*Neobacillus sp. SuZ13*																				
GCA_036223585.1	*Neobacillus cucumis*																				
GCA_036962345.1	*Neobacillus vireti*																				
GCA_030915405.1	*Neobacillus sp. OS1-33*																				
GCA_023715505.1	*Neobacillus cucumis*																				
GCA_036962725.1	*Neobacillus drentensis*																				
GCA_036962365.1	*Neobacillus drentensis*																				
GCA_036965785.1	*Neobacillus vireti*																				
GCA_002860255.1	*Neobacillus cucumis*																				
GCA_036219545.1	*Neobacillus thermocopriae*																				
GCA_000307875.1	*Neobacillus bataviensis LMG 21833*																				
GCA_036961715.1	*Neobacillus vireti*																				
GCA_036962765.1	*Neobacillus drentensis*																				
GCA_031324245.1	*Neobacillus cucumis*																				
GCA_003362805.1	*Neobacillus piezotolerans*																				
GCA_013248975.1	*Neobacillus endophyticus*																				
GCA_030123505.1	*Neobacillus sp. YX16*																				
GCA_036962905.1	*Neobacillus drentensis*																				
GCA_030123065.1	*Neobacillus sp. DY30*																				
GCA_031457825.1	*Neobacillus drentensis*																				
GCA_032667185.1	*Neobacillus sp. YIM B06451*																				
GCA_016765675.1	*Neobacillus paridis*																				
GCA_036962645.1	*Neobacillus niacini*																				
GCA_023715785.1	*Neobacillus niacini*																				
GCA_000508325.2	*Neobacillus vireti LMG 21834*																				
GCA_030915525.1	*Neobacillus sp. PS2-9*																				
GCA_016107705.1	*Neobacillus cucumis*																				
GCA_036961885.1	*Neobacillus niacini*																				
GCA_001591485.1	*Neobacillus fumarioli NBRC 102428*																				
GCA_000759675.1	*Neobacillus niacini*																				
GCA_036963385.1	*Neobacillus niacini*																				
GCA_017353195.1	*Neobacillus sp. MM2021_6*																				
GCA_023715155.1	*Neobacillus mesonae*																				
GCA_001591665.1	*Neobacillus soli NBRC 102451*																				
GCA_010975035.1	*Neobacillus thermocopriae*																				
GCA_019969725.1	*Neobacillus bataviensis*																				
GCA_036223565.1	*Neobacillus mesonae*																				
GCA_030813055.1	*Neobacillus ginsengisoli*																				
GCA_014656545.1	*Neobacillus kokaensis*																				
GCA_036965485.1	*Neobacillus drentensis*																				
GCA_010614825.1	*Neobacillus sedimentimangrovi*																				
GCA_036962665.1	*Neobacillus niacini*																				
GCA_030316255.1	*Neobacillus mesonae*																				
GCA_036961795.1	*Neobacillus vireti*																				
GCA_030123465.1	*Neobacillus cucumis*																				
GCA_001636315.1	*Neobacillus mesonae*																				
GCA_036965145.1	*Neobacillus drentensis*																				
GCA_004116995.1	*Neobacillus thermocopriae*																				
GCA_001636415.1	*Neobacillus drentensis*																				

GenBank Accession #	Scientific Name	*Neobacillus sp. FSL H8-0543*	*Neobacillus sp. 114*	*Neobacillus vireti*	*Neobacillus novalis*	*Neobacillus drentensis*	*Neobacillus soli*	*Neobacillus jeddahensis*	*Neobacillus sp. PS3-40*	*Neobacillus sp.*	*Neobacillus niacini*	*Neobacillus bataviensis*	*Neobacillus sp. WH10*	*Neobacillus sp.*	*Neobacillus sp. OS1-32*	*Neobacillus niacini*	*Neobacillus jeddahensis*	*Neobacillus niacini*	*Neobacillus drentensis*	*Neobacillus niacini*	*Neobacillus drentensis*
GCA_036965425.1	*Neobacillus drentensis*																				
GCA_023714155.1	*Neobacillus sp. MER 74*																				
GCA_036965445.1	*Neobacillus niacini*																				
GCA_031456445.1	*Neobacillus niacini*																				
GCA_003515685.1	*Neobacillus notoginsengisoli*																				
GCA_000612665.1	*Neobacillus dielmonensis*																				
GCA_001591805.1	*Neobacillus novalis NBRC 102450*																				
GCA_030915505.1	*Neobacillus sp. OS1-2*																				
GCA_937468385.1	*Neobacillus rhizosphaerae*																				
GCA_021109295.1	*Neobacillus sedimentimangrovi*																				
GCA_030348765.1	*Neobacillus sp. CF12*																				
GCA_001048695.1	*Neobacillus massiliamazoniensis*																				
GCA_023702235.1	*Neobacillus pocheonensis*																				
GCA_001591445.1	*Neobacillus drentensis NBRC 102427*																				
GCA_018343545.2	*Neobacillus citreus*																				
GCA_036963125.1	*Neobacillus drentensis*																				
GCA_036961895.1	*Neobacillus drentensis*																				
GCA_036963445.1	*Neobacillus drentensis*																				
GCA_029256785.1	*Neobacillus sp.*																				
GCA_018343535.1	*Neobacillus rhizophilus*																				
GCA_036962125.1	*Neobacillus drentensis*																				
GCA_000820865.2	*Neobacillus jeddahensis*																				
GCA_036966985.1	*Neobacillus drentensis*																				
GCA_946151075.1	*Neobacillus sp. Marseille-Q6967*																				
GCA_036965245.1	*Neobacillus drentensis*																				
GCA_036965285.1	*Neobacillus drentensis*																				
GCA_030915385.1	*Neobacillus sp. PS3-12*																				
GCA_003999735.1	*Neobacillus mesonae*																				
GCA_036496045.1	*Neobacillus sp.*																				
GCA_023715415.1	*Neobacillus niacini*																				
GCA_016908975.1	*Neobacillus cucumis*																				
GCA_943193175.1	*Neobacillus muris*																				
GCA_036219525.1	*Neobacillus thermocopriae*																				
GCA_001636395.1	*Neobacillus novalis*																				
GCA_001026695.1	*Neobacillus vireti*																				
GCA_038592905.1	*Neobacillus sp. FSL H8-0543*	100.0																			
GCA_030127045.1	*Neobacillus sp. 114*	< 77	100.0																		
GCA_036962465.1	*Neobacillus vireti*	< 77	78.3	100.0																	
GCA_030123445.1	*Neobacillus novalis*	79.1	79.2	78.9	100.0																
GCA_021560175.1	*Neobacillus drentensis*	78.3	79.1	91.8	79.4	100.0															
GCA_002335815.1	*Neobacillus soli*	79.0	78.8	78.8	83.1	79.2	100.0														
GCA_000612625.1	*Neobacillus jeddahensis*	78.4	78.4	79.7	79.5	79.7	79.4	100.0													
GCA_030915485.1	*Neobacillus sp. PS3-40*	< 77	< 77	< 77	< 77	< 77	78.4	< 77	100.0												
GCA_031988165.1	*Neobacillus sp.*	78.3	78.3	79.1	82.2	79.0	80.7	79.6	77.9	100.0											
GCA_030817695.1	*Neobacillus niacini*	79.7	< 77	78.5	79.0	79.2	78.9	78.6	< 77	78.5	100.0										
GCA_007828895.1	*Neobacillus bataviensis*	78.0	78.6	85.6	79.1	85.9	78.9	79.7	< 77	79.2	79.0	100.0									
GCA_030123405.1	*Neobacillus sp. WH10*	78.8	79.3	79.5	81.2	80.1	80.5	80.3	78.7	82.4	79.1	79.7	100.0								
GCA_029240335.1	*Neobacillus sp.*	79.1	77.7	78.1	78.2	78.2	78.0	78.1	< 77	78.0	88.4	78.4	78.2	100.0							
GCA_030915425.1	*Neobacillus sp. OS1-32*	< 77	78.5	< 77	78.6	< 77	78.3	< 77	< 77	77.9	< 77	< 77	78.4	< 77	100.0						
GCA_000768265.1	*Neobacillus niacini*	79.4	78.8	78.5	78.6	78.5	78.8	< 77	< 77	78.7	86.5	78.8	78.9	86.1	< 77	100.0					
GCA_902375305.1	*Neobacillus jeddahensis*	78.4	78.4	79.7	79.5	79.7	79.4	100.0	< 77	79.6	78.6	79.7	80.3	78.1	< 77	< 77	100.0				
GCA_019749295.1	*Neobacillus niacini*	79.1	< 77	78.3	78.1	78.3	78.3	78.4	< 77	78.2	83.7	78.4	78.4	83.6	< 77	82.9	78.4	100.0			
GCA_036965565.1	*Neobacillus drentensis*	78.5	78.5	79.3	79.5	79.3	79.3	79.7	< 77	79.7	78.6	79.4	79.9	78.3	< 77	78.7	79.7	78.5	100.0		
GCA_031457145.1	*Neobacillus niacini*	80.0	78.5	78.1	78.8	78.3	79.1	78.3	< 77	78.6	83.3	78.5	78.7	83.2	< 77	82.8	78.3	82.6	78.8	100.0	
GCA_036962325.1	*Neobacillus drentensis*	78.4	78.1	79.2	79.3	79.1	79.7	79.2	< 77	79.3	78.4	79.1	79.4	78.4	< 77	78.7	79.2	78.3	79.3	78.5	78.5

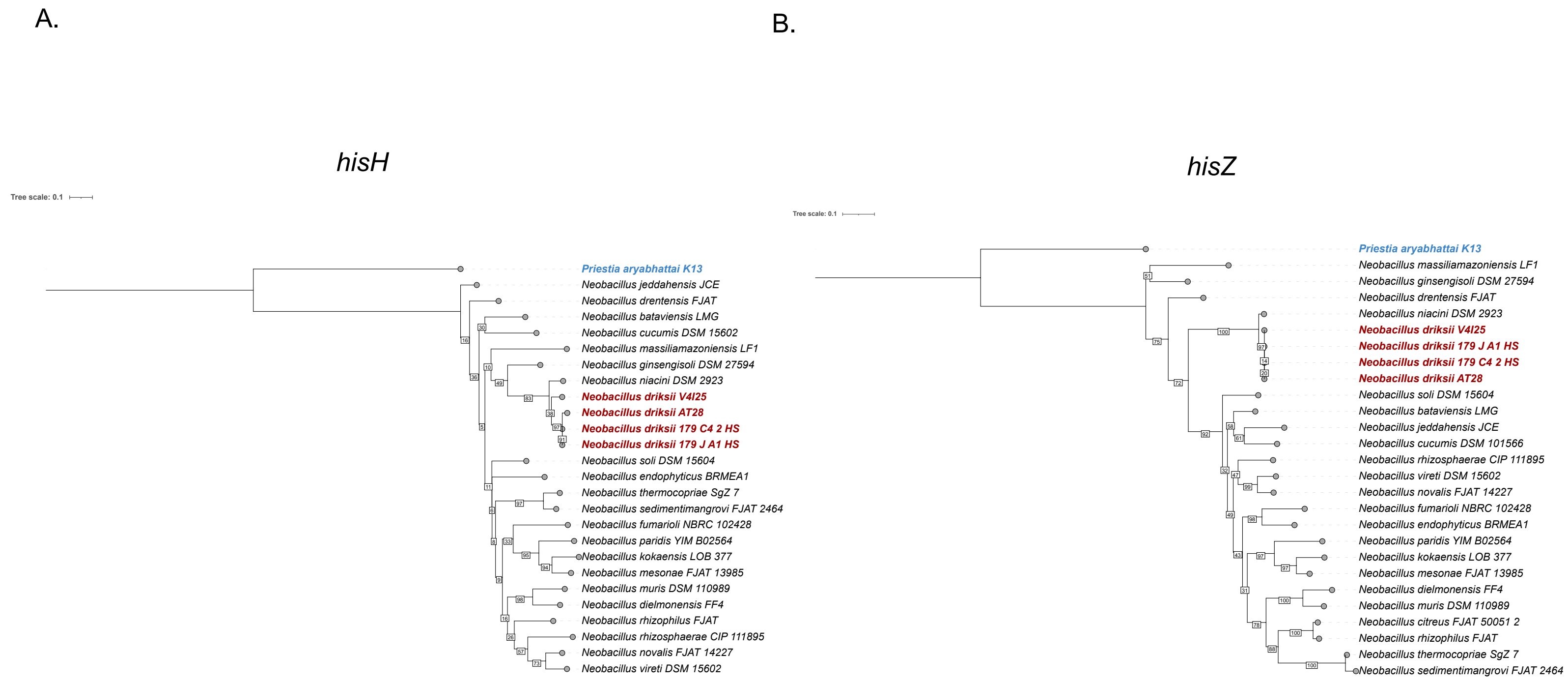

Supplemental File S2. Phylogenetic inference based on amino acid multisequence alignment of the imidazole glycerol phosphate synthase subunit [*hisH*] (A) and ATP phosphoribosyltransferase regulatory subunit [*his*Z] (B) of all reference genomes of the genus *Neobacillus* including the three genomes of *N. driksii* highlighted in red text. *P. polymyxa* ATCC 842 was used as an outgroup. Bootstrap support values of >50% are presented on nodes along with branch distances rounded to three decimal spaces.

Supplemental File S3. Differential phenotypic characteristics of *Neobacillus driksii* 179-C4-2-HS^T and other related species of *Neobacillus*.

Taxa: 1, *Neobacillus driksii* 179-C4-2-HS^T; 2, *N. niacini* (type strain DSM 2923^T); 3, *N. drentensis* (type strain IDA1967^T); 4, *N. bataviensis* (type strain IDA1115^T); 5, *N. soli* (type strain IDA0086^T).

-, negative; +, positive; w, weak; V, variable; ND, not determined; ST, subterminal.

**Characteristic**	**1**	**2**	**3**	**4**	**5**
Oxidase	+	ND	ND	ND	ND
Catalase	W	ND	ND	ND	ND
Gram stain	+	V	+/V	+/V	+/V
Cell ends	Rounded	Rounded	Tapered	Slightly tapered	Rounded
Spore shape	Ellipsoidal	Ellipsoidal	Ellipsoidal/Circular	Ellipsoidal (Circular)	Ellipsoidal
Spore position	ST	Central/ST	ST	Central/ST	ST
Sporangia swollen	W	-/W	+	W	W
Growth at 50 °C	-	-	+	+	-
Growth temperature range (°C)	4-45	10-40	max 50/55	max 50/55	max 40/45
NaCl tolerance (%)	5	ND	ND	ND	ND
pH range	6 to 9.5	7-8	5.5/6-9.5/10	4/6-9.5/10	4/5-9/9.5
Anaerobic growth	+	ND	+	+	+
**Hydrolysis of:**					
Casein	-	-	-	-	+
Gelatin	-	+	-	+	+
Storage inclusions	ND	ND	+	-	-
ONPG	+	ND	+	+	-
Urease	-	(-)	-	-	-
Nitrate reduction to N2	+	(+)	V	+	+
**Acid production from:**					
Amygdalin	-	+	V	V	-
D-Cellobiose	+	V	-	+	-

L-Fucose	-	-	-	V	-
B-Gentiobiose	+	V	-	+	-
Glycerol	-	-	-	W	-
Glycogen	-	(+)	-	-	+
Lactose	+	(+)*	+	+	-
D-Mannitol	-	(-)	-	+	-
D-Melibiose	+	+	+	V	-
D-Melezitose	-	V	V	+	-
Methyl a-D-glucoside	-	V	V	V	-
Raffinose	-	(+)*	V	+	-
Ribose	-	V	V	W	W
Salicin	+	V	+	W	-
Starch	+	ND	V	V	+
Sucrose	+	+	V	V	V
D-Turanose	+	(-)*	V	+	-
G + C content (mol%)	38.3 to 38.4†	37-39	39.3-39.4	39.6-40.1	40.1-40.4

Data in 2 were from Nagel and Andreesen (1991); Data in 3, 4 and 5 were from Heyrman et al. (2004)

†, based on the genome sequence data; *, type strain was the only strain that gave a different result

References

Nagel M, Andreesen JR. (1991) *Bacillus niacini* sp. nov., a nicotinate-metabolizing mesophile isolated from soil. Int J Syst Bacteriol 41:134-139.

Heyrman J, Vanparys B, Logan NA, Balcaen A, Rodriguez-Diaz M, Felske A, De Vos P. 2004. *Bacillus novalis* sp. nov., *Bacillus vireti* sp. nov., *Bacillus soli* sp. nov., *Bacillus bataviensis* sp. nov. and *Bacillus drentensis* sp. nov., from the Drentse A grasslands. Int J Syst Evol Microbiol 54:47-57.

BioLog GNIII and Vitek data for *Neobacillus driksii* 179-C4-2-HS^T

- In BioLog GNIII MicroPlate, positive for dextrin, D-maltose, D-trehalose, D-cellobiose, gentiobiose, sucrose, d-turanose, pH6, raffinose, α-D-lactose, D-melibiose, β-methyl-D-glucoside, D-salicin, N-acetyl-D-glucosamine, N-acetyl-β-D-mannosamine, , α-D-glucose, D-mannose, D-fructose, D-galactose, 3-methyl glucose, L-rhamnose, inosine, 1% sodium lactate, fusidic acid, D-serine, D-glucose-6-phosphate, D-aspartic

acid, D-serine, L-alanine, L-arginine, L-aspartic acid, L-glutamic acid, L-serine, pectin, D-galacturonic acid, L-galactonic acid lactone, D-gluconic acid, D-glucuronic acid, glucuronamide, quinic acid, D-saccharic acid, tetrazolium violet, tetrazolium blue, L-lactic acid, D-malic acid, L-malic acid, bromo-succinic acid, nalidixic acid, lithium chloride, potassium tellurite, tween 40, β-hydroxy-D,L-butyric acid, acetoacetic acid, propionic acid, acetic acid, aztreonam, sodium butyrate; growth in the presence of 1% and 4% NaCl, troleandomycin, minocycline and lincomycin. Negative for stachyose, N-acetyl-D-galactosamine, N-acetyl neuraminic acid, D-fucose, L-fucose, D-sorbitol, D-mannitol, D-arabitol, myo-inositol, glycerol, D-fructose-6-phosphate, gelatin, glycyl-L-proline, L-histidine, L-pyroglutamic acid, guanidine HCl, mucic acid, vancomycin, p-hydroxy-phenylacetic acid, methyl pyruvate, D-lactic acid methyl ester, citric acid, α-keto-glutaric acid, γ-amino-butyric acid, α-hydroxy-butyric acid, α-keto-butyric acid, formic acid; sodium bromate; growth in the presence of pH5, 8% NaCl, rifamycin SV and niaproof 4.

- In Vitek GP2 card, positive for L-proline arylamidase, β-glucuronidase, alanine arylamidase and D-trehalose. Negative for D-amygdalin, phosphatidylinositol phospholipase C, D-xylose, arginine dihydrolase 1, β -galactosidase, α-glucosidase, Ala-Phe-Pro arylamidase, cyclodextrin, L-aspartate arylamidase, β galactopiranosidase, α-mannosidase, phosphatase, leucine arylamidase, α-galactosidase, L-pyrrolydonyl-arylamidase, β-glucuronidase, tyrosine arylamidase, D-sorbitol, urease, polymixin B resistance, D-galactose, D-ribose, L-lactate alkalization, lactose, N-acetyl-D-glucosamine, D-maltose, bacitracin resistance, novobiocin resistance, growth in 6.5% NaCl, D-mannitol, D-mannose, methyl-B-D-glucopyranoside, pullulan, D-raffinose, O/129 resistance (comp. vibrio), salicin, saccharose/sucrose, arginine dihydrolase 2 and optochin resistance

Supplemental File S4. Cellular fatty acid methyl ester profile of *Neobacillus driksii* 179-C4-2-HS[T] and other related species of *Neobacillus*.

Taxa: 1, *Neobacillus driksii* 179-C4-2-HS[T]; 2, *N. niacini* DSM 2923[T]; 3, *N. drentensis* (10 isolates); 4, *N. bataviensis* (11 isolates); 5, *N. soli* (5 isolates); 6, *N. cucumis* CCM 8651[T]; 7, *N. pocheonensis* Gsoil 420[T]. -, not detected; tr, trace.

**Fatty acid**	**1**	**2[a]**	**3[b]**	**4[b]**	**5[b]**	**6[a]**	**7[c]**
$C_{14:0}$	1.6	1.9	1.4	1.5	tr	2.6	1.8
iso-$C_{14:0}$	3.9	5.1	8.7	6.9	3.3	4.3	19.9
iso-$C_{15:0}$	43.6	33.7	32.2	36.9	42.9	38.6	24.5
anteiso-$C_{15:0}$	32.7	40.6	21.8	20.5	33.5	33.2	33.9
$C_{16:0}$	3.4	9.4	3.4	7.7	1.6	13.2	2.4
iso-$C_{16:0}$	2.2	-	2.1	2.4	1.4	-	7.5
$C_{16:1}$ *ω*11*c*	2.6	5.2	13.2	11.3	3.2	8.0	1.9
$C_{16:1}$ *ω*7*c* alcohol	1.6	-	3.1	2.3	2.4	-	5.0
iso-$C_{17:0}$	1.6	1.8	2.6	1.4	2.6	-	0.8
anteiso-$C_{17:0}$	2.0	-	1.1	1.1	1.5	-	1.5
iso-$C_{17:1}$ *ω*10*c*	1.8	-	4.5	1.7	4.1	-	-
$C_{18:0}$	0.7	-	1.3	tr	tr	-	-
$C_{18:1}$ *ω*9*c*	0.5	2.3	1.8	tr	tr	-	-

[a]Data in 2 and 6 were from Xue et al. (2021).
[b]Data in 3, 4 and 5 were mean values taken from Heyrman et al. (2004)
[c]Data in 7 were taken from Ten et al. (2007).

Supplementary References:

1. Xue L, Tang L, Zhao J, Fang Z, Liu H, Qiao J, Zhang G. 2021. *Bacillus salipaludis* sp. nov., isolated from saline-alkaline soil. Arch Microbiol 203:2211-2217.
2. Heyrman J, Vanparys B, Logan NA, Balcaen A, Rodriguez-Diaz M, Felske A, De Vos P. 2004. *Bacillus novalis* sp. nov., *Bacillus vireti* sp. nov., *Bacillus soli* sp. nov., *Bacillus bataviensis* sp. nov. and *Bacillus drentensis* sp. nov., from the Drentse A grasslands. Int J Syst Evol Microbiol 54:47-57.
3. Ten LN, Baek SH, Im WT, Larina LL, Lee JS, Oh HM, Lee ST. 2007. *Bacillus pocheonensis* sp. nov., a moderately halotolerant, aerobic bacterium isolated from soil of a ginseng field. Int J Syst Evol Microbiol 57:2532-2537.

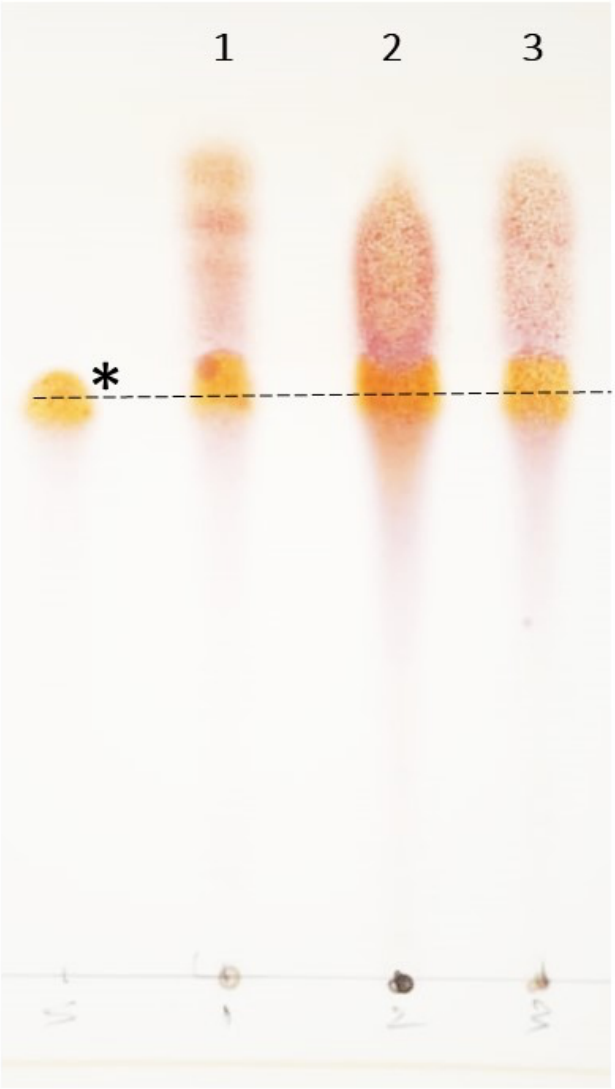

Supplemental File S5. Thin layer chromatographic plate showing the identification of meso-diaminopimelic acid in bacterial isolates of ISS. 1, *Neobacillus driksii* sp. nov. 179-C4-2-HS^{T}; 2, *Tigheibacillus jepli* 179-BFC-A-HS^{T}; 3, *Paenibacillus vandeheii* F6_3S_P_1C^{T}. *, meso 2,6-diminopimelic acid standard (meso-DAP).

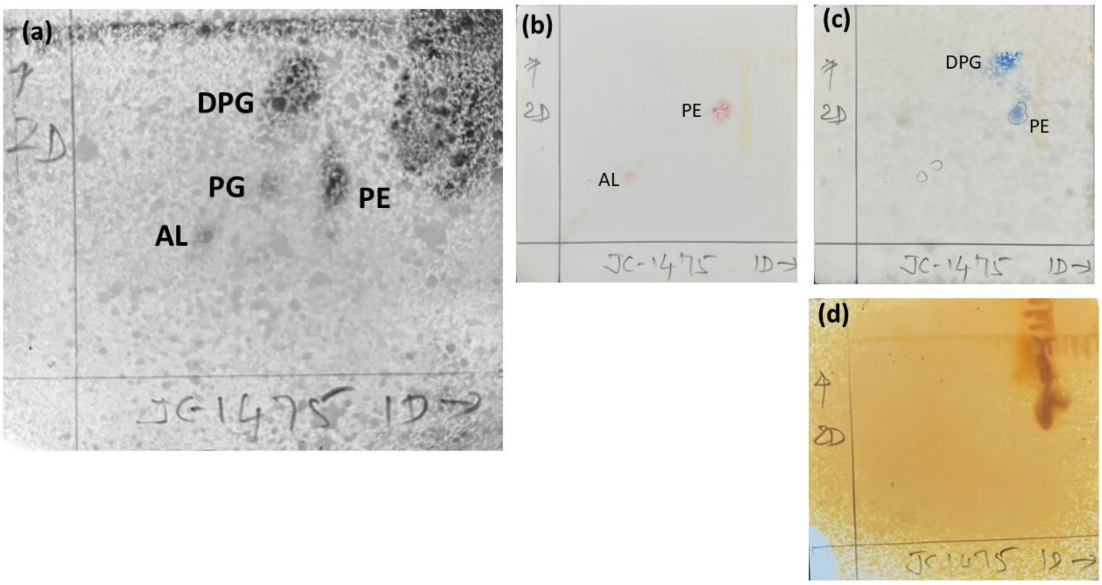

Supplemental File S6. Polar lipid profile of *Neobacillus driksii* 179-C4-2-HS^{T} as determined by two-dimensional thin layer chromatography (2D TLC). Lipids were stained with 10 % (w/v) ethanolic phosphomolybdic acid (a), 0.2 % (w/v) solution of ninhydrin in butanol (b), Dittmer and Lester's Zinzadze reagent (c) and α-naphthol spray reagent (d) to visualize total, amino, phsopho and glycolipids, respectively. DPG, diphosphatidylglycerol; PG, phosphatidylglycerol; PE, phosphatidylethanolamine; AL, Unidentified aminolipid.

Supplemental File S7. Major BGCs predicted in the genomes of four *N. driksii* strains and *N. niacini* type strain

Type of secondary metabolite	Cluster name	Class	*N. niacini* NBRC 15566 GCF_001591505.1	*N. driksii* strains 179_C42_HS JAROBZ	179-J1A1-HS JBDZYE	AT2.8 GCF_013409995.1	V4I35 GCF_030817595.1
lassopeptide	paeninodin	RiPP	Not present	80%*	80%	80%	80%
NI-siderophore	schizokinen	Other	Not present	60%	60%	60%	60%
betalactone	fengycin	NRP	40%	40%	40%	40%	40%
T3PKS			Unknown	Unknown	Unknown	Unknown	Unknown
LAP			Unknown	Unknown	Unknown	Unknown	Unknown
terpene			Unknown	Unknown	Unknown	Unknown	Unknown

*80% similarities are with *P. dendritiformis* C454 (8).

Supplemental FIle S8. DeepFRI-based COG characteristics of *N. driksii* and type strain of *N. niacini*

GO_term	GO_term_name	COG_LETTER	*N. niacini*	*N. driksii* strains			
			DSM 2923T	179-C4-2-HS	.79-J 1A1 H:	AT2.8	V4I25
GO:0031082	BLOC complex	O	1	1	1	1	1
GO:0044237	cellular metabolic process	C	1	1	1	1	1
GO:0031406	carboxylic acid binding	R	1	1	1	1	1
GO:0008092	cytoskeletal protein binding	Z	1	1	1	1	1
GO:0016695	oxidoreductase activity, acting on hydrogen as donor	C	1	1	1	1	1
GO:0019693	ribose phosphate metabolic process	G	1	1	1	1	1
GO:0019321	pentose metabolic process	H, G	1	1	1	1	1
GO:0016709	oxidoreductase activity, acting on paired donors, with incorporation or reduction of molecular oxygen, NAD(P)H as one donor, and incorporation of one atom of oxygen	H	1	1	1	1	1
GO:0043831	thiosulfate dehydrogenase (quinone) activity	C	1	1	1	1	1
GO:0080090	regulation of primary metabolic process	T, C	1	1	1	1	1
GO:0016705	oxidoreductase activity, acting on paired donors, with incorporation or reduction of molecular oxygen	C	1	1	1	1	1
GO:0019439	aromatic compound catabolic process	C	1	1	1	1	1
GO:0018786	haloalkane dehalogenase activity	C	1	1	1	1	1
GO:0051172	negative regulation of nitrogen compound metabolic process	C	1	1	1	1	1
GO:0016810	hydrolase activity, acting on carbon-nitrogen (but not peptide) bonds	C	1	1	1	1	1
GO:0033559	unsaturated fatty acid metabolic process	I	1	1	1	1	1
GO:0061695	transferase complex, transferring phosphorus-containing groups	O	1	1	1	1	1
GO:0022803	passive transmembrane transporter activity	U	1	1	1	1	1
GO:0044042	glucan metabolic process	G	1	1	1	1	1
GO:0045892	negative regulation of DNA-templated transcription	K	1	1	1	1	1
GO:0044270	cellular nitrogen compound catabolic process	C	1	1	1	1	1
GO:0046483	heterocycle metabolic process	C	1	1	1	1	1
GO:0006139	nucleobase-containing compound metabolic process	C	1	1	1	1	1
GO:0016210	naringenin-chalcone synthase activity	I	1	1	1	1	1
GO:0016740	transferase activity	R	1	1	1	1	1
GO:0008874	gluconate 5-dehydrogenase activity	C	1	1	1	1	1
GO:0008238	exopeptidase activity	O	1	1	1	1	1
GO:0015930	glutamate synthase activity	E	1	1	1	1	1
GO:0008283	cell population proliferation	D	1	1	1	1	1
GO:0046914	transition metal ion binding	P	1	1	1	1	1
GO:1904949	ATPase complex	C	1	1	1	1	1
GO:0015157	oligosaccharide transmembrane transporter activity	G	1	1	1	1	1
GO:0016661	oxidoreductase activity, acting on other nitrogenous compounds as donors	C	1	1	1	1	1
GO:0050020	L-arabinonate dehydratase activity	H	1	1	1	1	1
GO:0050118	N-acetyldiaminopimelate deacetylase activity	C	1	1	1	1	1
GO:0015850	organic hydroxy compound transport	R	1	1	1	1	1
GO:0048869	cellular developmental process	D	1	1	1	1	1
GO:0006703	estrogen biosynthetic process	I, C	1	1	1	1	1
GO:0031554	regulation of termination of DNA-templated transcription	K, O	1	1	1	1	1
GO:0045181	glutamate synthase activity, NAD(P)H as acceptor	E	1	1	1	1	1
GO:0099503	secretory vesicle	U	1	1	1	1	1
GO:0033036	macromolecule localization	R	1	1	1	1	1
GO:0019566	arabinose metabolic process	H, G	1	1	1	1	1
GO:0009526	plastid envelope	M	1	1	1	1	1
GO:0000779	condensed chromosome, centromeric region	B	1	1	1	1	1
GO:0046527	glucosyltransferase activity	G	1	1	1	1	1
GO:0003712	transcription coregulator activity	K	1	1	1	1	1
GO:0033655	host cell cytoplasm part	R	1	1	1	1	1
GO:0004081	bis(5'-nucleosyl)-tetraphosphatase (asymmetrical) activity	C	1	1	1	1	1
GO:0019572	L-arabinose catabolic process	H, G	1	1	1	1	1
GO:0050440	2-methylcitrate synthase activity	C	1	1	1	1	1
GO:0098796	membrane protein complex	M, O	1	1	1	1	1
GO:0031982	vesicle	M	1	1	1	1	1
GO:0060255	regulation of macromolecule metabolic process	T, C	1	1	1	1	1
GO:0046857	oxidoreductase activity, acting on other nitrogenous compounds as donors, with NAD or NADP as acceptor	C	1	1	1	1	1
GO:0050138	nicotinate dehydrogenase activity	C	1	1	1	1	1
GO:0016558	protein import into peroxisome matrix	O	1	1	1	1	1
GO:0004029	aldehyde dehydrogenase (NAD+) activity	C	1	1	1	1	1
GO:0030154	cell differentiation	D	1	1	1	1	1
GO:0052873	FMN reductase (NADPH) activity	C	1	1	1	1	1
GO:0022804	active transmembrane transporter activity	U	1	1	1	1	1
GO:0016790	thiolester hydrolase activity	H	1	1	1	1	1
GO:0044249	cellular biosynthetic process	C	1	1	1	1	1
GO:0015079	potassium ion transmembrane transporter activity	P	1	1	1	1	1
GO:0047789	creatininase activity	C	1	1	1	1	1
GO:0018193	peptidyl-amino acid modification	O	1	1	1	1	1
GO:0031428	box C/D RNP complex	O	1	1	1	1	1
GO:0000228	nuclear chromosome	B	1	1	1	1	1
GO:0019752	carboxylic acid metabolic process	H	1	1	1	1	1
GO:0019904	protein domain specific binding	O	1	1	1	1	1
GO:0008324	monoatomic cation transmembrane transporter activity	P	1	1	1	1	1

GO_term	GO_term_name	COG_LETTER	*N. niacini*	*N. driksii* strains			
			DSM 2923T	179-C4-2-HS	.79-J 1A1 H	AT2.8	V4I25
GO:0019899	enzyme binding	R	1	1	1	1	1
GO:0031984	organelle subcompartment	M	1	1	1	1	1
GO:0071840	cellular component organization or biogenesis	R	1	1	1	1	1
GO:0042446	hormone biosynthetic process	C	1	1	1	1	1
GO:0022613	ribonucleoprotein complex biogenesis	J	1	1	1	1	1
GO:0009180	purine ribonucleoside diphosphate biosynthetic process	H, C	1	1	1	1	1
GO:0048487	beta-tubulin binding	Z	1	1	1	1	1
GO:0015591	D-ribose transmembrane transporter activity	G	1	1	1	1	1
GO:0052890	oxidoreductase activity, acting on the CH-CH group of donors, with a flavin as acceptor	C	1	1	1	1	1
GO:0005654	nucleoplasm	Y	1	1	1	1	1
GO:0044262	cellular carbohydrate metabolic process	R	1	1	1	1	1
GO:0016989	sigma factor antagonist activity	K	1	1	1	1	1
GO:0043168	anion binding	R	1	1	1	1	1
GO:0010562	positive regulation of phosphorus metabolic process	C	1	1	1	1	1
GO:0010558	negative regulation of macromolecule biosynthetic process	C	1	1	1	1	1
GO:0033365	protein localization to organelle	O	1	1	1	1	1
GO:0000776	kinetochore	Z	1	1	1	1	1
GO:0008210	estrogen metabolic process	I, C	1	1	1	1	1
GO:0046903	secretion	U	1	1	1	1	1
GO:0000922	spindle pole	Z	1	1	1	1	1
GO:0019203	carbohydrate phosphatase activity	C	1	1	1	1	1
GO:0018130	heterocycle biosynthetic process	H	1	1	1	1	1
GO:0042763	intracellular immature spore	R	1	1	1	1	1
GO:0010817	regulation of hormone levels	T	1	1	1	1	1
GO:0008081	phosphoric diester hydrolase activity	C	1	1	1	1	1
GO:0043230	extracellular organelle	W	1	1	1	1	1
GO:0050794	regulation of cellular process	T	1	1	1	1	1
GO:0046907	intracellular transport	U	1	1	1	1	1
GO:0003690	double-stranded DNA binding	B	1	1	1	1	1
GO:0140657	ATP-dependent activity	C	1	1	1	1	1
GO:0009736	cytokinin-activated signaling pathway	T	1	1	1	1	1
GO:0019062	virion attachment to host cell	X	1	1	1	1	1
GO:0030170	pyridoxal phosphate binding	Q	1	1	1	1	1
GO:0000811	GINS complex	B, Y	1	1	1	1	1
GO:0006576	biogenic amine metabolic process	C	1	1	1	1	1
GO:0008131	primary amine oxidase activity	E	1	1	1	1	1
GO:0007275	multicellular organism development	D	1	1	1	1	1
GO:0043958	acryloyl-CoA reductase (NADH) activity	H	1	1	1	1	1
GO:1901136	carbohydrate derivative catabolic process	G	1	1	1	1	1
GO:0032543	mitochondrial translation	J	1	1	1	1	1
GO:0016021	membrane	M	1	1	1	1	1
GO:1901605	alpha-amino acid metabolic process	E	1	1	1	1	1
GO:0043226	organelle	R	1	1	1	1	1
GO:0030017	sarcomere	Z	1	1	1	1	1
GO:0016788	hydrolase activity, acting on ester bonds	C	1	1	1	1	1
GO:0016823	hydrolase activity, acting on acid carbon-carbon bonds, in ketonic substances	C	1	1	1	1	1
GO:0050793	regulation of developmental process	T, D	1	1	1	1	1
GO:0009117	nucleotide metabolic process	F	1	1	1	1	1
GO:0016702	oxidoreductase activity, acting on single donors with incorporation of molecular oxygen, incorporation of two atoms of oxygen	C	1	1	1	1	1
GO:0006605	protein targeting	O	1	1	1	1	1
GO:0098791	Golgi apparatus subcompartment	M, U	1	1	1	1	1
GO:0042170	plastid membrane	M	1	1	1	1	1
GO:0042887	amide transmembrane transporter activity	U	1	1	1	1	1
GO:0035639	purine ribonucleoside triphosphate binding	F	1	1	1	1	1
GO:1903507	negative regulation of nucleic acid-templated transcription	R	1	1	1	1	1
GO:0006073	cellular glucan metabolic process	R	1	1	1	1	1
GO:0004673			1	1	1	1	1
GO:0019725	cellular homeostasis	R	1	1	1	1	1
GO:0140104	molecular carrier activity	R	1	1	1	1	1
GO:0008173	RNA methyltransferase activity	A	1	1	1	1	1
GO:0031975	envelope	M	1	1	1	1	1
GO:0072522	purine-containing compound biosynthetic process	H, C	1	1	1	1	1
GO:0045859	regulation of protein kinase activity	O	1	1	1	1	1
GO:0009705	plant-type vacuole membrane	U	1	1	1	1	1
GO:0004152	dihydroorotate dehydrogenase activity	C	1	1	1	1	1
GO:0043939	negative regulation of sporulation	D	1	1	1	1	1
GO:0016616	oxidoreductase activity, acting on the CH-OH group of donors, NAD or NADP as acceptor	C	1	1	1	1	1
GO:0006812	monoatomic cation transport	P	1	1	1	1	1
GO:0030141	secretory granule	M, U	1	1	1	1	1
GO:0033644	host cell membrane	R	1	1	1	1	1
GO:0099402	plant organ development	D	1	1	1	1	1
GO:0051252	regulation of RNA metabolic process	A	1	1	1	1	1
GO:0009000	selenocysteine lyase activity	C	1	1	1	1	1
GO:0009150	purine ribonucleotide metabolic process	F	1	1	1	1	1

GO_term	GO_term_name	COG_LETTER	*N. niacini*	*N. driksii* strains			
			DSM 2923T	179-C4-2-HS	.79-J 1A1 H:	AT2.8	V4I25
GO:0090482	vitamin transmembrane transporter activity	H, U	1	1	1	1	1
GO:0008152	metabolic process	C	1	1	1	1	1
GO:0016607	nuclear speck	Y	1	1	1	1	1
GO:0015718	monocarboxylic acid transport	C	1	1	1	1	1
GO:0009306	protein secretion	O	1	1	1	1	1
GO:0005743	mitochondrial inner membrane	M	1	1	1	1	1
GO:0015295	solute:proton symporter activity	P	1	1	1	1	1
GO:0051540			1	1	1	1	1
GO:0015075	monoatomic ion transmembrane transporter activity	U	1	1	1	1	1
GO:0019150	D-ribulokinase activity	P, T	1	1	1	1	1
GO:0120178	steroid hormone biosynthetic process	I	1	1	1	1	1
GO:0044403	biological process involved in symbiotic interaction	T	1	1	1	1	1
GO:0017111			1	1	1	1	1
GO:0042546	cell wall biogenesis	M	1	1	1	1	1
GO:0009236	cobalamin biosynthetic process	H, C	1	1	1	1	1
GO:0016071	mRNA metabolic process	A	1	1	1	1	1
GO:0000003	reproduction	D	1	1	1	1	1
GO:0034220	monoatomic ion transmembrane transport	P	1	1	1	1	1
GO:0030611	arsenate reductase activity	C	1	1	1	1	1
GO:0010501	RNA secondary structure unwinding	A	1	1	1	1	1
GO:0006950	response to stress	T	1	1	1	1	1
GO:0006414	translational elongation	J	1	1	1	1	1
GO:0019251	anaerobic cobalamin biosynthetic process	H, C	1	1	1	1	1
GO:0005667	transcription regulator complex	O	1	1	1	1	1
GO:0034071	aminoglycoside phosphotransferase activity	P, T	1	1	1	1	1
GO:0030435	sporulation resulting in formation of a cellular spore	D	1	1	1	1	1
GO:0016799	hydrolase activity, hydrolyzing N-glycosyl compounds	G	1	1	1	1	1
GO:0009890	negative regulation of biosynthetic process	C	1	1	1	1	1
GO:0006551	leucine metabolic process	E	1	1	1	1	1
GO:0000775	chromosome, centromeric region	B	1	1	1	1	1
GO:0006996	organelle organization	R	1	1	1	1	1
GO:0009260	ribonucleotide biosynthetic process	F	1	1	1	1	1
GO:0072330	monocarboxylic acid biosynthetic process	H	1	1	1	1	1
GO:0046365	monosaccharide catabolic process	H, G	1	1	1	1	1
GO:0046486	glycerolipid metabolic process	I	1	1	1	1	1
GO:0019114	catechol dioxygenase activity	C	1	1	1	1	1
GO:0032259	methylation	C	1	1	1	1	1
GO:0016774	phosphotransferase activity, carboxyl group as acceptor	P, T	1	1	1	1	1
GO:0042371	vitamin K biosynthetic process	H	1	1	1	1	1
GO:0009893	positive regulation of metabolic process	C	1	1	1	1	1
GO:0015666	restriction endodeoxyribonuclease activity	B	1	1	1	1	1
GO:0010605	negative regulation of macromolecule metabolic process	C	1	1	1	1	1
GO:0016671	oxidoreductase activity, acting on a sulfur group of donors, disulfide as acceptor	C	1	1	1	1	1
GO:0060090	molecular adaptor activity	R	1	1	1	1	1
GO:0016757	glycosyltransferase activity	G	1	1	1	1	1
GO:0005618	cell wall	W	1	1	1	1	1
GO:0052730	sarcosine N-methyltransferase activity	H	1	1	1	1	1
GO:0009165	nucleotide biosynthetic process	F	1	1	1	1	1
GO:1901566	organonitrogen compound biosynthetic process	C	1	1	1	1	1
GO:0043186	P granule	J	1	1	1	1	1
GO:0006575	cellular modified amino acid metabolic process	C	1	1	1	1	1
GO:0006714	sesquiterpenoid metabolic process	I	1	1	1	1	1
GO:0120025	plasma membrane bounded cell projection	M	1	1	1	1	1
GO:0006355	regulation of DNA-templated transcription	K	1	1	1	1	1
GO:0009755	hormone-mediated signaling pathway	T	1	1	1	1	1
GO:0015407	ABC-type monosaccharide transporter activity	G	1	1	1	1	1
GO:0060089	molecular transducer activity	T	1	1	1	1	1
GO:0016421	CoA carboxylase activity	H	1	1	1	1	1
GO:0010467	gene expression	K	1	1	1	1	1
GO:0009056	catabolic process	C	1	1	1	1	1
GO:0007049	cell cycle	R	1	1	1	1	1
GO:0004344	glucose dehydrogenase activity	C	1	1	1	1	1
GO:0140575	transmembrane monodehydroascorbate reductase activity	C	1	1	1	1	1
GO:0009185	ribonucleoside diphosphate metabolic process	H	1	1	1	1	1
GO:0043657	host cell	R	1	1	1	1	1
GO:0015144	carbohydrate transmembrane transporter activity	G	1	1	1	1	1
GO:0003824	catalytic activity	R	1	1	1	1	1
GO:0032879	regulation of localization	T	1	1	1	1	1
GO:0015931	nucleobase-containing compound transport	F	1	1	1	1	1
GO:0042327	positive regulation of phosphorylation	C	1	1	1	1	1
GO:0008104	protein localization	O	1	1	1	1	1
GO:0004396	hexokinase activity	P, T	1	1	1	1	1
GO:0008559	ABC-type xenobiotic transporter activity	Q, U	1	1	1	1	1
GO:0098803	respiratory chain complex	M, O	1	1	1	1	1
GO:0042995	cell projection	R	1	1	1	1	1
GO:0031461	cullin-RING ubiquitin ligase complex	O	1	1	1	1	1
GO:0006081	cellular aldehyde metabolic process	C	1	1	1	1	1

GO_term	GO_term_name	COG_LETTER	*N. niacini*	*N. driksii* strains			
			DSM 2923T	179-C4-2-HS	.79-J 1A1 H	AT2.8	V4I25
GO:0032774	RNA biosynthetic process	K	1	1	1	1	1
GO:0031323	regulation of cellular metabolic process	T, C	1	1	1	1	1
GO:0005739	mitochondrion	M	1	1	1	1	1
GO:0050790	regulation of catalytic activity	T	1	1	1	1	1
GO:1901362	organic cyclic compound biosynthetic process	C	1	1	1	1	1
GO:0033554	cellular response to stress	T	1	1	1	1	1
GO:0044275	cellular carbohydrate catabolic process	R	1	1	1	1	1
GO:0048523	negative regulation of cellular process	T	1	1	1	1	1
GO:0060341	regulation of cellular localization	U	1	1	1	1	1
GO:0032440	2-alkenal reductase [NAD(P)+] activity	C	1	1	1	1	1
GO:0046873	metal ion transmembrane transporter activity	P	1	1	1	1	1
GO:0070013	intracellular organelle lumen	M	1	1	1	1	1
GO:0004030	aldehyde dehydrogenase [NAD(P)+] activity	C	1	1	1	1	1
GO:0032880	regulation of protein localization	O	1	1	1	1	1
GO:0006720	isoprenoid metabolic process	I	1	1	1	1	1
GO:0019120	hydrolase activity, acting on acid halide bonds, in C-halide compounds	C	1	1	1	1	1
GO:0016410	N-acyltransferase activity	I	1	1	1	1	1
GO:0015768	maltose transport	G	1	1	1	1	1
GO:0051247	positive regulation of protein metabolic process	O	1	1	1	1	1
GO:0051536			1	1	1	1	1
GO:0016798	hydrolase activity, acting on glycosyl bonds	G	1	1	1	1	1
GO:0043038	amino acid activation	E	1	1	1	1	1
GO:0007062	sister chromatid cohesion	B, D	1	1	1	1	1
GO:0052689	carboxylic ester hydrolase activity	C	1	1	1	1	1
GO:0019898	extrinsic component of membrane	M	1	1	1	1	1
GO:0030312	external encapsulating structure	W	1	1	1	1	1
GO:0032991	protein-containing complex	O	1	1	1	1	1
GO:0099080	supramolecular complex	R	1	1	1	1	1
GO:0008796	bis(5'-nucleosyl)-tetraphosphatase activity	C	1	1	1	1	1
GO:0000155			1	1	1	1	1
GO:0140110	transcription regulator activity	K	1	1	1	1	1
GO:0032502	developmental process	D	1	1	1	1	1
GO:0036260	RNA capping	A	1	1	1	1	1
GO:0030424	axon	M	1	1	1	1	1
GO:0006397	mRNA processing	A	1	1	1	1	1
GO:0016070	RNA metabolic process	A	1	1	1	1	1
GO:0030234	enzyme regulator activity	T	1	1	1	1	1
GO:0090304	nucleic acid metabolic process	F	1	1	1	1	1
GO:0070125	mitochondrial translational elongation	J	1	1	1	1	1
GO:0019222	regulation of metabolic process	T, C	1	1	1	1	1
GO:0016323	basolateral plasma membrane	M	1	1	1	1	1
GO:0009501	amyloplast	M	1	1	1	1	1
GO:0050066	lysine 2,3-aminomutase activity	E	1	1	1	1	1
GO:0016817	hydrolase activity, acting on acid anhydrides	C	1	1	1	1	1
GO:0035461	vitamin transmembrane transport	H	1	1	1	1	1
GO:0042168	heme metabolic process	H	1	1	1	1	1
GO:0050896	response to stimulus	T	1	1	1	1	1
GO:0016835	carbon-oxygen lyase activity	C	1	1	1	1	1
GO:0043891	glyceraldehyde-3-phosphate dehydrogenase (NAD(P)+) (phosphorylating) activity	C	1	1	1	1	1
GO:0030687	preribosome, large subunit precursor	O	1	1	1	1	1
GO:0009987	cellular process	R	1	1	1	1	1
GO:0009132	nucleoside diphosphate metabolic process	H	1	1	1	1	1
GO:0019541	propionate metabolic process	I	1	1	1	1	1
GO:0042174	negative regulation of sporulation resulting in formation of a cellular spore	D	1	1	1	1	1
GO:0098660	inorganic ion transmembrane transport	P	1	1	1	1	1
GO:0016289	CoA hydrolase activity	H	1	1	1	1	1
GO:0020015	glycosome	M	1	1	1	1	1
GO:0035770	ribonucleoprotein granule	J	1	1	1	1	1
GO:0009725	response to hormone	T	1	1	1	1	1
GO:0016018	cyclosporin A binding	Q	1	1	1	1	1
GO:0046943	carboxylic acid transmembrane transporter activity	U, C	1	1	1	1	1
GO:0036094	small molecule binding	R	1	1	1	1	1
GO:0022414	reproductive process	D	1	1	1	1	1
GO:0098542	defense response to other organism	V	1	1	1	1	1
GO:0044255	cellular lipid metabolic process	I	1	1	1	1	1
GO:0006775	fat-soluble vitamin metabolic process	H	1	1	1	1	1
GO:1901137	carbohydrate derivative biosynthetic process	G	1	1	1	1	1
GO:0004812			1	1	1	1	1
GO:0047400	phosphonoacetate hydrolase activity	C	1	1	1	1	1
GO:0022853	active monoatomic ion transmembrane transporter activity	U	1	1	1	1	1
GO:0004145	diamine N-acetyltransferase activity	I	1	1	1	1	1
GO:0048870	cell motility	N	1	1	1	1	1
GO:0016814	hydrolase activity, acting on carbon-nitrogen (but not peptide) bonds, in cyclic amidines	C	1	1	1	1	1
GO:0004674	protein serine/threonine kinase activity	O	1	1	1	1	1

GO_term	GO_term_name	COG_LETTER	*N. niacini*	*N. driksii* strains			
			DSM 2923T	179-C4-2-HS	.79-J 1A1 H:	AT2.8	V4I25
GO:0009259	ribonucleotide metabolic process	F	1	1	1	1	1
GO:0004161	dimethylallyltranstransferase activity	I	1	1	1	1	1
GO:0001516	prostaglandin biosynthetic process	H, I	1	1	1	1	1
GO:0031983	vesicle lumen	M	1	1	1	1	1
GO:0016646	oxidoreductase activity, acting on the CH-NH group of donors, NAD or NADP as acceptor	C	1	1	1	1	1
GO:0044248	cellular catabolic process	C	1	1	1	1	1
GO:0061458	reproductive system development	D	1	1	1	1	1
GO:0035592	establishment of protein localization to extracellular region	O	1	1	1	1	1
GO:0098588	bounding membrane of organelle	M	1	1	1	1	1
GO:0033643	host cell part	R	1	1	1	1	1
GO:0046165	alcohol biosynthetic process	H	1	1	1	1	1
GO:0005761	mitochondrial ribosome	J	1	1	1	1	1
GO:0019751	polyol metabolic process	H	1	1	1	1	1
GO:0005849	mRNA cleavage factor complex	Y, O	1	1	1	1	1
GO:0016893	endonuclease activity, active with either ribo- or deoxyribonucleic acids and producing 5'-phosphomonoesters	C	1	1	1	1	1
GO:0043718	2-hydroxymethylglutarate dehydrogenase activity	E	1	1	1	1	1
GO:0032555	purine ribonucleotide binding	F, G	1	1	1	1	1
GO:0043412	macromolecule modification	C	1	1	1	1	1
GO:0008514	organic anion transmembrane transporter activity	U	1	1	1	1	1
GO:0046470	phosphatidylcholine metabolic process	C	1	1	1	1	1
GO:0046031	ADP metabolic process	F	1	1	1	1	1
GO:0051173	positive regulation of nitrogen compound metabolic process	C	1	1	1	1	1
GO:0003905	alkylbase DNA N-glycosylase activity	B	1	1	1	1	1
GO:0043227	membrane-bounded organelle	M	1	1	1	1	1
GO:0033646	host intracellular part	R	1	1	1	1	1
GO:0016627	oxidoreductase activity, acting on the CH-CH group of donors	C	1	1	1	1	1
GO:0016853	isomerase activity	R	1	1	1	1	1
GO:0006351	DNA-templated transcription	K	1	1	1	1	1
GO:0003676	nucleic acid binding	F	1	1	1	1	1
GO:0006693	prostaglandin metabolic process	I	1	1	1	1	1
GO:0015697	quaternary ammonium group transport	R	1	1	1	1	1
GO:0031123	RNA 3'-end processing	A	1	1	1	1	1
GO:0005615	extracellular space	W	1	1	1	1	1
GO:0031124	mRNA 3'-end processing	A	1	1	1	1	1
GO:0018995	host cellular component	R	1	1	1	1	1
GO:0051707	response to other organism	T	1	1	1	1	1
GO:0016662	oxidoreductase activity, acting on other nitrogenous compounds as donors, cytochrome as acceptor	C	1	1	1	1	1
GO:0008195	phosphatidate phosphatase activity	I	1	1	1	1	1
GO:0015035	protein-disulfide reductase activity	O	1	1	1	1	1
GO:0006692	prostanoid metabolic process	I	1	1	1	1	1
GO:0046915	transition metal ion transmembrane transporter activity	P	1	1	1	1	1
GO:1901474	azole transmembrane transporter activity	U	1	1	1	1	1
GO:0106068	SUMO ligase complex	O	1	1	1	1	1
GO:0031969	chloroplast membrane	M	1	1	1	1	1
GO:0005746	mitochondrial respirasome	M	1	1	1	1	1
GO:1901565	organonitrogen compound catabolic process	C	1	1	1	1	1
GO:0044271	cellular nitrogen compound biosynthetic process	C	1	1	1	1	1
GO:0034645	cellular macromolecule biosynthetic process	R	1	1	1	1	1
GO:0009225	nucleotide-sugar metabolic process	H, G	1	1	1	1	1
GO:0043604	amide biosynthetic process	C	1	1	1	1	1
GO:0022857	transmembrane transporter activity	U	1	1	1	1	1
GO:0042181	ketone biosynthetic process	H	1	1	1	1	1
GO:0050538	N-carbamoyl-L-amino-acid hydrolase activity	C	1	1	1	1	1
GO:0097659	nucleic acid-templated transcription	R	1	1	1	1	1
GO:0004521	RNA endonuclease activity	A	1	1	1	1	1
GO:1904680	peptide transmembrane transporter activity	U	1	1	1	1	1
GO:0042180	cellular ketone metabolic process	H	1	1	1	1	1
GO:0016831	carboxy-lyase activity	C	1	1	1	1	1
GO:0031261	DNA replication preinitiation complex	Y	1	1	1	1	1
GO:0051347	positive regulation of transferase activity	T	1	1	1	1	1
GO:0005548	phospholipid transporter activity	I	1	1	1	1	1
GO:0044164	host cell cytosol	R	1	1	1	1	1
GO:0009179	purine ribonucleoside diphosphate metabolic process	H	1	1	1	1	1
GO:0042254	ribosome biogenesis	J	1	1	1	1	1
GO:1901617	organic hydroxy compound biosynthetic process	C	1	1	1	1	1
GO:0030427	site of polarized growth	D	1	1	1	1	1
GO:0102786	stearoyl-[acp] desaturase activity	Q	1	1	1	1	1
GO:0042350	GDP-L-fucose biosynthetic process	H, G, C	1	1	1	1	1
GO:0005102	signaling receptor binding	T	1	1	1	1	1
GO:0099512	supramolecular fiber	R	1	1	1	1	1
GO:0007031	peroxisome organization	R	1	1	1	1	1
GO:0005856	cytoskeleton	Z	1	1	1	1	1
GO:0031974	membrane-enclosed lumen	M	1	1	1	1	1
GO:0004589	dihydroorotate dehydrogenase (NADH) activity	C	1	1	1	1	1
GO:0051338	regulation of transferase activity	T	1	1	1	1	1

GO_term	GO_term_name	COG_LETTER	*N. niacini*	*N. driksii* strains			
			DSM 2923T	179-C4-2-HS	.79-J 1A1 H:	AT2.8	V4I25
GO:0006865	amino acid transport	E	1	1	1	1	1
GO:0033865	nucleoside bisphosphate metabolic process	H	1	1	1	1	1
GO:0004386	helicase activity	F	1	1	1	1	1
GO:0034654	nucleobase-containing compound biosynthetic process	H, C	1	1	1	1	1
GO:0097367	carbohydrate derivative binding	G	1	1	1	1	1
GO:0009706	chloroplast inner membrane	M	1	1	1	1	1
GO:0016054	organic acid catabolic process	H	1	1	1	1	1
GO:0009528	plastid inner membrane	M	1	1	1	1	1
GO:0051179	localization	R	1	1	1	1	1
GO:1901606	alpha-amino acid catabolic process	E	1	1	1	1	1
GO:0034062	5'-3' RNA polymerase activity	A	1	1	1	1	1
GO:1903008	organelle disassembly	R	1	1	1	1	1
GO:0016869	intramolecular transferase activity, transferring amino groups	R	1	1	1	1	1
GO:0015838	amino-acid betaine transport	E	1	1	1	1	1
GO:0008080	N-acetyltransferase activity	I	1	1	1	1	1
GO:0009941	chloroplast envelope	M	1	1	1	1	1
GO:0046456	icosanoid biosynthetic process	H	1	1	1	1	1
GO:0022607	cellular component assembly	R	1	1	1	1	1
GO:0044233	mitochondria-associated endoplasmic reticulum membrane	R	1	1	1	1	1
GO:0000400	four-way junction DNA binding	L	1	1	1	1	1
GO:0071103	DNA conformation change	B	1	1	1	1	1
GO:0010468	regulation of gene expression	K	1	1	1	1	1
GO:0099513	polymeric cytoskeletal fiber	Z	1	1	1	1	1
GO:0016822	hydrolase activity, acting on acid carbon-carbon bonds	C	1	1	1	1	1
GO:0042373	vitamin K metabolic process	H	1	1	1	1	1
GO:1901702	salt transmembrane transporter activity	P	1	1	1	1	1
GO:0015145	monosaccharide transmembrane transporter activity	G	1	1	1	1	1
GO:0005681	spliceosomal complex	Y, O	1	1	1	1	1
GO:0015101	organic cation transmembrane transporter activity	U	1	1	1	1	1
GO:0009735	response to cytokinin	T	1	1	1	1	1
GO:0016491	oxidoreductase activity	C	1	1	1	1	1
GO:0009507	chloroplast	M	1	1	1	1	1
GO:0043231	intracellular membrane-bounded organelle	M	1	1	1	1	1
GO:0033013	tetrapyrrole metabolic process	H	1	1	1	1	1
GO:0016875	ligase activity, forming carbon-oxygen bonds	R	1	1	1	1	1
GO:0050580	2,5-didehydrogluconate reductase activity	C	1	1	1	1	1
GO:0016787	hydrolase activity	C	1	1	1	1	1
GO:0030684	preribosome	O	1	1	1	1	1
GO:0035251	UDP-glucosyltransferase activity	G	1	1	1	1	1
GO:0071897	DNA biosynthetic process	L	1	1	1	1	1
GO:0009081	branched-chain amino acid metabolic process	E	1	1	1	1	1
GO:0005310	dicarboxylic acid transmembrane transporter activity	C	1	1	1	1	1
GO:0030115	S-layer	W	1	1	1	1	1
GO:0036382	flavin reductase (NADH) activity	C	1	1	1	1	1
GO:0043167	ion binding	R	1	1	1	1	1
GO:0006357	regulation of transcription by RNA polymerase II	K	1	1	1	1	1
GO:0044282	small molecule catabolic process	H	1	1	1	1	1
GO:0018478	malonate-semialdehyde dehydrogenase (acetylating) activity	C	1	1	1	1	1
GO:0045860	positive regulation of protein kinase activity	O	1	1	1	1	1
GO:0005819	spindle	Z	1	1	1	1	1
GO:0016731	oxidoreductase activity, acting on iron-sulfur proteins as donors, NAD or NADP as acceptor	C	1	1	1	1	1
GO:0006820	monoatomic anion transport	P	1	1	1	1	1
GO:0005634	nucleus	Y	1	1	1	1	1
GO:0016701	oxidoreductase activity, acting on single donors with incorporation of molecular oxygen	C	1	1	1	1	1
GO:0043107	type IV pilus-dependent motility	N	1	1	1	1	1
GO:0016860	intramolecular oxidoreductase activity	C	1	1	1	1	1
GO:0003735			1	1	1	1	1
GO:0008610	lipid biosynthetic process	I	1	1	1	1	1
GO:0065003	protein-containing complex assembly	O	1	1	1	1	1
GO:0019985	translesion synthesis	L	1	1	1	1	1
GO:0006418			1	1	1	1	1
GO:0044232	organelle membrane contact site	R	1	1	1	1	1
GO:0005816	spindle pole body	Z	1	1	1	1	1
GO:0048583	regulation of response to stimulus	T	1	1	1	1	1
GO:1901698	response to nitrogen compound	T	1	1	1	1	1
GO:0004528	phosphodiesterase I activity	C	1	1	1	1	1
GO:0016782	transferase activity, transferring sulphur-containing groups	R	1	1	1	1	1
GO:0016672	oxidoreductase activity, acting on a sulfur group of donors, quinone or similar compound as acceptor	C	1	1	1	1	1
GO:0009791	post-embryonic development	D	1	1	1	1	1
GO:0016775	phosphotransferase activity, nitrogenous group as acceptor	P, T	1	1	1	1	1
GO:0048519	negative regulation of biological process	T	1	1	1	1	1
GO:0098772	molecular function regulator activity	T	1	1	1	1	1
GO:0032790	ribosome disassembly	J	1	1	1	1	1
GO:0034655	nucleobase-containing compound catabolic process	C	1	1	1	1	1

GO_term	GO_term_name	COG_LETTER	*N. niacini*	*N. driksii* strains			
			DSM 2923T	179-C4-2-HS	.79-J 1A1 H	AT2.8	V4I25
GO:0016818	hydrolase activity, acting on acid anhydrides, in phosphorus-containing anhydrides	C	1	1	1	1	1
GO:0047589	5-aminovalerate transaminase activity	E	1	1	1	1	1
GO:0004540	ribonuclease activity	A	1	1	1	1	1
GO:0016899	oxidoreductase activity, acting on the CH-OH group of donors, oxygen as acceptor	C	1	1	1	1	1
GO:0062023	collagen-containing extracellular matrix	W	1	1	1	1	1
GO:1901293	nucleoside phosphate biosynthetic process	H, C	1	1	1	1	1
GO:1901576	organic substance biosynthetic process	C	1	1	1	1	1
GO:0003859	3-hydroxybutyryl-CoA dehydratase activity	R	1	1	1	1	1
GO:0047429	nucleoside triphosphate diphosphatase activity	C	1	1	1	1	1
GO:0031312	extrinsic component of organelle membrane	M	1	1	1	1	1
GO:0008175	tRNA methyltransferase activity	J, A	1	1	1	1	1
GO:0097351	toxin sequestering activity	V	1	1	1	1	1
GO:0015294	solute:monoatomic cation symporter activity	P	1	1	1	1	1
GO:0016885	ligase activity, forming carbon-carbon bonds	R	1	1	1	1	1
GO:0015935	small ribosomal subunit	J	1	1	1	1	1
GO:0140513	nuclear protein-containing complex	Y, O	1	1	1	1	1
GO:0005759	mitochondrial matrix	M	1	1	1	1	1
GO:0030430	host cell cytoplasm	R	1	1	1	1	1
GO:0043656	host intracellular region	R	1	1	1	1	1
GO:0048608	reproductive structure development	D	1	1	1	1	1
GO:0012506	vesicle membrane	M	1	1	1	1	1
GO:0001067	transcription regulatory region nucleic acid binding	K	1	1	1	1	1
GO:0046394	carboxylic acid biosynthetic process	H	1	1	1	1	1
GO:0046149	pigment catabolic process	H	1	1	1	1	1
GO:0032940	secretion by cell	U	1	1	1	1	1
GO:0005198	structural molecule activity	R	1	1	1	1	1
GO:0009295	nucleoid	J	1	1	1	1	1
GO:0015171	amino acid transmembrane transporter activity	E	1	1	1	1	1
GO:0043755	alpha-ribazole phosphatase activity	C	1	1	1	1	1
GO:0016827	hydrolase activity, acting on acid carbon-phosphorus bonds	C	1	1	1	1	1
GO:0016857	racemase and epimerase activity, acting on carbohydrates and derivatives	E	1	1	1	1	1
GO:0019408	dolichol biosynthetic process	Q	1	1	1	1	1
GO:0071310	cellular response to organic substance	T	1	1	1	1	1
GO:0015333	peptide:proton symporter activity	P, U	1	1	1	1	1
GO:0016604	nuclear body	Y	1	1	1	1	1
GO:0071704	organic substance metabolic process	C	1	1	1	1	1
GO:0008289	lipid binding	I	1	1	1	1	1
GO:0005996	monosaccharide metabolic process	H, G	1	1	1	1	1
GO:0008643	carbohydrate transport	G	1	1	1	1	1
GO:0034641	cellular nitrogen compound metabolic process	C	1	1	1	1	1
GO:0009059	macromolecule biosynthetic process	C	1	1	1	1	1
GO:0051049	regulation of transport	T	1	1	1	1	1
GO:0045934	negative regulation of nucleobase-containing compound metabolic process	C	1	1	1	1	1
GO:0005923	bicellular tight junction	W	1	1	1	1	1
GO:0043937	regulation of sporulation	T, D	1	1	1	1	1
GO:0043178	alcohol binding	C	1	1	1	1	1
GO:0045152	antisigma factor binding	T	1	1	1	1	1
GO:0006552	leucine catabolic process	E	1	1	1	1	1
GO:0140096	catalytic activity, acting on a protein	O	1	1	1	1	1
GO:0005622	intracellular anatomical structure	R	1	1	1	1	1
GO:0019538	protein metabolic process	O	1	1	1	1	1
GO:0005524			1	1	1	1	1
GO:0042645	mitochondrial nucleoid	J	1	1	1	1	1
GO:1901661	quinone metabolic process	H	1	1	1	1	1
GO:0044281	small molecule metabolic process	H	1	1	1	1	1
GO:0072521	purine-containing compound metabolic process	C	1	1	1	1	1
GO:0046942	carboxylic acid transport	C	1	1	1	1	1
GO:0042374	phylloquinone metabolic process	H	1	1	1	1	1
GO:0032501	multicellular organismal process	R	1	1	1	1	1
GO:0099081	supramolecular polymer	R	1	1	1	1	1
GO:1990204	oxidoreductase complex	O	1	1	1	1	1
GO:0019213	deacetylase activity	C	1	1	1	1	1
GO:0022832	voltage-gated channel activity	U	1	1	1	1	1
GO:0006164	purine nucleotide biosynthetic process	F, H, C	1	1	1	1	1
GO:0051094	positive regulation of developmental process	D	1	1	1	1	1
GO:0070279			1	1	1	1	1
GO:1905348	endonuclease complex	O	1	1	1	1	1
GO:0016726	oxidoreductase activity, acting on CH or CH2 groups, NAD or NADP as acceptor	C	1	1	1	1	1
GO:1901615	organic hydroxy compound metabolic process	C	1	1	1	1	1
GO:0030054	cell junction	W	1	1	1	1	1
GO:0009066	aspartate family amino acid metabolic process	E	1	1	1	1	1
GO:0000731	DNA synthesis involved in DNA repair	L	1	1	1	1	1
GO:0003682	chromatin binding	B	1	1	1	1	1

GO_term	GO_term_name	COG_LETTER	*N. niacini*	*N. driksii* strains			
			DSM 2923T	179-C4-2-HS	.79-J 1A1 H:	AT2.8	V4I25
GO:0044406	adhesion of symbiont to host	T	1	1	1	1	1
GO:2000113	negative regulation of cellular macromolecule biosynthetic process	R	1	1	1	1	1
GO:0003714	transcription corepressor activity	K	1	1	1	1	1
GO:0070069	cytochrome complex	O	1	1	1	1	1
GO:0016730	oxidoreductase activity, acting on iron-sulfur proteins as donors	C	1	1	1	1	1
GO:0047922	gentisate 1,2-dioxygenase activity	C	1	1	1	1	1
GO:0030162	regulation of proteolysis	O	1	1	1	1	1
GO:0009133	nucleoside diphosphate biosynthetic process	H, C	1	1	1	1	1
GO:0044238	primary metabolic process	C	1	1	1	1	1
GO:0042173	regulation of sporulation resulting in formation of a cellular spore	T, D	1	1	1	1	1
GO:0015662	P-type ion transporter activity	C	1	1	1	1	1
GO:0051276	chromosome organization	B	1	1	1	1	1
GO:0006811	monoatomic ion transport	P	1	1	1	1	1
GO:0004033	aldo-keto reductase (NADP) activity	C	1	1	1	1	1
GO:0045178	basal part of cell	M	1	1	1	1	1
GO:0016791	phosphatase activity	C	1	1	1	1	1
GO:0051129	negative regulation of cellular component organization	T	1	1	1	1	1
GO:0048871	multicellular organismal-level homeostasis	R	1	1	1	1	1
GO:0006753	nucleoside phosphate metabolic process	H	1	1	1	1	1
GO:1901361	organic cyclic compound catabolic process	C	1	1	1	1	1
GO:1901981	phosphatidylinositol phosphate binding	I	1	1	1	1	1
GO:1901505	carbohydrate derivative transmembrane transporter activity	G, U	1	1	1	1	1
GO:0009328	phenylalanine-tRNA ligase complex	O	1	1	1	1	1
GO:0072341	modified amino acid binding	E	1	1	1	1	1
GO:0042362	fat-soluble vitamin biosynthetic process	H	1	1	1	1	1
GO:0042221	response to chemical	T	1	1	1	1	1
GO:0000785	chromatin	B	1	1	1	1	1
GO:0042578	phosphoric ester hydrolase activity	C	1	1	1	1	1
GO:0016423	tRNA (guanine) methyltransferase activity	H	1	1	1	1	1
GO:0043733	DNA-3-methylbase glycosylase activity	B	1	1	1	1	1
GO:0043244	regulation of protein-containing complex disassembly	O	1	1	1	1	1
GO:0015343	siderophore-iron transmembrane transporter activity	U	1	1	1	1	1
GO:0006082	organic acid metabolic process	H	1	1	1	1	1
GO:0048856	anatomical structure development	D	1	1	1	1	1
GO:0031410	cytoplasmic vesicle	U	1	1	1	1	1
GO:0051119	sugar transmembrane transporter activity	G	1	1	1	1	1
GO:0031324	negative regulation of cellular metabolic process	C	1	1	1	1	1
GO:0036464	cytoplasmic ribonucleoprotein granule	J	1	1	1	1	1
GO:0006766	vitamin metabolic process	H	1	1	1	1	1
GO:0044391	ribosomal subunit	J	1	1	1	1	1
GO:0009188	ribonucleoside diphosphate biosynthetic process	H, C	1	1	1	1	1
GO:0008106	alcohol dehydrogenase (NADP+) activity	C	1	1	1	1	1
GO:0000315	organellar large ribosomal subunit	J	1	1	1	1	1
GO:0071368	cellular response to cytokinin stimulus	T	1	1	1	1	1
GO:0006508	proteolysis	O	1	1	1	1	1
GO:0016866	intramolecular transferase activity	R	1	1	1	1	1
GO:0050632	propionyl-CoA C2-trimethyltridecanoyltransferase activity	I	1	1	1	1	1
GO:0019348	dolichol metabolic process	H	1	1	1	1	1
GO:0015165	pyrimidine nucleotide-sugar transmembrane transporter activity	F	1	1	1	1	1
GO:0008528	G protein-coupled peptide receptor activity	T	1	1	1	1	1
GO:0015740	C4-dicarboxylate transport	C	1	1	1	1	1
GO:0051246	regulation of protein metabolic process	O	1	1	1	1	1
GO:0071705	nitrogen compound transport	R	1	1	1	1	1
GO:0070461	SAGA-type complex	B	1	1	1	1	1
GO:0098800	inner mitochondrial membrane protein complex	M, O	1	1	1	1	1
GO:0006066	alcohol metabolic process	H	1	1	1	1	1
GO:0000217	DNA secondary structure binding	B	1	1	1	1	1
GO:0015078	proton transmembrane transporter activity	P	1	1	1	1	1
GO:0043228	non-membrane-bounded organelle	R	1	1	1	1	1
GO:0061634	alpha-D-xyloside xylohydrolase	G	1	1	1	1	1
GO:1901607	alpha-amino acid biosynthetic process	E	1	1	1	1	1
GO:0051180	vitamin transport	H	1	1	1	1	1
GO:1905368	peptidase complex	O	1	1	1	1	1
GO:0008206	bile acid metabolic process	I	1	1	1	1	1
GO:0031252	cell leading edge	N	1	1	1	1	1
GO:0003677	DNA binding	B	1	1	1	1	1
GO:0046457	prostanoid biosynthetic process	H, I	1	1	1	1	1
GO:0006810	transport	R	1	1	1	1	1
GO:0033014	tetrapyrrole biosynthetic process	H, C	1	1	1	1	1
GO:0043436	oxoacid metabolic process	H	1	1	1	1	1
GO:0098662	inorganic cation transmembrane transport	P	1	1	1	1	1
GO:0016310	phosphorylation	C	1	1	1	1	1
GO:0008757	S-adenosylmethionine-dependent methyltransferase activity	H	1	1	1	1	1
GO:0016903	oxidoreductase activity, acting on the aldehyde or oxo group of donors	C	1	1	1	1	1
GO:0004888	transmembrane signaling receptor activity	T	1	1	1	1	1
GO:0009653	anatomical structure morphogenesis	D	1	1	1	1	1
GO:0009889	regulation of biosynthetic process	T, C	1	1	1	1	1

GO_term	GO_term_name	COG_LETTER	*N. niacini*	*N. driksii* strains			
			DSM 2923T	179-C4-2-HS	.79-J 1A1 H:	AT2.8	V4I25
GO:0019239	deaminase activity	C	1	1	1	1	1
GO:0003995	acyl-CoA dehydrogenase activity	H	1	1	1	1	1
GO:0042372	phylloquinone biosynthetic process	H	1	1	1	1	1
GO:0038023	signaling receptor activity	T	1	1	1	1	1
GO:0015849	organic acid transport	R	1	1	1	1	1
GO:0006974	cellular response to DNA damage stimulus	T	1	1	1	1	1
GO:0009226	nucleotide-sugar biosynthetic process	H, G, C	1	1	1	1	1
GO:0042625			1	1	1	1	1
GO:0072594	establishment of protein localization to organelle	O	1	1	1	1	1
GO:0008876	quinoprotein glucose dehydrogenase activity	C	1	1	1	1	1
GO:0072524	pyridine-containing compound metabolic process	C	1	1	1	1	1
GO:0006796	phosphate-containing compound metabolic process	C	1	1	1	1	1
GO:0007165	signal transduction	T	1	1	1	1	1
GO:0098590	plasma membrane region	M	1	1	1	1	1
GO:0006520	amino acid metabolic process	E	1	1	1	1	1
GO:0016830	carbon-carbon lyase activity	C	1	1	1	1	1
GO:0000151	ubiquitin ligase complex	O	1	1	1	1	1
GO:0051726	regulation of cell cycle	T	1	1	1	1	1
GO:0006629	lipid metabolic process	I	1	1	1	1	1
GO:1905039	carboxylic acid transmembrane transport	C	1	1	1	1	1
GO:0045937	positive regulation of phosphate metabolic process	C	1	1	1	1	1
GO:0008875	gluconate dehydrogenase activity	C	1	1	1	1	1
GO:0046373	L-arabinose metabolic process	H, G	1	1	1	1	1
GO:0004518	nuclease activity	C	1	1	1	1	1
GO:0005543	phospholipid binding	I	1	1	1	1	1
GO:0006952	defense response	V	1	1	1	1	1
GO:0030915	Smc5-Smc6 complex	B	1	1	1	1	1
GO:0005783	endoplasmic reticulum	M, U	1	1	1	1	1
GO:0016645	oxidoreductase activity, acting on the CH-NH group of donors	C	1	1	1	1	1
GO:0016725	oxidoreductase activity, acting on CH or CH2 groups	C	1	1	1	1	1
GO:0071944	cell periphery	W	1	1	1	1	1
GO:0019058	viral life cycle	X	1	1	1	1	1
GO:0030426	growth cone	D	1	1	1	1	1
GO:0070726	cell wall assembly	M	1	1	1	1	1
GO:0016032	viral process	X	1	1	1	1	1
GO:0015234	thiamine transmembrane transporter activity	F, H, U	1	1	1	1	1
GO:0000976	transcription cis-regulatory region binding	K	1	1	1	1	1
GO:0015766	disaccharide transport	G	1	1	1	1	1
GO:0001934	positive regulation of protein phosphorylation	O	1	1	1	1	1
GO:0043933	protein-containing complex organization	O	1	1	1	1	1
GO:0043085	positive regulation of catalytic activity	T	1	1	1	1	1
GO:0071495	cellular response to endogenous stimulus	T	1	1	1	1	1
GO:0016670	oxidoreductase activity, acting on a sulfur group of donors, oxygen as acceptor	C	1	1	1	1	1
GO:0006366	transcription by RNA polymerase II	K	1	1	1	1	1
GO:0047046	homoisocitrate dehydrogenase activity	C	1	1	1	1	1
GO:0031327	negative regulation of cellular biosynthetic process	C	1	1	1	1	1
GO:0044650	adhesion of symbiont to host cell	T	1	1	1	1	1
GO:0015919	peroxisomal membrane transport	U	1	1	1	1	1
GO:0006518	peptide metabolic process	E, Q	1	1	1	1	1
GO:0045495	pole plasm	R	1	1	1	1	1
GO:0008194	UDP-glycosyltransferase activity	G	1	1	1	1	1
GO:0043822	ribonuclease M5 activity	A	1	1	1	1	1
GO:0005604	basement membrane	W	1	1	1	1	1
GO:0110165	cellular anatomical entity	R	1	1	1	1	1
GO:0046390	ribose phosphate biosynthetic process	G	1	1	1	1	1
GO:0034032	purine nucleoside bisphosphate metabolic process	H	1	1	1	1	1
GO:0032559	adenyl ribonucleotide binding	F, G	1	1	1	1	1
GO:0004321	fatty-acyl-CoA synthase activity	I	1	1	1	1	1
GO:0003006	developmental process involved in reproduction	D	1	1	1	1	1
GO:0000502	proteasome complex	O	1	1	1	1	1
GO:0000122	negative regulation of transcription by RNA polymerase II	K	1	1	1	1	1
GO:0004553	hydrolase activity, hydrolyzing O-glycosyl compounds	G	1	1	1	1	1
GO:0048513	animal organ development	D	1	1	1	1	1
GO:0006464			1	1	1	1	1
GO:0050501	hyaluronan synthase activity	G	1	1	1	1	1
GO:0031399	regulation of protein modification process	O	1	1	1	1	1
GO:0016462	pyrophosphatase activity	C	1	1	1	1	1
GO:0051213	dioxygenase activity	C	1	1	1	1	1
GO:0015879	carnitine transport	E	1	1	1	1	1
GO:1902493	acetyltransferase complex	O	1	1	1	1	1
GO:0022890	inorganic cation transmembrane transporter activity	P	1	1	1	1	1
GO:0009617	response to bacterium	T	1	1	1	1	1
GO:0005930	axoneme	Z	1	1	1	1	1
GO:0005342	organic acid transmembrane transporter activity	U, C	1	1	1	1	1
GO:0008143	poly(A) binding	F	1	1	1	1	1
GO:0003723	RNA binding	A	1	1	1	1	1
GO:0018685	alkane 1-monooxygenase activity	Q	1	1	1	1	1

GO_term	GO_term_name	COG_LETTER	*N. niacini*	*N. driksii* strains			
			DSM 2923T	179-C4-2-HS	.79-J 1A1 H:	AT2.8	V4I25
GO:0016231	beta-N-acetylglucosaminidase activity	G	1	1	1	1	1
GO:0070717	poly-purine tract binding	F	1	1	1	1	1
GO:0006694	steroid biosynthetic process	I	1	1	1	1	1
GO:0015036	disulfide oxidoreductase activity	C	1	1	1	1	1
GO:0019200	carbohydrate kinase activity	P, T, G	1	1	1	1	1
GO:0006793	phosphorus metabolic process	C	1	1	1	1	1
GO:0009925	basal plasma membrane	M	1	1	1	1	1
GO:0016667	oxidoreductase activity, acting on a sulfur group of donors	C	1	1	1	1	1
GO:0015185	gamma-aminobutyric acid transmembrane transporter activity	E	1	1	1	1	1
GO:0031090	organelle membrane	M	1	1	1	1	1
GO:0019568	arabinose catabolic process	H, G	1	1	1	1	1
GO:0015318	inorganic molecular entity transmembrane transporter activity	U	1	1	1	1	1
GO:0031559	oxidosqualene cyclase activity	Q	1	1	1	1	1
GO:0010646	regulation of cell communication	T	1	1	1	1	1
GO:0007154	cell communication	T	1	1	1	1	1
GO:0016811	hydrolase activity, acting on carbon-nitrogen (but not peptide) bonds, in linear amides	C	1	1	1	1	1
GO:0005929	cilium	M, O	1	1	1	1	1
GO:0098655	monoatomic cation transmembrane transport	P	1	1	1	1	1
GO:0009251	glucan catabolic process	G	1	1	1	1	1
GO:0016888	endodeoxyribonuclease activity, producing 5'-phosphomonoesters	B	1	1	1	1	1
GO:0015229	L-ascorbic acid transmembrane transporter activity	H, U	1	1	1	1	1
GO:0048522	positive regulation of cellular process	T	1	1	1	1	1
GO:0006636	unsaturated fatty acid biosynthetic process	I	1	1	1	1	1
GO:0043565	sequence-specific DNA binding	B	1	1	1	1	1
GO:0140359	ABC-type transporter activity	C	1	1	1	1	1
GO:0019220	regulation of phosphate metabolic process	T, C	1	1	1	1	1
GO:0015099	nickel cation transmembrane transporter activity	P	1	1	1	1	1
GO:0006725	cellular aromatic compound metabolic process	C	1	1	1	1	1
GO:0005838	proteasome regulatory particle	O	1	1	1	1	1
GO:0019219	regulation of nucleobase-containing compound metabolic process	T, C	1	1	1	1	1
GO:0072663	establishment of protein localization to peroxisome	O	1	1	1	1	1
GO:0098798	mitochondrial protein-containing complex	O	1	1	1	1	1
GO:0005515	protein binding	R	1	1	1	1	1
GO:0009452	7-methylguanosine RNA capping	A	1	1	1	1	1
GO:0006778	porphyrin-containing compound metabolic process	H	1	1	1	1	1
GO:0004519	endonuclease activity	C	1	1	1	1	1
GO:0097708	intracellular vesicle	U	1	1	1	1	1
GO:0016278	lysine N-methyltransferase activity	H	1	1	1	1	1
GO:0140098	catalytic activity, acting on RNA	A	1	1	1	1	1
GO:0000272	polysaccharide catabolic process	G	1	1	1	1	1
GO:0003974	UDP-N-acetylglucosamine 4-epimerase activity	F, G	1	1	1	1	1
GO:0051128	regulation of cellular component organization	T	1	1	1	1	1
GO:0016408	C-acyltransferase activity	I	1	1	1	1	1
GO:0051604	protein maturation	K, O	1	1	1	1	1
GO:0008170	N-methyltransferase activity	R	1	1	1	1	1
GO:0046700	heterocycle catabolic process	C	1	1	1	1	1
GO:0044085	cellular component biogenesis	R	1	1	1	1	1
GO:0005737	cytoplasm	R	1	1	1	1	1
GO:0004497	monooxygenase activity	C	1	1	1	1	1
GO:0005840	ribosome	J	1	1	1	1	1
GO:0042325	regulation of phosphorylation	T, C	1	1	1	1	1
GO:0070161	anchoring junction	W	1	1	1	1	1
GO:0008202	steroid metabolic process	I	1	1	1	1	1
GO:0071806	protein transmembrane transport	O	1	1	1	1	1
GO:0016872	intramolecular lyase activity	R	1	1	1	1	1
GO:0016747	acyltransferase activity, transferring groups other than amino-acyl groups	I	1	1	1	1	1
GO:0071396	cellular response to lipid	T	1	1	1	1	1
GO:0044260	cellular macromolecule metabolic process	R	1	1	1	1	1
GO:0097747	RNA polymerase activity	A	1	1	1	1	1
GO:1901700	response to oxygen-containing compound	T	1	1	1	1	1
GO:0018491	2-oxobutyrate synthase activity	C	1	1	1	1	1
GO:0140358	P-type transmembrane transporter activity	C	1	1	1	1	1
GO:0009308	amine metabolic process	C	1	1	1	1	1
GO:0006767	water-soluble vitamin metabolic process	H	1	1	1	1	1
GO:0043232	intracellular non-membrane-bounded organelle	R	1	1	1	1	1
GO:0043177	organic acid binding	R	1	1	1	1	1
GO:0046368	GDP-L-fucose metabolic process	H, G	1	1	1	1	1
GO:0043938	positive regulation of sporulation	D	1	1	1	1	1
GO:0031325	positive regulation of cellular metabolic process	C	1	1	1	1	1
GO:0019637	organophosphate metabolic process	C	1	1	1	1	1
GO:0009058	biosynthetic process	C	1	1	1	1	1
GO:2001141	regulation of RNA biosynthetic process	K	1	1	1	1	1
GO:0065002	intracellular protein transmembrane transport	O	1	1	1	1	1
GO:0030554	adenyl nucleotide binding	F	1	1	1	1	1
GO:0044743	protein transmembrane import into intracellular organelle	O	1	1	1	1	1
GO:0097014	ciliary plasm	M, O	1	1	1	1	1

GO_term	GO_term_name	COG_LETTER	*N. niacini*	*N. driksii* strains			
			DSM 2923T	179-C4-2-HS	.79-J 1A1 H:	AT2.8	V4I25
GO:0005576	extracellular region	W	1	1	1	1	1
GO:0065009	regulation of molecular function	T	1	1	1	1	1
GO:0004527	exonuclease activity	C	1	1	1	1	1
GO:0044092	negative regulation of molecular function	T	1	1	1	1	1
GO:1990904	ribonucleoprotein complex	O	1	1	1	1	1
GO:0016641	oxidoreductase activity, acting on the CH-NH2 group of donors, oxygen as acceptor	E	1	1	1	1	1
GO:0016043	cellular component organization	R	1	1	1	1	1
GO:0035539	8-oxo-7,8-dihydrodeoxyguanosine triphosphate pyrophosphatase activity	C	1	1	1	1	1
GO:0019207	kinase regulator activity	T	1	1	1	1	1
GO:0003700	DNA-binding transcription factor activity	K	1	1	1	1	1
GO:0008622	epsilon DNA polymerase complex	B, Y, O	1	1	1	1	1
GO:0004099	chitin deacetylase activity	C	1	1	1	1	1
GO:0015146	pentose transmembrane transporter activity	G	1	1	1	1	1
GO:0033875	ribonucleoside bisphosphate metabolic process	H	1	1	1	1	1
GO:0003727	single-stranded RNA binding	A	1	1	1	1	1
GO:0006399	tRNA metabolic process	J	1	1	1	1	1
GO:0018850	chloromuconate cycloisomerase activity	Q	1	1	1	1	1
GO:0004659	prenyltransferase activity	I	1	1	1	1	1
GO:0009136	purine nucleoside diphosphate biosynthetic process	H, C	1	1	1	1	1
GO:0140640	catalytic activity, acting on a nucleic acid	F	1	1	1	1	1
GO:0009605	response to external stimulus	T	1	1	1	1	1
GO:0051240	positive regulation of multicellular organismal process	T	1	1	1	1	1
GO:0006690	icosanoid metabolic process	H	1	1	1	1	1
GO:0004753	saccharopine dehydrogenase activity	C	1	1	1	1	1
GO:0051174	regulation of phosphorus metabolic process	T, C	1	1	1	1	1
GO:0042811	pheromone biosynthetic process	Q	1	1	1	1	1
GO:0015293	symporter activity	U	1	1	1	1	1
GO:0004520	DNA endonuclease activity	B	1	1	1	1	1
GO:0016901	oxidoreductase activity, acting on the CH-OH group of donors, quinone or similar compound as acceptor	C	1	1	1	1	1
GO:0015934	large ribosomal subunit	J	1	1	1	1	1
GO:0006787	porphyrin-containing compound catabolic process	H	1	1	1	1	1
GO:0140053	mitochondrial gene expression	K	1	1	1	1	1
GO:0016854	racemase and epimerase activity	R	1	1	1	1	1
GO:0015267	channel activity	U	1	1	1	1	1
GO:0006301	postreplication repair	L	1	1	1	1	1
GO:0019438	aromatic compound biosynthetic process	C	1	1	1	1	1
GO:0009966	regulation of signal transduction	T	1	1	1	1	1
GO:0034545	fumarylpyruvate hydrolase activity	C	1	1	1	1	1
GO:0016639	oxidoreductase activity, acting on the CH-NH2 group of donors, NAD or NADP as acceptor	E	1	1	1	1	1
GO:0005795	Golgi stack	M, U	1	1	1	1	1
GO:0016891	RNA endonuclease activity, producing 5'-phosphomonoesters	A	1	1	1	1	1
GO:0003877	ATP adenylyltransferase activity	P, T	1	1	1	1	1
GO:0009892	negative regulation of metabolic process	C	1	1	1	1	1
GO:0071555	cell wall organization	M	1	1	1	1	1
GO:0042601	endospore-forming forespore	D	1	1	1	1	1
GO:0016620	oxidoreductase activity, acting on the aldehyde or oxo group of donors, NAD or NADP as acceptor	C	1	1	1	1	1
GO:0016229	steroid dehydrogenase activity	Q	1	1	1	1	1
GO:0009123	nucleoside monophosphate metabolic process	H	1	1	1	1	1
GO:1901564	organonitrogen compound metabolic process	C	1	1	1	1	1
GO:0006699	bile acid biosynthetic process	I	1	1	1	1	1
GO:0097159	organic cyclic compound binding	R	1	1	1	1	1
GO:1901292	nucleoside phosphate catabolic process	H	1	1	1	1	1
GO:0010035	response to inorganic substance	T	1	1	1	1	1
GO:0070887	cellular response to chemical stimulus	T	1	1	1	1	1
GO:0019748	secondary metabolic process	Q	1	1	1	1	1
GO:0018669	3-hydroxybenzoate 6-monooxygenase activity	H	1	1	1	1	1
GO:0042445	hormone metabolic process	C	1	1	1	1	1
GO:0016234	inclusion body	R	1	1	1	1	1
GO:0016298	lipase activity	I	1	1	1	1	1
GO:0004039	allophanate hydrolase activity	C	1	1	1	1	1
GO:0016879	ligase activity, forming carbon-nitrogen bonds	R	1	1	1	1	1
GO:0043462	regulation of ATP-dependent activity	T, C	1	1	1	1	1
GO:0032870	cellular response to hormone stimulus	T	1	1	1	1	1
GO:0031966	mitochondrial membrane	M	1	1	1	1	1
GO:0050606	4-carboxy-2-hydroxymuconate semialdehyde hemiacetal dehydrogenase activity	C	1	1	1	1	1
GO:0016878	acid-thiol ligase activity	C	1	1	1	1	1
GO:0008509	monoatomic anion transmembrane transporter activity	P, U	1	1	1	1	1
GO:0004485	methylcrotonoyl-CoA carboxylase activity	H	1	1	1	1	1
GO:0016874	ligase activity	R	1	1	1	1	1
GO:0043798	glycerate 2-kinase activity	P, T	1	1	1	1	1
GO:0016966	nitric oxide reductase activity	C	1	1	1	1	1
GO:0009991	response to extracellular stimulus	T	1	1	1	1	1

GO_term	GO_term_name	COG_LETTER	*N. niacini*	*N. driksii* strains			
			DSM 2923T	179-C4-2-HS	.79-J 1A1 H	AT2.8	V4I25
GO:0015630	microtubule cytoskeleton	Z	1	1	1	1	1
GO:0042579	microbody	M	1	1	1	1	1
GO:0000808	origin recognition complex	B, O	1	1	1	1	1
GO:0055086	nucleobase-containing small molecule metabolic process	H	1	1	1	1	1
GO:0032993	protein-DNA complex	O	1	1	1	1	1
GO:0015771	trehalose transport	G	1	1	1	1	1
GO:0043229	intracellular organelle	R	1	1	1	1	1
GO:0030674	protein-macromolecule adaptor activity	O	1	1	1	1	1
GO:0044247	cellular polysaccharide catabolic process	R	1	1	1	1	1
GO:0035446	cysteine-glucosaminylinositol ligase activity	E	1	1	1	1	1
GO:0051641	cellular localization	U	1	1	1	1	1
GO:0004123	cystathionine gamma-lyase activity	C	1	1	1	1	1
GO:1901682	sulfur compound transmembrane transporter activity	U	1	1	1	1	1
GO:0015711	organic anion transport	R	1	1	1	1	1
GO:0060205	cytoplasmic vesicle lumen	U	1	1	1	1	1
GO:0016779	nucleotidyltransferase activity	P, T	1	1	1	1	1
GO:0042626	ATPase-coupled transmembrane transporter activity	C	1	1	1	1	1
GO:0022411	cellular component disassembly	R	1	1	1	1	1
GO:0016772	transferase activity, transferring phosphorus-containing groups	P, T	1	1	1	1	1
GO:0005319	lipid transporter activity	I	1	1	1	1	1
GO:0032182	ubiquitin-like protein binding	O	1	1	1	1	1
GO:0009607	response to biotic stimulus	T	1	1	1	1	1
GO:0030027	lamellipodium	N	1	1	1	1	1
GO:0043005	neuron projection	M	1	1	1	1	1
GO:0006396	RNA processing	A	1	1	1	1	1
GO:0005975	carbohydrate metabolic process	G	1	1	1	1	1
GO:0042810	pheromone metabolic process	Q	1	1	1	1	1
GO:1901575	organic substance catabolic process	C	1	1	1	1	1
GO:0009063	amino acid catabolic process	H, C	1	1	1	1	1
GO:0070469	respirasome	M	1	1	1	1	1
GO:2000112	regulation of cellular macromolecule biosynthetic process	R	1	1	1	1	1
GO:0050660			1	1	1	1	1
GO:0006259	DNA metabolic process	B	1	1	1	1	1
GO:0047372	acylglycerol lipase activity	I	1	1	1	1	1
GO:1901363	heterocyclic compound binding	R	1	1	1	1	1
GO:0016301	kinase activity	P, T	1	1	1	1	1
GO:0060293	germ plasm	R	1	1	1	1	1
GO:0019866	organelle inner membrane	M	1	1	1	1	1
GO:0048646	anatomical structure formation involved in morphogenesis	D	1	1	1	1	1
GO:0005751	mitochondrial respiratory chain complex IV	M, O	1	1	1	1	1
GO:0016836	hydro-lyase activity	C	1	1	1	1	1
GO:0010033	response to organic substance	T	1	1	1	1	1
GO:0006163	purine nucleotide metabolic process	F	1	1	1	1	1
GO:0043934	sporulation	D	1	1	1	1	1
GO:0001726	ruffle	N	1	1	1	1	1
GO:0006644	phospholipid metabolic process	I	1	1	1	1	1
GO:0016020	membrane	M	1	1	1	1	1
GO:0051093	negative regulation of developmental process	D	1	1	1	1	1
GO:1901663	quinone biosynthetic process	H	1	1	1	1	1
GO:0009699	phenylpropanoid biosynthetic process	Q, C	1	1	1	1	1
GO:0051253	negative regulation of RNA metabolic process	A	1	1	1	1	1
GO:0015297	antiporter activity	U	1	1	1	1	1
GO:0034219	carbohydrate transmembrane transport	G	1	1	1	1	1
GO:0033015	tetrapyrrole catabolic process	H	1	1	1	1	1
GO:0032553	ribonucleotide binding	F, G	1	1	1	1	1
GO:0016765	transferase activity, transferring alkyl or aryl (other than methyl) groups	R	1	1	1	1	1
GO:0140737	encapsulin nanocompartment	X	1	1	1	1	1
GO:0050062	long-chain-fatty-acyl-CoA reductase activity	C	1	1	1	1	1
GO:0072662	protein localization to peroxisome	O	1	1	1	1	1
GO:0034470	ncRNA processing	A	1	1	1	1	1
GO:0015291	secondary active transmembrane transporter activity	U	1	1	1	1	1
GO:0046912	acyltransferase activity, acyl groups converted into alkyl on transfer	C	1	1	1	1	1
GO:0016741	transferase activity, transferring one-carbon groups	R	1	1	1	1	1
GO:0008028	monocarboxylic acid transmembrane transporter activity	C	1	1	1	1	1
GO:0016052	carbohydrate catabolic process	G	1	1	1	1	1
GO:0043242	negative regulation of protein-containing complex disassembly	O	1	1	1	1	1
GO:1902679	negative regulation of RNA biosynthetic process	K	1	1	1	1	1
GO:0019875	6-aminohexanoate-dimer hydrolase activity	C	1	1	1	1	1
GO:0019104	DNA N-glycosylase activity	B	1	1	1	1	1
GO:0043549	regulation of kinase activity	P, T	1	1	1	1	1
GO:0015611	ABC-type D-ribose transporter activity	G	1	1	1	1	1
GO:0046434	organophosphate catabolic process	C	1	1	1	1	1
GO:0015399	primary active transmembrane transporter activity	U	1	1	1	1	1
GO:0043039			1	1	1	1	1
GO:0004857	enzyme inhibitor activity	T	1	1	1	1	1
GO:0016824	hydrolase activity, acting on acid halide bonds	C	1	1	1	1	1
GO:0098656			1	1	1	1	1

GO_term	GO_term_name	COG_LETTER	*N. niacini*	*N. driksii* strains			
			DSM 2923T	179-C4-2-HS	.79-J 1A1 H	AT2.8	V4I25
GO:1905369	endopeptidase complex	O	1	1	1	1	1
GO:0045862	positive regulation of proteolysis	O	1	1	1	1	1
GO:0006468	protein phosphorylation	O	1	1	1	1	1
GO:0005694	chromosome	B	1	1	1	1	1
GO:0006886	intracellular protein transport	U, O	1	1	1	1	1
GO:0009536	plastid	M	1	1	1	1	1
GO:0016863	intramolecular oxidoreductase activity, transposing C=C bonds	C	1	1	1	1	1
GO:0046558	arabinan endo-1,5-alpha-L-arabinosidase activity	G	1	1	1	1	1
GO:0010629	negative regulation of gene expression	K	1	1	1	1	1
GO:0042440	pigment metabolic process	H	1	1	1	1	1
GO:1990837	sequence-specific double-stranded DNA binding	B	1	1	1	1	1
GO:0000038	very long-chain fatty acid metabolic process	I	1	1	1	1	1
GO:0031907	microbody lumen	M	1	1	1	1	1
GO:0009152	purine ribonucleotide biosynthetic process	F, H, C	1	1	1	1	1
GO:0044297	cell body	R	1	1	1	1	1
GO:0022402	cell cycle process	D	1	1	1	1	1
GO:0034660	ncRNA metabolic process	A	1	1	1	1	1
GO:0044540	L-cystine L-cysteine-lyase (deaminating)	C	1	1	1	1	1
GO:0006172	ADP biosynthetic process	F, H, C	1	1	1	1	1
GO:0016812	hydrolase activity, acting on carbon-nitrogen (but not peptide) bonds, in cyclic amides	C	1	1	1	1	1
GO:0046995	oxidoreductase activity, acting on hydrogen as donor, with other known acceptors	C	1	1	1	1	1
GO:0043574	peroxisomal transport	U	1	1	1	1	1
GO:0005488	binding	R	1	1	1	1	1
GO:0009719	response to endogenous stimulus	T	1	1	1	1	1
GO:0015440	ABC-type peptide transporter activity	U	1	1	1	1	1
GO:0043170	macromolecule metabolic process	C	1	1	1	1	1
GO:0006790	sulfur compound metabolic process	C	1	1	1	1	1
GO:0016769	transferase activity, transferring nitrogenous groups	R	1	1	1	1	1
GO:0043750	phosphatidylinositol alpha-mannosyltransferase activity	G	1	1	1	1	1
GO:0140101	catalytic activity, acting on a tRNA	J	1	1	1	1	1
GO:0031012	extracellular matrix	W	1	1	1	1	1
GO:0046395	carboxylic acid catabolic process	H	1	1	1	1	1
GO:0090407	organophosphate biosynthetic process	C	1	1	1	1	1
GO:1903506	regulation of nucleic acid-templated transcription	R	1	1	1	1	1
GO:0043130	ubiquitin binding	O	1	1	1	1	1
GO:0022836	gated channel activity	U	1	1	1	1	1
GO:0016638	oxidoreductase activity, acting on the CH-NH2 group of donors	E	1	1	1	1	1
GO:0015574	trehalose transmembrane transporter activity	G	1	1	1	1	1
GO:0051239	regulation of multicellular organismal process	T	1	1	1	1	1
GO:0051171	regulation of nitrogen compound metabolic process	T, C	1	1	1	1	1
GO:0047810	D-alanine:2-oxoglutarate aminotransferase activity	E	1	1	1	1	1
GO:0005215	transporter activity	R	1	1	1	1	1
GO:0042646	plastid nucleoid	J	1	1	1	1	1
GO:0060567	negative regulation of termination of DNA-templated transcription	K, O	1	1	1	1	1
GO:0005777	peroxisome	M	1	1	1	1	1
GO:0140097	catalytic activity, acting on DNA	B	1	1	1	1	1
GO:0016405	CoA-ligase activity	H	1	1	1	1	1
GO:0016829	lyase activity	C	1	1	1	1	1
GO:0016094	polyprenol biosynthetic process	Q	1	1	1	1	1
GO:0016407	acetyltransferase activity	I	1	1	1	1	1
GO:0019323	pentose catabolic process	H, G	1	1	1	1	1
GO:0140313	molecular sequestering activity	T	1	1	1	1	1
GO:0090613	5'-deoxyadenosine deaminase activity	C	1	1	1	1	1
GO:0045229	external encapsulating structure organization	W	1	1	1	1	1
GO:0009110	vitamin biosynthetic process	H	1	1	1	1	1
GO:0015154	disaccharide transmembrane transporter activity	G	1	1	1	1	1
GO:0032838	plasma membrane bounded cell projection cytoplasm	M	1	1	1	1	1
GO:0000940	outer kinetochore	Z, O	1	1	1	1	1
GO:0000123	histone acetyltransferase complex	B	1	1	1	1	1
GO:0000793	condensed chromosome	B	1	1	1	1	1
GO:0008299	isoprenoid biosynthetic process	Q	1	1	1	1	1
GO:0015695	organic cation transport	R	1	1	1	1	1
GO:0006412	translation	J	1	1	1	1	1
GO:0043603	amide metabolic process	C	1	1	1	1	1
GO:0001653	peptide receptor activity	T	1	1	1	1	1
GO:0006633	fatty acid biosynthetic process	I	1	1	1	1	1
GO:0015748	organophosphate ester transport	R	1	1	1	1	1
GO:0042910	xenobiotic transmembrane transporter activity	Q, U	1	1	1	1	1
GO:0140678	molecular function inhibitor activity	T	1	1	1	1	1
GO:0043207	response to external biotic stimulus	T	1	1	1	1	1
GO:0016887			1	1	1	1	1
GO:0005740	mitochondrial envelope	M	1	1	1	1	1
GO:1902494	catalytic complex	O	1	1	1	1	1
GO:0022624	proteasome accessory complex	O	1	1	1	1	1
GO:0005782	peroxisomal matrix	M	1	1	1	1	1
GO:0016846	carbon-sulfur lyase activity	C	1	1	1	1	1

GO_term	GO_term_name	COG_LETTER	*N. niacini*	*N. driksii* strains			
			DSM 2923T	179-C4-2-HS	.79-J 1A1 H:	AT2.8	V4I25
GO:0006022	aminoglycan metabolic process	G	1	1	1	1	1
GO:0019842	vitamin binding	H	1	1	1	1	1
GO:0042575	DNA polymerase complex	O	1	1	1	1	1
GO:0012505	endomembrane system	M, U	1	1	1	1	1
GO:1990234	transferase complex	O	1	1	1	1	1
GO:0006631	fatty acid metabolic process	I	1	1	1	1	1
GO:0009698	phenylpropanoid metabolic process	Q	1	1	1	1	1
GO:0048518	positive regulation of biological process	T	1	1	1	1	1
GO:0070727	cellular macromolecule localization	U	1	1	1	1	1
GO:0005732	sno(s)RNA-containing ribonucleoprotein complex	O	1	1	1	1	1
GO:0031667	response to nutrient levels	T	1	1	1	1	1
GO:0071692	protein localization to extracellular region	O	1	1	1	1	1
GO:0065007	biological regulation	T	1	1	1	1	1
GO:0008233	peptidase activity	O	1	1	1	1	1
GO:0033764	steroid dehydrogenase activity, acting on the CH-OH group of donors, NAD or NADP as acceptor	Q	1	1	1	1	1
GO:0050182	phosphate butyryltransferase activity	I	1	1	1	1	1
GO:0004672	protein kinase activity	O	1	1	1	1	1
GO:0030203	glycosaminoglycan metabolic process	G	1	1	1	1	1
GO:0044093	positive regulation of molecular function	T	1	1	1	1	1
GO:0004410	homocitrate synthase activity	C	1	1	1	1	1
GO:0008483	transaminase activity	R	1	1	1	1	1
GO:0051716	cellular response to stimulus	T	1	1	1	1	1
GO:0015631	tubulin binding	Z	1	1	1	1	1
GO:0043086	negative regulation of catalytic activity	T	1	1	1	1	1
GO:0016780	phosphotransferase activity, for other substituted phosphate groups	P, T	1	1	1	1	1
GO:0005886	plasma membrane	M	1	1	1	1	1
GO:0070566	adenylyltransferase activity	P, T	1	1	1	1	1
GO:0016862	intramolecular oxidoreductase activity, interconverting keto- and enol-groups	C	1	1	1	1	1
GO:0015749	monosaccharide transmembrane transport	G	1	1	1	1	1
GO:0034010	sulfolactate sulfo-lyase activity	C	1	1	1	1	1
GO:0016614	oxidoreductase activity, acting on CH-OH group of donors	C	1	1	1	1	1
GO:0033920	6-phospho-beta-galactosidase activity	G	1	1	1	1	1
GO:0015645	fatty acid ligase activity	C	1	1	1	1	1
GO:0050308	sugar-phosphatase activity	C	1	1	1	1	1
GO:0004536	deoxyribonuclease activity	B	1	1	1	1	1
GO:0005452	solute:inorganic anion antiporter activity	P	1	1	1	1	1
GO:0032984	protein-containing complex disassembly	O	1	1	1	1	1
GO:0044550	secondary metabolite biosynthetic process	Q	1	1	1	1	1
GO:0009083	branched-chain amino acid catabolic process	E	1	1	1	1	1
GO:0016880	acid-ammonia (or amide) ligase activity	C	1	1	1	1	1
GO:0015772	oligosaccharide transport	G	1	1	1	1	1
GO:0044283	small molecule biosynthetic process	H	1	1	1	1	1
GO:0016832	aldehyde-lyase activity	C	1	1	1	1	1
GO:0016053	organic acid biosynthetic process	H	1	1	1	1	1
GO:0023051	regulation of signaling	T	1	1	1	1	1
GO:0032392	DNA geometric change	B	1	1	1	1	1
GO:0016746	acyltransferase activity	I	1	1	1	1	1
GO:0035091	phosphatidylinositol binding	I	1	1	1	1	1
GO:0042364	water-soluble vitamin biosynthetic process	H	1	1	1	1	1
GO:0033674	positive regulation of kinase activity	P, T	1	1	1	1	1
GO:0009235	cobalamin metabolic process	H	1	1	1	1	1
GO:0031326	regulation of cellular biosynthetic process	T, C	1	1	1	1	1
GO:0043043	peptide biosynthetic process	E, Q	1	1	1	1	1
GO:0023052	signaling	T	1	1	1	1	1
GO:0006625	protein targeting to peroxisome	O	1	1	1	1	1
GO:0015503	glutathione-regulated potassium exporter activity	P, U	1	1	1	1	1
GO:0045881	positive regulation of sporulation resulting in formation of a cellular spore	D	1	1	1	1	1
GO:0036211	protein modification process	O	1	1	1	1	1
GO:0008168	methyltransferase activity	R	1	1	1	1	1
GO:0000313	organellar ribosome	J	1	1	1	1	1
GO:0047681	aryl-alcohol dehydrogenase (NADP+) activity	C	1	1	1	1	1
GO:0042592	homeostatic process	R	1	1	1	1	1
GO:0031248	protein acetyltransferase complex	O	1	1	1	1	1
GO:0009758	carbohydrate utilization	G	1	1	1	1	1
GO:0005635	nuclear envelope	Y	1	1	1	1	1
GO:0001932	regulation of protein phosphorylation	O	1	1	1	1	1
GO:0008673	2-dehydro-3-deoxygluconokinase activity	P, T	1	1	1	1	1
GO:0016093	polyprenol metabolic process	H	1	1	1	1	1
GO:0006281	DNA repair	L	1	1	1	1	1
GO:0001669	acrosomal vesicle	M, U	1	1	1	1	1
GO:0008980	propionate kinase activity	P, T	1	1	1	1	1
GO:0019153	protein-disulfide reductase (glutathione) activity	O	1	1	1	1	1
GO:0046459	short-chain fatty acid metabolic process	I	1	1	1	1	1
GO:0015929	hexosaminidase activity	G	1	1	1	1	1

GO_term	GO_term_name	COG_LETTER	*N. niacini*	*N. driksii* strains			
			DSM 2923T	179-C4-2-HS	.79-J 1A1 H:	AT2.8	V4I25
GO:0065008	regulation of biological quality	T	1	1	1	1	1
GO:0016773	phosphotransferase activity, alcohol group as acceptor	P, T	1	1	1	1	1
GO:0099128	mitochondrial iron-sulfur cluster assembly complex	O	1	1	1	1	1
GO:0016877	ligase activity, forming carbon-sulfur bonds	R	1	1	1	1	1
GO:0015031	protein transport	O	1	1	1	1	1
GO:0140535	intracellular protein-containing complex	O	1	1	1	1	1
GO:0032787	monocarboxylic acid metabolic process	H	1	1	1	1	1
GO:0004563	beta-N-acetylhexosaminidase activity	G	1	1	1	1	1
GO:0044419	biological process involved in interspecies interaction between organisms	T	1	1	1	1	1
GO:0008738	L-fuculose-phosphate aldolase activity	C	1	1	1	1	1
GO:0043233	organelle lumen	M	1	1	1	1	1
GO:0016758	hexosyltransferase activity	G	1	1	1	1	1
GO:0098797	plasma membrane protein complex	M	1	1	1	1	1
GO:0031981	nuclear lumen	Y	1	1	1	1	1
GO:0004046	aminoacylase activity	C	1	1	1	1	1
GO:0010604	positive regulation of macromolecule metabolic process	C	1	1	1	1	1
GO:0009628	response to abiotic stimulus	T	1	1	1	1	1
GO:0031224			1	1	1	1	1
GO:0015103	inorganic anion transmembrane transporter activity	P	1	1	1	1	1
GO:1903825	organic acid transmembrane transport	C	1	1	1	1	1
GO:1901135	carbohydrate derivative metabolic process	G	1	1	1	1	1
GO:0017076	purine nucleotide binding	F	1	1	1	1	1
GO:0008652	amino acid biosynthetic process	H	1	1	1	1	1
GO:0031314	extrinsic component of mitochondrial inner membrane	M	1	1	1	1	1
GO:0048731	system development	D	1	1	1	1	1
GO:0071702	organic substance transport	R	1	1	1	1	1
GO:0009532	plastid stroma	M	1	1	1	1	1
GO:0007017	microtubule-based process	Z	1	1	1	1	1
GO:0098687	chromosomal region	B	1	1	1	1	1
GO:0045184	establishment of protein localization	O	1	1	1	1	1
GO:0031083	BLOC-1 complex	O	1	1	1	1	1
GO:0055085	transmembrane transport	R	1	1	1	1	1
GO:0033907	beta-D-fucosidase activity	G	1	1	1	1	1
GO:0045277	respiratory chain complex IV	M, O	1	1	1	1	1
GO:0006290	pyrimidine dimer repair	L	1	1	1	1	1
GO:0015882	L-ascorbic acid transmembrane transport	H	1	1	1	1	1
GO:0005762	mitochondrial large ribosomal subunit	O	1	1	1	1	1
GO:0009135	purine nucleoside diphosphate metabolic process	H	1	1	1	1	1
GO:0051234	establishment of localization	R	1	1	1	1	1
GO:0006353	DNA-templated transcription termination	K	1	1	1	1	1
GO:0120241	2-iminobutanoate/2-iminopropanoate deaminase	C	1	1	1	1	1
GO:0051649	establishment of localization in cell	U	1	1	1	1	1
GO:0004754	saccharopine dehydrogenase (NAD+, L-lysine-forming) activity	C	1	1	1	1	1
GO:0009888	tissue development	D	1	1	1	1	1
GO:0008470	isovaleryl-CoA dehydrogenase activity	H	1	1	1	1	1
GO:0031967	organelle envelope	M	1	1	1	1	1
GO:1901360	organic cyclic compound metabolic process	C	1	1	1	1	1
GO:0008237	metallopeptidase activity	O	1	1	1	1	1
GO:0047044	androstan-3-alpha,17-beta-diol dehydrogenase activity	Q	1	1	1	1	1
GO:0009068	aspartate family amino acid catabolic process	E	1	1	1	1	1
GO:0050789	regulation of biological process	T	1	1	1	1	1
GO:0015925	galactosidase activity	G	1	1	1	1	1
GO:0043211	ABC-type carbohydrate transporter activity	G	1	1	1	1	1
GO:0034212	peptide N-acetyltransferase activity	I	1	1	1	1	1
GO:0005938	cell cortex	W	1	1	1	1	1
GO:0140299	small molecule sensor activity	T	1	1	1	1	1
GO:0016628	oxidoreductase activity, acting on the CH-CH group of donors, NAD or NADP as acceptor	C	1	1	1	1	1
GO:0016717	oxidoreductase activity, acting on paired donors, with oxidation of a pair of donors resulting in the reduction of molecular oxygen to two molecules of water	Q	1	1	1	1	1
GO:0051539			1	1	1	1	1
GO:0008865	fructokinase activity	P, T	1	1	1	1	1
GO:0047868	dimethylmaleate hydratase activity	C	1	1	1	1	1
GO:0004930	G protein-coupled receptor activity	T	1	1	1	1	1
GO:0031401	positive regulation of protein modification process	O	1	1	1	1	1
GO:0006807	nitrogen compound metabolic process	C	1	1	1	1	1
GO:0010556	regulation of macromolecule biosynthetic process	T, C	1	1	1	1	1
GO:0051701	biological process involved in interaction with host	T	1	1	1	1	1
GO:0043296	apical junction complex	W	1	1	1	1	1
GO:0047794	cyclohexadienyl dehydrogenase activity	C	1	1	1	1	1
GO:0009057	macromolecule catabolic process	C	1	1	1	1	1
GO:0006721	terpenoid metabolic process	I	1	1	1	1	1
GO:0005794	Golgi apparatus	M, U	1	1	1	1	1
GO:0016783	sulfurtransferase activity	P	1	1	1	0	1
GO:0006637	acyl-CoA metabolic process	H	1	1	1	0	1
GO:0070568	guanylyltransferase activity	P, T	1	1	1	0	1

GO_term	GO_term_name	COG_LETTER	*N. niacini*	*N. driksii* strains			
			DSM 2923T	179-C4-2-HS	.79-J 1A1 H:	AT2.8	V4I25
GO:0016784	3-mercaptopyruvate sulfurtransferase activity	P	1	1	1	0	1
GO:0072359	circulatory system development	D	1	1	1	0	1
GO:0009100	glycoprotein metabolic process	G, O	1	1	1	0	1
GO:0070160	tight junction	W	1	1	1	0	1
GO:0061024	membrane organization	M	1	1	1	0	1
GO:0008928	mannose-1-phosphate guanylyltransferase (GDP) activity	P, T	1	1	1	0	1
GO:0033705	GDP-4-dehydro-6-deoxy-D-mannose reductase activity	C	1	1	1	0	1
GO:0009894	regulation of catabolic process	T, C	1	1	1	0	1
GO:0035383	thioester metabolic process	C	1	1	1	0	1
GO:0001503	ossification	R	1	1	1	0	1
GO:1902555	endoribonuclease complex	O	1	1	0	1	1
GO:0031226			1	1	0	1	1
GO:0042277	peptide binding	R	1	1	0	1	1
GO:0005887			1	1	0	1	1
GO:0009266	response to temperature stimulus	T	1	1	0	1	1
GO:0009806	lignan metabolic process	Q	1	1	0	1	1
GO:0033293	monocarboxylic acid binding	R	1	1	0	1	1
GO:0019005	SCF ubiquitin ligase complex	O	1	1	0	1	1
GO:0016125	sterol metabolic process	I	1	1	0	1	1
GO:0016072	rRNA metabolic process	A	1	1	0	1	1
GO:0019362	pyridine nucleotide metabolic process	F	1	1	0	1	1
GO:0034068	aminoglycoside nucleotidyltransferase activity	P, T	1	1	0	1	1
GO:0046496	nicotinamide nucleotide metabolic process	H	1	1	0	1	1
GO:0004060	arylamine N-acetyltransferase activity	I	1	1	0	1	1
GO:0006739	NADP metabolic process	H	1	1	0	1	1
GO:0009012	aminoglycoside 3''-adenylyltransferase activity	P, T	1	1	0	1	1
GO:0009807	lignan biosynthetic process	Q, C	1	1	0	1	1
GO:0072593	reactive oxygen species metabolic process	C	1	0	1	1	1
GO:0048609	multicellular organismal reproductive process	D	1	0	1	1	1
GO:0032504	multicellular organism reproduction	D	1	0	1	1	1
GO:0044097	secretion by the type IV secretion system	U	1	0	1	1	1
GO:0022843	voltage-gated monoatomic cation channel activity	P	1	0	1	1	1
GO:0007276	gamete generation	D	1	0	1	1	1
GO:0045127	N-acetylglucosamine kinase activity	P, T, G	1	0	1	1	1
GO:0030255	protein secretion by the type IV secretion system	O	1	0	1	1	1
GO:0005216	monoatomic ion channel activity	U	1	0	1	1	1
GO:0017007	protein-bilin linkage	O	1	0	1	1	1
GO:0017006	protein-tetrapyrrole linkage	O	1	0	1	1	1
GO:0017009	protein-phycocyanobilin linkage	O	1	0	1	1	1
GO:0030506	ankyrin binding	Z	1	0	1	1	1
GO:0009534	chloroplast thylakoid	C	0	1	1	1	0
GO:0099568	cytoplasmic region	R	0	1	1	1	0
GO:0150034	distal axon	M	0	1	1	1	0
GO:0072527	pyrimidine-containing compound metabolic process	C	0	1	1	1	0
GO:0008094	ATP-dependent activity, acting on DNA	B	0	1	1	1	0
GO:0043190			0	1	1	1	0
GO:0031047	RNA-mediated gene silencing	K	0	1	1	1	0
GO:0045171	intercellular bridge	R	0	1	1	1	0
GO:0042304	regulation of fatty acid biosynthetic process	I	0	1	1	1	0
GO:0006111	regulation of gluconeogenesis	H, G	0	1	1	1	0
GO:0015807	L-amino acid transport	E	0	1	1	1	0
GO:0062197	cellular response to chemical stress	T	0	1	1	1	0
GO:0046058	cAMP metabolic process	F	0	1	1	1	0
GO:1990351	transporter complex	O	0	1	1	1	0
GO:0016137	glycoside metabolic process	G	0	1	1	1	0
GO:0019217	regulation of fatty acid metabolic process	I	0	1	1	1	0
GO:0009579	thylakoid	C	0	1	1	1	0
GO:0051478	mannosylglycerate metabolic process	G	0	1	1	1	0
GO:0003939	L-iditol 2-dehydrogenase activity	C	0	1	1	1	0
GO:0047760	butyrate-CoA ligase activity	H, C	0	1	1	1	0
GO:0009162	deoxyribonucleoside monophosphate metabolic process	H	0	1	1	1	0
GO:0009089	lysine biosynthetic process via diaminopimelate	E	0	1	1	1	0
GO:0009125	nucleoside monophosphate catabolic process	H	0	1	1	1	0
GO:0045721	negative regulation of gluconeogenesis	H, G	0	1	1	1	0
GO:0007623	circadian rhythm	T	0	1	1	1	0
GO:0045111	intermediate filament cytoskeleton	Z	0	1	1	1	0
GO:0005882	intermediate filament	Z	0	1	1	1	0
GO:0003697	single-stranded DNA binding	B	0	1	1	1	0
GO:0051235	maintenance of location	R	0	1	1	1	0
GO:0046467	membrane lipid biosynthetic process	I	0	1	1	1	0
GO:0009064	glutamine family amino acid metabolic process	E	0	1	1	1	0
GO:0044877	protein-containing complex binding	O	0	1	1	1	0
GO:1902495	transmembrane transporter complex	M, O	0	1	1	1	0
GO:0009261	ribonucleotide catabolic process	F, G	0	1	1	1	0
GO:0019156	isoamylase activity	G	0	1	1	1	0
GO:0033647	host intracellular organelle	R	0	1	1	1	0
GO:0016795	phosphoric triester hydrolase activity	C	0	1	1	1	0
GO:0090079			0	1	1	1	0

GO_term	GO_term_name	COG_LETTER	*N. niacini*	*N. driksii* strains		
			DSM 2923T	179-C4-2-HS	.79-J 1A1 H	AT2.8	V4I25
GO:0003955	NAD(P)H dehydrogenase (quinone) activity	C	0	1	1	1	0
GO:0009082			0	1	1	1	0
GO:0000026	alpha-1,2-mannosyltransferase activity	G	0	1	1	1	0
GO:0009739	response to gibberellin	T	0	1	1	1	0
GO:0005275	amine transmembrane transporter activity	U	0	1	1	1	0
GO:0046555	acetylxylan esterase activity	C	0	1	1	1	0
GO:0008375	acetylglucosaminyltransferase activity	G	0	1	1	1	0
GO:0004063	aryldialkylphosphatase activity	C	0	1	1	1	0
GO:0006260	DNA replication	B, L	0	1	1	1	0
GO:0042537	benzene-containing compound metabolic process	C	0	1	1	1	0
GO:0019843	rRNA binding	A	0	1	1	1	0
GO:0031668	cellular response to extracellular stimulus	T	0	1	1	1	0
GO:0019210	kinase inhibitor activity	T	0	1	1	1	0
GO:0006873	intracellular monoatomic ion homeostasis	R	0	1	1	1	0
GO:0004090	carbonyl reductase (NADPH) activity	C	0	1	1	1	0
GO:0042182	ketone catabolic process	H	0	1	1	1	0
GO:0033854	glutamate-prephenate aminotransferase activity	E	0	1	1	1	0
GO:0015939	pantothenate metabolic process	H	0	1	1	1	0
GO:0030964	NADH dehydrogenase complex	M, O	0	1	1	1	0
GO:0032561	guanyl ribonucleotide binding	F, G	0	1	1	1	0
GO:0006783			0	1	1	1	0
GO:0032958	inositol phosphate biosynthetic process	H	0	1	1	1	0
GO:0015556	C4-dicarboxylate transmembrane transporter activity	U, C	0	1	1	1	0
GO:0005049	nuclear export signal receptor activity	U	0	1	1	1	0
GO:0019249	lactate biosynthetic process	H, C	0	1	1	1	0
GO:0017056	structural constituent of nuclear pore	Y, U, O	0	1	1	1	0
GO:0016651	oxidoreductase activity, acting on NAD(P)H	C	0	1	1	1	0
GO:0009127	purine nucleoside monophosphate biosynthetic process	H, C	0	1	1	1	0
GO:0003333	amino acid transmembrane transport	E	0	1	1	1	0
GO:0005496	steroid binding	I	0	1	1	1	0
GO:0042086	5-methyl-5,6,7,8-tetrahydromethanopterin-dependent methyltransferase activity	C	0	1	1	1	0
GO:0018667	cyclohexanone monooxygenase activity	H	0	1	1	1	0
GO:0003924			0	1	1	1	0
GO:0015940			0	1	1	1	0
GO:0015081	sodium ion transmembrane transporter activity	P	0	1	1	1	0
GO:1901618	organic hydroxy compound transmembrane transporter activity	U	0	1	1	1	0
GO:0017174	glycine N-methyltransferase activity	H	0	1	1	1	0
GO:0006189			0	1	1	1	0
GO:0043414	macromolecule methylation	C	0	1	1	1	0
GO:0031253	cell projection membrane	M	0	1	1	1	0
GO:0015038	glutathione disulfide oxidoreductase activity	C	0	1	1	1	0
GO:1902074	response to salt	P	0	1	1	1	0
GO:0034311	diol metabolic process	H	0	1	1	1	0
GO:0047487	oligogalacturonide lyase activity	G	0	1	1	1	0
GO:0050136			0	1	1	1	0
GO:0008654	phospholipid biosynthetic process	I	0	1	1	1	0
GO:0009156	ribonucleoside monophosphate biosynthetic process	H, C	0	1	1	1	0
GO:0000096	sulfur amino acid metabolic process	C	0	1	1	1	0
GO:0009085	lysine biosynthetic process	E	0	1	1	1	0
GO:0007005	mitochondrion organization	R	0	1	1	1	0
GO:0010427	abscisic acid binding	Q, C	0	1	1	1	0
GO:0033692	cellular polysaccharide biosynthetic process	R	0	1	1	1	0
GO:0061929	gamma-glutamylaminecyclotransferase activity	C	0	1	1	1	0
GO:0016469	proton-transporting two-sector ATPase complex	C	0	1	1	1	0
GO:0035725	sodium ion transmembrane transport	P	0	1	1	1	0
GO:0046415	urate metabolic process	H	0	1	1	1	0
GO:0005912	adherens junction	W	0	1	1	1	0
GO:0044217	other organism part	R	0	1	1	1	0
GO:0045185	maintenance of protein location	O	0	1	1	1	0
GO:0045017	glycerolipid biosynthetic process	I	0	1	1	1	0
GO:0036376	sodium ion export across plasma membrane	U	0	1	1	1	0
GO:0047663	aminoglycoside 6'-N-acetyltransferase activity	I	0	1	1	1	0
GO:0031365	N-terminal protein amino acid modification	O	0	1	1	1	0
GO:0046173	polyol biosynthetic process	H	0	1	1	1	0
GO:0001883	purine nucleoside binding	F	0	1	1	1	0
GO:0070062	extracellular exosome	W	0	1	1	1	0
GO:0008213	protein alkylation	O	0	1	1	1	0
GO:0072329	monocarboxylic acid catabolic process	H	0	1	1	1	0
GO:0018493	formylmethanofuran dehydrogenase activity	C	0	1	1	1	0
GO:0050446	azobenzene reductase activity	C	0	1	1	1	0
GO:0050520	phosphatidylcholine synthase activity	P, T	0	1	1	1	0
GO:0004377	GDP-Man:Man3GlcNAc2-PP-Dol alpha-1,2-mannosyltransferase activity	G	0	1	1	1	0
GO:0006479	protein methylation	O	0	1	1	1	0
GO:0017116	single-stranded DNA helicase activity	B	0	1	1	1	0
GO:0016803	ether hydrolase activity	C	0	1	1	1	0
GO:0048367	shoot system development	D	0	1	1	1	0

GO_term	GO_term_name	COG_LETTER	*N. niacini*	*N. driksii* strains			
			DSM 2923T	179-C4-2-HS	.79-J 1A1 H:	AT2.8	V4I25
GO:0019482	beta-alanine metabolic process	E	0	1	1	1	0
GO:0033648	host intracellular membrane-bounded organelle	R	0	1	1	1	0
GO:0005982	starch metabolic process	G	0	1	1	1	0
GO:0062012	regulation of small molecule metabolic process	H	0	1	1	1	0
GO:0046040	IMP metabolic process	F	0	1	1	1	0
GO:0140115	export across plasma membrane	U	0	1	1	1	0
GO:0004809	tRNA (guanine-N2-)-methyltransferase activity	R	0	1	1	1	0
GO:0010675	regulation of cellular carbohydrate metabolic process	R	0	1	1	1	0
GO:0042727			0	1	1	1	0
GO:0043650	dicarboxylic acid biosynthetic process	H	0	1	1	1	0
GO:0015780	nucleotide-sugar transmembrane transport	F	0	1	1	1	0
GO:1901657	glycosyl compound metabolic process	G	0	1	1	1	0
GO:0032045	guanyl-nucleotide exchange factor complex	O	0	1	1	1	0
GO:1901616	organic hydroxy compound catabolic process	C	0	1	1	1	0
GO:0005525			0	1	1	1	0
GO:0070180			0	1	1	1	0
GO:0018671	4-hydroxybenzoate 3-monooxygenase [NAD(P)H] activity	H	0	1	1	1	0
GO:0019400	alditol metabolic process	G	0	1	1	1	0
GO:0044265	cellular macromolecule catabolic process	R	0	1	1	1	0
GO:0015651	quaternary ammonium group transmembrane transporter activity	U	0	1	1	1	0
GO:0036477	somatodendritic compartment	R	0	1	1	1	0
GO:0000030	mannosyltransferase activity	G	0	1	1	1	0
GO:0009432			0	1	1	1	0
GO:0010476	gibberellin mediated signaling pathway	T	0	1	1	1	0
GO:0098858	actin-based cell projection	M, Z	0	1	1	1	0
GO:0070972	protein localization to endoplasmic reticulum	O	0	1	1	1	0
GO:0044770	cell cycle phase transition	D	0	1	1	1	0
GO:0046451	diaminopimelate metabolic process	H	0	1	1	1	0
GO:0072545	tyrosine binding	E	0	1	1	1	0
GO:0009168	purine ribonucleoside monophosphate biosynthetic process	H, C	0	1	1	1	0
GO:0050281	serine-glyoxylate transaminase activity	E	0	1	1	1	0
GO:0009161	ribonucleoside monophosphate metabolic process	H	0	1	1	1	0
GO:0046890	regulation of lipid biosynthetic process	I	0	1	1	1	0
GO:0047635	alanine-oxo-acid transaminase activity	E	0	1	1	1	0
GO:0047809	D-2-hydroxy-acid dehydrogenase activity	C	0	1	1	1	0
GO:0034069	aminoglycoside N-acetyltransferase activity	I	0	1	1	1	0
GO:0010011	auxin binding	Q	0	1	1	1	0
GO:0031414	N-terminal protein acetyltransferase complex	O	0	1	1	1	0
GO:0046522	S-methyl-5-thioribose kinase activity	P, T	0	1	1	1	0
GO:0032101	regulation of response to external stimulus	T	0	1	1	1	0
GO:0005793	endoplasmic reticulum-Golgi intermediate compartment	M	0	1	1	1	0
GO:0006835	dicarboxylic acid transport	C	0	1	1	1	0
GO:0016655	oxidoreductase activity, acting on NAD(P)H, quinone or similar compound as acceptor	C	0	1	1	1	0
GO:0043684	type IV secretion system complex	O	0	1	1	1	0
GO:0016403	dimethylargininase activity	C	0	1	1	1	0
GO:0006665	sphingolipid metabolic process	I, C	0	1	1	1	0
GO:0045833	negative regulation of lipid metabolic process	I	0	1	1	1	0
GO:0016801	hydrolase activity, acting on ether bonds	C	0	1	1	1	0
GO:0044218	other organism cell membrane	R	0	1	1	1	0
GO:0003724	RNA helicase activity	A	0	1	1	1	0
GO:0050368	tyrosine 2,3-aminomutase activity	E	0	1	1	1	0
GO:0008911	lactaldehyde dehydrogenase activity	C	0	1	1	1	0
GO:0047584	4-oxalmesaconate hydratase activity	C	0	1	1	1	0
GO:0055080	monoatomic cation homeostasis	R	0	1	1	1	0
GO:0031528	microvillus membrane	M	0	1	1	1	0
GO:0009514	glyoxysome	M	0	1	1	1	0
GO:0016840	carbon-nitrogen lyase activity	C	0	1	1	1	0
GO:0002684	positive regulation of immune system process	W	0	1	1	1	0
GO:0009116	nucleoside metabolic process	H	0	1	1	1	0
GO:0035869	ciliary transition zone	M, O	0	1	1	1	0
GO:0006096	glycolytic process	H, C	0	1	1	1	0
GO:0061134	peptidase regulator activity	T, O	0	1	1	1	0
GO:0042398	cellular modified amino acid biosynthetic process	C	0	1	1	1	0
GO:0072595	maintenance of protein localization in organelle	U, O	0	1	1	1	0
GO:0006555	methionine metabolic process	E	0	1	1	1	0
GO:0032507	maintenance of protein location in cell	U	0	1	1	1	0
GO:0019953	sexual reproduction	D	0	1	1	1	0
GO:0008172	S-methyltransferase activity	C	0	1	1	1	0
GO:0009126	purine nucleoside monophosphate metabolic process	H	0	1	1	1	0
GO:0072523	purine-containing compound catabolic process	C	0	1	1	1	0
GO:0047540	2-enoate reductase activity	C	0	1	1	1	0
GO:0071496	cellular response to external stimulus	T	0	1	1	1	0
GO:0016837	carbon-oxygen lyase activity, acting on polysaccharides	G	0	1	1	1	0
GO:0017050	D-erythro-sphingosine kinase activity	P, T	0	1	1	1	0
GO:0006757	ATP generation from ADP	R	0	1	1	1	0
GO:0016427	tRNA (cytosine) methyltransferase activity	H	0	1	1	1	0
GO:0008137			0	1	1	1	0

GO_term	GO_term_name	COG_LETTER	*N. niacini*	*N. driksii* strains			
			DSM 2923T	179-C4-2-HS	.79-J 1A1 H:	AT2.8	V4I25
GO:0032550			0	1	1	1	0
GO:0000075	cell cycle checkpoint signaling	T	0	1	1	1	0
GO:0098732	macromolecule deacylation	C	0	1	1	1	0
GO:0009159	deoxyribonucleoside monophosphate catabolic process	H	0	1	1	1	0
GO:0070647	protein modification by small protein conjugation or removal	O	0	1	1	1	0
GO:0009154	purine ribonucleotide catabolic process	F, G	0	1	1	1	0
GO:0004175	endopeptidase activity	O	0	1	1	1	0
GO:0050081	maltose-6'-phosphate glucosidase activity	G	0	1	1	1	0
GO:0009098			0	1	1	1	0
GO:1990814	DNA/DNA annealing activity	B	0	1	1	1	0
GO:0016971	flavin-linked sulfhydryl oxidase activity	O	0	1	1	1	0
GO:0065010	extracellular membrane-bounded organelle	M, W	0	1	1	1	0
GO:0047417	N-carbamoyl-D-amino acid hydrolase activity	C	0	1	1	1	0
GO:0005345	purine nucleobase transmembrane transporter activity	F, U	0	1	1	1	0
GO:0000097	sulfur amino acid biosynthetic process	H	0	1	1	1	0
GO:0015747	urate transport	G	0	1	1	1	0
GO:0010960	magnesium ion homeostasis	P	0	1	1	1	0
GO:0019829	ATPase-coupled monoatomic cation transmembrane transporter activity	P	0	1	1	1	0
GO:0006771			0	1	1	1	0
GO:0034637	cellular carbohydrate biosynthetic process	R	0	1	1	1	0
GO:0006862	nucleotide transport	F	0	1	1	1	0
GO:0019840	isoprenoid binding	I	0	1	1	1	0
GO:0005911	cell-cell junction	W	0	1	1	1	0
GO:0009086	methionine biosynthetic process	E, H	0	1	1	1	0
GO:0016213	linoleoyl-CoA desaturase activity	Q	0	1	1	1	0
GO:0009214	cyclic nucleotide catabolic process	F	0	1	1	1	0
GO:0030283	testosterone dehydrogenase [NAD(P)] activity	Q	0	1	1	1	0
GO:0006450			0	1	1	1	0
GO:0034704	calcium channel complex	M, O	0	1	1	1	0
GO:0007186	G protein-coupled receptor signaling pathway	T	0	1	1	1	0
GO:0051286	cell tip	R	0	1	1	1	0
GO:0032446	protein modification by small protein conjugation	O	0	1	1	1	0
GO:0006809	nitric oxide biosynthetic process	C	0	1	1	1	0
GO:0002161	aminoacyl-tRNA editing activity	J	0	1	1	1	0
GO:0031505	fungal-type cell wall organization	M	0	1	1	1	0
GO:0046474	glycerophospholipid biosynthetic process	I	0	1	1	1	0
GO:0031976	plastid thylakoid	C	0	1	1	1	0
GO:0015205	nucleobase transmembrane transporter activity	F, U	0	1	1	1	0
GO:0020037			0	1	1	1	0
GO:0051911	Methanosarcina-phenazine hydrogenase activity	C	0	1	1	1	0
GO:0006782			0	1	1	1	0
GO:0031510	SUMO activating enzyme complex	Y, O	0	1	1	1	0
GO:0042133	neurotransmitter metabolic process	R	0	1	1	1	0
GO:0030266	quinate 3-dehydrogenase (NAD+) activity	C	0	1	1	1	0
GO:0009124	nucleoside monophosphate biosynthetic process	H, C	0	1	1	1	0
GO:0008270			0	1	1	1	0
GO:0042844	glycol metabolic process	H	0	1	1	1	0
GO:0033939	xylan alpha-1,2-glucuronosidase activity	G	0	1	1	1	0
GO:0004021	L-alanine:2-oxoglutarate aminotransferase activity	E	0	1	1	1	0
GO:0031965	nuclear membrane	M, Y	0	1	1	1	0
GO:0019003	GDP binding	F, G	0	1	1	1	0
GO:0006195	purine nucleotide catabolic process	F	0	1	1	1	0
GO:0006364	rRNA processing	J	0	1	1	1	0
GO:0005637	nuclear inner membrane	M, Y	0	1	1	1	0
GO:0051596	methylglyoxal catabolic process	H	0	1	1	1	0
GO:0017101	aminoacyl-tRNA synthetase multienzyme complex	O	0	1	1	1	0
GO:0016813	hydrolase activity, acting on carbon-nitrogen (but not peptide) bonds, in linear amidines	C	0	1	1	1	0
GO:1904407	positive regulation of nitric oxide metabolic process	C	0	1	1	1	0
GO:1902475	L-alpha-amino acid transmembrane transport	E	0	1	1	1	0
GO:0000049	tRNA binding	J	0	1	1	1	0
GO:0046573	lactonohydrolase activity	C	0	1	1	1	0
GO:0005967	mitochondrial pyruvate dehydrogenase complex	O	0	1	1	1	0
GO:0009055			0	1	1	1	0
GO:0050297	stizolobate synthase activity	C	0	1	1	1	0
GO:0006650	glycerophospholipid metabolic process	I	0	1	1	1	0
GO:0006261	DNA-templated DNA replication	L	0	1	1	1	0
GO:0006656	phosphatidylcholine biosynthetic process	I, C	0	1	1	1	0
GO:0009414	response to water deprivation	T	0	1	1	1	0
GO:0000166	nucleotide binding	F	0	1	1	1	0
GO:0034077	butanediol metabolic process	H	0	1	1	1	0
GO:0015980	energy derivation by oxidation of organic compounds	C	0	1	1	1	0
GO:0046874	quinolinate metabolic process	C	0	1	1	1	0
GO:0046501			0	1	1	1	0
GO:0051912	CoB--CoM heterodisulfide reductase activity	C	0	1	1	1	0
GO:0031417	NatC complex	O	0	1	1	1	0
GO:0042562	hormone binding	Q	0	1	1	1	0
GO:0010564	regulation of cell cycle process	D	0	1	1	1	0

GO_term	GO_term_name	COG_LETTER	*N. niacini*	*N. driksii* strains			
			DSM 2923T	179-C4-2-HS	.79-J 1A1 H	AT2.8	V4I25
GO:0044264	cellular polysaccharide metabolic process	R	0	1	1	1	0
GO:0030136	clathrin-coated vesicle	U	0	1	1	1	0
GO:0006091	generation of precursor metabolites and energy	C	0	1	1	1	0
GO:0009339	glycolate oxidase complex	O	0	1	1	1	0
GO:0016138	glycoside biosynthetic process	G	0	1	1	1	0
GO:0009438	methylglyoxal metabolic process	H	0	1	1	1	0
GO:0043255	regulation of carbohydrate biosynthetic process	G	0	1	1	1	0
GO:0030001	metal ion transport	P	0	1	1	1	0
GO:1901264	carbohydrate derivative transport	G	0	1	1	1	0
GO:0005504	fatty acid binding	I	0	1	1	1	0
GO:0048878	chemical homeostasis	R	0	1	1	1	0
GO:0046559	alpha-glucuronidase activity	G	0	1	1	1	0
GO:0002376	immune system process	W	0	1	1	1	0
GO:0000403	Y-form DNA binding	B	0	1	1	1	0
GO:0062014	negative regulation of small molecule metabolic process	H	0	1	1	1	0
GO:0005984	disaccharide metabolic process	G	0	1	1	1	0
GO:0070603	SWI/SNF superfamily-type complex	C	0	1	1	1	0
GO:0045182	translation regulator activity	J	0	1	1	1	0
GO:0098771	inorganic ion homeostasis	P	0	1	1	1	0
GO:0002094	polyprenyltransferase activity	I	0	1	1	1	0
GO:0009129	pyrimidine nucleoside monophosphate metabolic process	H	0	1	1	1	0
GO:0030269	tetrahydromethanopterin S-methyltransferase activity	H	0	1	1	1	0
GO:0016571	histone methylation	R	0	1	1	1	0
GO:0052595	aliphatic amine oxidase activity	E	0	1	1	1	0
GO:0006546			0	1	1	1	0
GO:0005976	polysaccharide metabolic process	G	0	1	1	1	0
GO:0019483	beta-alanine biosynthetic process	E	0	1	1	1	0
GO:0035556	intracellular signal transduction	T	0	1	1	1	0
GO:0047560	3-dehydrosphinganine reductase activity	C	0	1	1	1	0
GO:0009740	gibberellic acid mediated signaling pathway	T	0	1	1	1	0
GO:0032549			0	1	1	1	0
GO:1901701	cellular response to oxygen-containing compound	T	0	1	1	1	0
GO:1901659	glycosyl compound biosynthetic process	G	0	1	1	1	0
GO:0000124	SAGA complex	Y, O	0	1	1	1	0
GO:0060187	cell pole	R	0	1	1	1	0
GO:0044272	sulfur compound biosynthetic process	C	0	1	1	1	0
GO:0019295	coenzyme M biosynthetic process	H	0	1	1	1	0
GO:0001917	photoreceptor inner segment	T	0	1	1	1	0
GO:0015926	glucosidase activity	G	0	1	1	1	0
GO:0052794	exo-alpha-(2->3)-sialidase activity	G	0	1	1	1	0
GO:0016884			0	1	1	1	0
GO:0042127	regulation of cell population proliferation	D	0	1	1	1	0
GO:0004104	cholinesterase activity	C	0	1	1	1	0
GO:0046164	alcohol catabolic process	H	0	1	1	1	0
GO:0016114	terpenoid biosynthetic process	Q	0	1	1	1	0
GO:0008872	glucarate dehydratase activity	C	0	1	1	1	0
GO:0045717	negative regulation of fatty acid biosynthetic process	H, I	0	1	1	1	0
GO:0004576	oligosaccharyl transferase activity	G	0	1	1	1	0
GO:1901998	toxin transport	V	0	1	1	1	0
GO:0043639	benzoate catabolic process	C	0	1	1	1	0
GO:0009415	response to water	T	0	1	1	1	0
GO:0071826	ribonucleoprotein complex subunit organization	J	0	1	1	1	0
GO:0071370	cellular response to gibberellin stimulus	T	0	1	1	1	0
GO:0015812	gamma-aminobutyric acid transport	E	0	1	1	1	0
GO:0009071	serine family amino acid catabolic process	E	0	1	1	1	0
GO:0016051	carbohydrate biosynthetic process	G	0	1	1	1	0
GO:0000271	polysaccharide biosynthetic process	G	0	1	1	1	0
GO:0097268	cytoophidium	Z	0	1	1	1	0
GO:0018205	peptidyl-lysine modification	O	0	1	1	1	0
GO:0016997	alpha-sialidase activity	G	0	1	1	1	0
GO:0017171	serine hydrolase activity	O	0	1	1	1	0
GO:0005774	vacuolar membrane	U	0	1	1	1	0
GO:0004376	glycolipid mannosyltransferase activity	G	0	1	1	1	0
GO:0006006	glucose metabolic process	H, G	0	1	1	1	0
GO:0000287			0	1	1	1	0
GO:0016833	oxo-acid-lyase activity	C	0	1	1	1	0
GO:0016925	protein sumoylation	O	0	1	1	1	0
GO:0009187	cyclic nucleotide metabolic process	F	0	1	1	1	0
GO:0047991	hydroxylamine oxidase activity	C	0	1	1	1	0
GO:0004096			0	1	1	1	0
GO:0045027	DNA end binding	B	0	1	1	1	0
GO:0033776	phenylacetone monooxygenase activity	H	0	1	1	1	0
GO:0004596	peptide alpha-N-acetyltransferase activity	I	0	1	1	1	0
GO:0045922	negative regulation of fatty acid metabolic process	H, I	0	1	1	1	0
GO:0140666	annealing activity	F	0	1	1	1	0
GO:0009158	ribonucleoside monophosphate catabolic process	H	0	1	1	1	0
GO:0017118	lipoyltransferase activity	I	0	1	1	1	0
GO:0051082			0	1	1	1	0

GO_term	GO_term_name	COG_LETTER	*N. niacini*	*N. driksii* strains			
			DSM 2923T	179-C4-2-HS	.79-J 1A1 H:	AT2.8	V4I25
GO:0060170	ciliary membrane	M, O	0	1	1	1	0
GO:0016815	hydrolase activity, acting on carbon-nitrogen (but not peptide) bonds, in nitriles	C	0	1	1	1	0
GO:0033993	response to lipid	T	0	1	1	1	0
GO:0016742			0	1	1	1	0
GO:0019417	sulfur oxidation	C	0	1	1	1	0
GO:0016428	tRNA (cytosine-5-)-methyltransferase activity	H	0	1	1	1	0
GO:0015179	L-amino acid transmembrane transporter activity	E	0	1	1	1	0
GO:0019216	regulation of lipid metabolic process	I	0	1	1	1	0
GO:0009084	glutamine family amino acid biosynthetic process	E	0	1	1	1	0
GO:0010677	negative regulation of cellular carbohydrate metabolic process	R	0	1	1	1	0
GO:0006814	sodium ion transport	P	0	1	1	1	0
GO:0018022	peptidyl-lysine methylation	O	0	1	1	1	0
GO:0072529	pyrimidine-containing compound catabolic process	C	0	1	1	1	0
GO:0002682	regulation of immune system process	T, W	0	1	1	1	0
GO:0080164	regulation of nitric oxide metabolic process	T, C	0	1	1	1	0
GO:0008236	serine-type peptidase activity	O	0	1	1	1	0
GO:0009067	aspartate family amino acid biosynthetic process	E	0	1	1	1	0
GO:1902531	regulation of intracellular signal transduction	T	0	1	1	1	0
GO:0009231			0	1	1	1	0
GO:0006553	lysine metabolic process	E	0	1	1	1	0
GO:0016663	oxidoreductase activity, acting on other nitrogenous compounds as donors, oxygen as acceptor	C	0	1	1	1	0
GO:0045202	synapse	W	0	1	1	1	0
GO:0043647	inositol phosphate metabolic process	H, C	0	1	1	1	0
GO:0052652	cyclic purine nucleotide metabolic process	F	0	1	1	1	0
GO:0048584	positive regulation of response to stimulus	T	0	1	1	1	0
GO:0046148	pigment biosynthetic process	H	0	1	1	1	0
GO:0009167	purine ribonucleoside monophosphate metabolic process	H	0	1	1	1	0
GO:0006026	aminoglycan catabolic process	G	0	1	1	1	0
GO:0004490	methylglutaconyl-CoA hydratase activity	C	0	1	1	1	0
GO:0050801	monoatomic ion homeostasis	R	0	1	1	1	0
GO:0006401	RNA catabolic process	A	0	1	1	1	0
GO:0009069	serine family amino acid metabolic process	E	0	1	1	1	0
GO:0005773	vacuole	U	0	1	1	1	0
GO:0000981	DNA-binding transcription factor activity, RNA polymerase II-specific	K	0	1	1	1	0
GO:1901265	nucleoside phosphate binding	F	0	1	1	1	0
GO:0018874	benzoate metabolic process	C	0	1	1	1	0
GO:0016441	post-transcriptional gene silencing	K	0	1	1	1	0
GO:0008174	mRNA methyltransferase activity	H, A	0	1	1	1	0
GO:0051287	NAD binding	F	0	1	1	1	0
GO:0048511	rhythmic process	T	0	1	1	1	0
GO:0030425	dendrite	M	0	1	1	1	0
GO:0010608	post-transcriptional regulation of gene expression	K	0	1	1	1	0
GO:0072525	pyridine-containing compound biosynthetic process	H	0	1	1	1	0
GO:0030313	cell envelope	M	0	1	1	1	0
GO:0006094	gluconeogenesis	H	0	1	1	1	0
GO:0003688			0	1	1	1	0
GO:0007059	chromosome segregation	D	0	1	1	1	0
GO:0016209	antioxidant activity	V	0	1	1	1	0
GO:0008135			0	1	1	1	0
GO:0018467	formaldehyde dehydrogenase activity	C	0	1	1	1	0
GO:0031297	replication fork processing	B, L	0	1	1	1	0
GO:0016570	histone modification	R	0	1	1	1	0
GO:0019001	guanyl nucleotide binding	F	0	1	1	1	0
GO:0031956	medium-chain fatty acid-CoA ligase activity	H	0	1	1	1	0
GO:0046185	aldehyde catabolic process	C	0	1	1	1	0
GO:0005385	zinc ion transmembrane transporter activity	P	0	1	1	1	0
GO:0046565	3-dehydroshikimate dehydratase activity	C	0	1	1	1	0
GO:0006955	immune response	T, W	0	1	1	1	0
GO:0019318	hexose metabolic process	H, G	0	1	1	1	0
GO:0001505	regulation of neurotransmitter levels	R	0	1	1	1	0
GO:0010256	endomembrane system organization	M	0	1	1	1	0
GO:0045005	DNA-templated DNA replication maintenance of fidelity	B, L	0	1	1	1	0
GO:0003954			0	1	1	1	0
GO:0018750	biuret amidohydrolase activity	C	0	1	1	1	0
GO:0046906	tetrapyrrole binding	C	0	1	1	1	0
GO:0051055	negative regulation of lipid biosynthetic process	I	0	1	1	1	0
GO:0001882	nucleoside binding	F	0	1	1	1	0
GO:0008674	2-dehydro-3-deoxy-6-phosphogalactonate aldolase activity	C	0	1	1	1	0
GO:0010565	regulation of cellular ketone metabolic process	H	0	1	1	1	0
GO:0043648	dicarboxylic acid metabolic process	H	0	1	1	1	0
GO:0070925	organelle assembly	R	0	1	1	1	0
GO:0009311	oligosaccharide metabolic process	G	0	1	1	1	0
GO:0102497	scyllo-inositol dehydrogenase (NADP+) activity	C	0	1	1	1	0
GO:0019296	coenzyme M metabolic process	H	0	1	1	1	0
GO:0045428	regulation of nitric oxide biosynthetic process	T, C	0	1	1	1	0

GO_term	GO_term_name	COG_LETTER	*N. niacini*	*N. driksii* strains			
			DSM 2923T	179-C4-2-HS	.79-J 1A1 H:	AT2.8	V4I25
GO:0004064	arylesterase activity	C	0	1	1	1	0
GO:0006198	cAMP catabolic process	F	0	1	1	1	0
GO:0016669	oxidoreductase activity, acting on a sulfur group of donors, cytochrome as acceptor	C	0	1	1	1	0
GO:0047949	glutarate-semialdehyde dehydrogenase (NAD+) activity	R	0	1	1	1	0
GO:0008953	penicillin amidase activity	C	0	1	1	1	0
GO:0006027	glycosaminoglycan catabolic process	G	0	1	1	1	0
GO:0032451	demethylase activity	R	0	1	1	1	0
GO:0018958	phenol-containing compound metabolic process	C	0	1	1	1	0
GO:0000439	transcription factor TFIIH core complex	Y, O	0	1	1	1	0
GO:0018787	4-chlorobenzoyl-CoA dehalogenase activity	C	0	1	1	1	0
GO:0019464			0	1	1	1	0
GO:0005684	U2-type spliceosomal complex	Y, O	0	1	1	1	0
GO:1901987	regulation of cell cycle phase transition	D	0	1	1	1	0
GO:0005768	endosome	M, U	0	1	1	1	0
GO:0048038			0	1	1	1	0
GO:0016684	oxidoreductase activity, acting on peroxide as acceptor	C	0	1	1	1	0
GO:0006779	porphyrin-containing compound biosynthetic process	H	0	1	1	1	0
GO:0006109	regulation of carbohydrate metabolic process	G	0	1	1	1	0
GO:0045912	negative regulation of carbohydrate metabolic process	G	0	1	1	1	0
GO:0016207	4-coumarate-CoA ligase activity	H, C	0	1	1	1	0
GO:0031328	positive regulation of cellular biosynthetic process	C	0	1	1	1	0
GO:0046174	polyol catabolic process	H	0	1	1	1	0
GO:0001101	response to acid chemical	T	0	1	1	1	0
GO:0035194	RNA-mediated post-transcriptional gene silencing	K	0	1	1	1	0
GO:0009262	deoxyribonucleotide metabolic process	F, G	0	1	1	1	0
GO:0006595	polyamine metabolic process	C	0	1	1	1	0
GO:0003729	mRNA binding	J	0	1	1	1	0
GO:0005891	voltage-gated calcium channel complex	M	0	1	1	1	0
GO:0045254	pyruvate dehydrogenase complex	O	0	1	1	1	0
GO:0030135	coated vesicle	U	0	1	1	1	0
GO:0009269	response to desiccation	T	0	1	1	1	0
GO:0004308	exo-alpha-sialidase activity	G	0	1	1	1	0
GO:0040011	locomotion	N	0	1	1	1	0
GO:0043621	protein self-association	O	0	1	1	1	0
GO:0072686	mitotic spindle	Z	0	1	1	1	0
GO:0047693	ATP diphosphatase activity	C	0	1	1	1	0
GO:0106074			0	1	1	1	0
GO:0140303	intramembrane lipid transporter activity	I	0	1	1	1	0
GO:0004252			0	1	1	1	0
GO:0071852	fungal-type cell wall organization or biogenesis	M	0	1	1	1	0
GO:0018818	acetylene hydratase activity	C	0	1	1	1	0
GO:0009891	positive regulation of biosynthetic process	C	0	1	1	1	0
GO:0031347	regulation of defense response	V	0	1	1	1	0
GO:0042273	ribosomal large subunit biogenesis	J	0	1	1	1	0
GO:0017128	phospholipid scramblase activity	I, M	0	1	1	1	0
GO:0022623	proteasome-activating nucleotidase complex	O	0	1	1	1	0
GO:0050776	regulation of immune response	T, W	0	1	1	1	0
GO:0140352	export from cell	U	0	1	1	1	0
GO:0008645	hexose transmembrane transport	G	0	1	1	1	0
GO:0005902	microvillus	Z	0	1	1	1	0
GO:0008111	alpha-methylacyl-CoA racemase activity	H	0	1	1	1	0
GO:0046364	monosaccharide biosynthetic process	H	0	1	1	1	0
GO:0010906	regulation of glucose metabolic process	H, G	0	1	1	1	0
GO:1903561	extracellular vesicle	M, W	0	1	1	1	0
GO:0050778	positive regulation of immune response	T, W	0	1	1	1	0
GO:0015220	choline transmembrane transporter activity	U	0	1	1	1	0
GO:0010876	lipid localization	I	0	1	1	1	0
GO:0005983	starch catabolic process	G	0	1	1	1	0
GO:0071554	cell wall organization or biogenesis	M	0	1	1	1	0
GO:0000027	ribosomal large subunit assembly	J	0	1	1	1	0
GO:0003990	acetylcholinesterase activity	C	0	1	1	1	0
GO:1902652	secondary alcohol metabolic process	H	0	1	1	1	0
GO:1901988	negative regulation of cell cycle phase transition	D	0	1	1	1	0
GO:0009394	2'-deoxyribonucleotide metabolic process	F, G	0	1	1	1	0
GO:0034622			0	1	1	1	0
GO:0042742	defense response to bacterium	V	0	1	1	1	0
GO:0035437	maintenance of protein localization in endoplasmic reticulum	M, U, O	0	1	1	1	0
GO:0000325	plant-type vacuole	U	0	1	1	1	0
GO:0019877			0	1	1	1	0
GO:0022618	ribonucleoprotein complex assembly	J	0	1	1	1	0
GO:0016215	acyl-CoA desaturase activity	Q	0	1	1	1	0
GO:0098734	macromolecule depalmitoylation	C	0	1	1	1	0
GO:0097447	dendritic tree	M	0	1	1	1	0
GO:0098827	endoplasmic reticulum subcompartment	M, U	0	1	1	1	0
GO:0051651	maintenance of location in cell	U	0	1	1	1	0
GO:0006089	lactate metabolic process	C	0	1	1	1	0
GO:0009166	nucleotide catabolic process	F	0	1	1	1	0

GO_term	GO_term_name	COG_LETTER	*N. niacini*	*N. driksii* strains			
			DSM 2923T	179-C4-2-HS	.79-J 1A1 H:	AT2.8	V4I25
GO:0000702	oxidized base lesion DNA N-glycosylase activity	B	0	1	1	1	0
GO:0008994	rhamnulose-1-phosphate aldolase activity	C	0	1	1	1	0
GO:0042726	flavin-containing compound metabolic process	C	0	1	1	1	0
GO:0043799	glycine oxidase activity	E	0	1	1	1	0
GO:0042135	neurotransmitter catabolic process	R	0	1	1	1	0
GO:0080134	regulation of response to stress	T	0	1	1	1	0
GO:0020002	host cell plasma membrane	R	0	1	1	1	0
GO:0009509	chromoplast	M	0	1	1	1	0
GO:0033951	oligosaccharide reducing-end xylanase activity	G	0	1	1	1	0
GO:0003678	DNA helicase activity	B	0	1	1	1	0
GO:0042255	ribosome assembly	J	0	1	1	1	0
GO:0051479	mannosylglycerate biosynthetic process	G	0	1	1	1	0
GO:0006220	pyrimidine nucleotide metabolic process	F	0	1	1	1	0
GO:0004579	dolichyl-diphosphooligosaccharide-protein glycotransferase activity	G	0	1	1	1	0
GO:0019319	hexose biosynthetic process	H	0	1	1	1	0
GO:0006643	membrane lipid metabolic process	I	0	1	1	1	0
GO:0016706	2-oxoglutarate-dependent dioxygenase activity	C	0	1	1	1	0
GO:0006544			0	1	1	1	0
GO:0019692	deoxyribose phosphate metabolic process	G	0	1	1	1	0
GO:0045429	positive regulation of nitric oxide biosynthetic process	C	0	1	1	1	0
GO:0009927	histidine phosphotransfer kinase activity	T	0	1	1	1	0
GO:0005484	SNAP receptor activity	O	0	1	1	1	0
GO:0005770	late endosome	M, U	0	1	1	1	0
GO:0006188			0	1	1	1	0
GO:0004601	peroxidase activity	V	0	1	1	1	0
GO:0052866	phosphatidylinositol phosphate phosphatase activity	C	0	1	1	1	0
GO:0046209	nitric oxide metabolic process	C	0	1	1	1	0
GO:0098533	ATPase dependent transmembrane transport complex	M, O	0	1	1	1	0
GO:0004869	cysteine-type endopeptidase inhibitor activity	T	0	1	1	1	0
GO:2001057	reactive nitrogen species metabolic process	C	0	1	1	1	0
GO:0042770	signal transduction in response to DNA damage	T	0	1	1	1	0
GO:0016842	amidine-lyase activity	C	0	1	1	1	0
GO:0090575	RNA polymerase II transcription regulator complex	Y, O	0	1	1	1	0
GO:0031968	organelle outer membrane	M	1	1	0	0	1
GO:0008940	nitrate reductase activity	C	1	1	0	0	1
GO:0003008	system process	R	1	1	0	0	1
GO:0050463	nitrate reductase [NAD(P)H] activity	C	1	1	0	0	1
GO:0007600	sensory perception	T	1	1	0	0	1
GO:0060918	auxin transport	T	1	1	0	0	1
GO:0009914	hormone transport	T	1	1	0	0	1
GO:0019867	outer membrane	M	1	1	0	0	1
GO:0050877	nervous system process	R	1	1	0	0	1
GO:0005815	microtubule organizing center	Z	1	1	0	0	1
GO:0007606	sensory perception of chemical stimulus	T	1	1	0	0	1
GO:0030990	intraciliary transport particle	O	1	0	1	0	1
GO:0016881	acid-amino acid ligase activity	E	1	0	1	0	1
GO:0030992	intraciliary transport particle B	O	1	0	1	0	1
GO:0048872	homeostasis of number of cells	R	1	0	1	0	1
GO:0042383	sarcolemma	M	1	0	1	0	1
GO:0005244	voltage-gated monoatomic ion channel activity	U	1	0	0	1	1
GO:0008033	tRNA processing	J	1	0	0	1	1
GO:0015932	nucleobase-containing compound transmembrane transporter activity	F, U	1	0	0	1	1
GO:0004530	deoxyribonuclease I activity	B	1	0	0	1	1
GO:0030880	RNA polymerase complex	O	1	0	0	1	1
GO:0005338	nucleotide-sugar transmembrane transporter activity	F, G, U	1	0	0	1	1
GO:0046292	formaldehyde metabolic process	H	1	0	0	1	1
GO:0006054	N-acetylneuraminate metabolic process	G	1	0	0	1	1
GO:0048029	monosaccharide binding	G	1	0	0	1	1
GO:0000428	DNA-directed RNA polymerase complex	O	1	0	0	1	1
GO:0030246	carbohydrate binding	G	1	0	0	1	1
GO:0005669	transcription factor TFIID complex	Y	1	0	0	1	1
GO:0016591	RNA polymerase II, holoenzyme	Y	1	0	0	1	1
GO:0010628	positive regulation of gene expression	K	1	0	0	1	1
GO:0070201	regulation of establishment of protein localization	O	1	0	0	1	1
GO:0055029	nuclear DNA-directed RNA polymerase complex	Y, O	1	0	0	1	1
GO:1903047	mitotic cell cycle process	D	1	0	0	1	1
GO:0019647	formaldehyde assimilation via ribulose monophosphate cycle	H	1	0	0	1	1
GO:0042806	fucose binding	G	1	0	0	1	1
GO:0005775	vacuolar lumen	U	1	0	0	1	1
GO:0016713	oxidoreductase activity, acting on paired donors, with incorporation or reduction of molecular oxygen, reduced iron-sulfur protein as one donor, and incorporation of one atom of oxygen	Q	1	0	0	1	1
GO:0030030	cell projection organization	R	1	0	0	1	1
GO:0019649	formaldehyde assimilation	H	1	0	0	1	1
GO:0001897	cytolysis by symbiont of host cells	V	0	1	1	0	0
GO:0043204	perikaryon	R	0	1	1	0	0
GO:0106261	tRNA uridine(34) acetyltransferase activity	I	0	1	1	0	0

GO_term	GO_term_name	COG_LETTER	*N. niacini*	*N. driksii* strains			
			DSM 2923T	179-C4-2-HS	.79-J 1A1 H	AT2.8	V4I25
GO:0101005	deubiquitinase activity	O	0	1	1	0	0
GO:0009218	pyrimidine ribonucleotide metabolic process	F	0	1	1	0	0
GO:0006222			0	1	1	0	0
GO:0016855	racemase and epimerase activity, acting on amino acids and derivatives	E	0	1	1	0	0
GO:0006526			0	1	1	0	0
GO:0047661	amino-acid racemase activity	E	0	1	1	0	0
GO:0009220	pyrimidine ribonucleotide biosynthetic process	F, H	0	1	1	0	0
GO:0016998	cell wall macromolecule catabolic process	M	0	1	1	0	0
GO:0001907	killing by symbiont of host cells	V	0	1	1	0	0
GO:0019783	ubiquitin-like protein peptidase activity	O	0	1	1	0	0
GO:0006563	L-serine metabolic process	E	0	1	1	0	0
GO:0006760	folic acid-containing compound metabolic process	C	0	1	1	0	0
GO:0005851	eukaryotic translation initiation factor 2B complex	O	0	1	1	0	0
GO:0009173	pyrimidine ribonucleoside monophosphate metabolic process	H	0	1	1	0	0
GO:0008553	P-type proton-exporting transporter activity	P	0	1	1	0	0
GO:0046939	nucleotide phosphorylation	R	0	1	1	0	0
GO:0031570	DNA integrity checkpoint signaling	T	0	1	1	0	0
GO:0043083	synaptic cleft	W	0	1	1	0	0
GO:0009112	nucleobase metabolic process	H	0	1	1	0	0
GO:0006545			0	1	1	0	0
GO:0009070	serine family amino acid biosynthetic process	E	0	1	1	0	0
GO:0019264			0	1	1	0	0
GO:0019694	alkanesulfonate metabolic process	H	0	1	1	0	0
GO:0006059	hexitol metabolic process	G	0	1	1	0	0
GO:0070548	L-glutamine aminotransferase activity	E	0	1	1	0	0
GO:0120013	lipid transfer activity	I, M	0	1	1	0	0
GO:0042558	pteridine-containing compound metabolic process	C	0	1	1	0	0
GO:0019402	galactitol metabolic process	G	0	1	1	0	0
GO:0009174			0	1	1	0	0
GO:0035999			0	1	1	0	0
GO:0008376	acetylgalactosaminyltransferase activity	G	0	1	1	0	0
GO:2001119	methanofuran metabolic process	C	0	1	1	0	0
GO:0019407	hexitol catabolic process	G	0	1	1	0	0
GO:0072528	pyrimidine-containing compound biosynthetic process	H	0	1	1	0	0
GO:0006165	nucleoside diphosphate phosphorylation	R	0	1	1	0	0
GO:0006730			0	1	1	0	0
GO:0006207			0	1	1	0	0
GO:0004372			0	1	1	0	0
GO:0033925	mannosyl-glycoprotein endo-beta-N-acetylglucosaminidase activity	G	0	1	1	0	0
GO:0009130			0	1	1	0	0
GO:0009678	pyrophosphate hydrolysis-driven proton transmembrane transporter activity	P	0	1	1	0	0
GO:0036361	racemase activity, acting on amino acids and derivatives	E	0	1	1	0	0
GO:0030414	peptidase inhibitor activity	T	0	1	1	0	0
GO:0004482	mRNA (guanine-N7-)-methyltransferase activity	H, A	0	1	1	0	0
GO:0061135	endopeptidase regulator activity	T, O	0	1	1	0	0
GO:0035097	histone methyltransferase complex	Y	0	1	1	0	0
GO:2001120	methanofuran biosynthetic process	H	0	1	1	0	0
GO:0044205			0	1	1	0	0
GO:0018537	coenzyme F420-dependent N5,N10-methenyltetrahydromethanopterin reductase activity	C	0	1	1	0	0
GO:0046049	UMP metabolic process	F	0	1	1	0	0
GO:0004866	endopeptidase inhibitor activity	T	0	1	1	0	0
GO:0046112			0	1	1	0	0
GO:0004303	estradiol 17-beta-dehydrogenase activity	Q	0	1	1	0	0
GO:1901269	lipooligosaccharide metabolic process	G	0	1	1	0	0
GO:0006525			0	1	1	0	0
GO:0019405	alditol catabolic process	G	0	1	1	0	0
GO:0016106	sesquiterpenoid biosynthetic process	Q	0	1	1	0	0
GO:0102986	trehalose synthase activity	G	0	1	1	0	0
GO:0000077	DNA damage checkpoint signaling	T	0	1	1	0	0
GO:0045786	negative regulation of cell cycle	T	0	1	1	0	0
GO:0120014	phospholipid transfer activity	I	0	1	1	0	0
GO:0046653			0	1	1	0	0
GO:0010948	negative regulation of cell cycle process	D	0	1	1	0	0
GO:0004222			0	1	1	0	0
GO:0006221	pyrimidine nucleotide biosynthetic process	F, H	0	1	1	0	0
GO:0006206			0	1	1	0	0
GO:0034708	methyltransferase complex	O	0	1	1	0	0
GO:0019856			0	1	1	0	0
GO:0036065	fucosylation	G	0	1	1	0	0
GO:0106035	protein maturation by [4Fe-4S] cluster transfer	K, O	0	1	1	0	0
GO:0009897	external side of plasma membrane	M, W	0	1	0	1	0
GO:0070417	cellular response to cold	T	0	1	0	1	0
GO:0015846	polyamine transport	C	0	1	0	1	0
GO:1900723	negative regulation of protein adenylylation	O	0	1	0	1	0
GO:0050072	m7G(5')pppN diphosphatase activity	R	0	1	0	1	0
GO:0009073			0	1	0	1	0

GO_term	GO_term_name	COG_LETTER	*N. niacini* DSM 2923T	*N. driksii* strains 179-C4-2-HS	.79-J 1A1 H:	AT2.8	V4I25
GO:1900722	regulation of protein adenylylation	O	0	1	0	1	0
GO:0006480	N-terminal protein amino acid methylation	O	0	1	0	1	0
GO:0031970	organelle envelope lumen	M	0	1	0	1	0
GO:0001676	long-chain fatty acid metabolic process	I	0	1	0	1	0
GO:0018117	protein adenylylation	O	0	1	0	1	0
GO:0005771	multivesicular body	M, U	0	1	0	1	0
GO:0035516	oxidative DNA demethylase activity	C	0	1	0	1	0
GO:0012511	monolayer-surrounded lipid storage body	I	0	1	0	1	0
GO:0005727	extrachromosomal circular DNA	B	0	1	0	1	0
GO:0009309	amine biosynthetic process	C	0	1	0	1	0
GO:0016413	O-acetyltransferase activity	I	0	1	0	1	0
GO:0008374	O-acyltransferase activity	I	0	1	0	1	0
GO:0016696	oxidoreductase activity, acting on hydrogen as donor, NAD or NADP as acceptor	C	0	1	0	1	0
GO:0000162			0	1	0	1	0
GO:0009051	pentose-phosphate shunt, oxidative branch	G, C	0	1	0	1	0
GO:0098552	side of membrane	M	0	1	0	1	0
GO:0031514	motile cilium	M, O	0	1	0	1	0
GO:0036038	MKS complex	O	0	1	0	1	0
GO:0044106			0	1	0	1	0
GO:0009164	nucleoside catabolic process	H	0	1	0	1	0
GO:0006586	indolalkylamine metabolic process	C	0	1	0	1	0
GO:0016042	lipid catabolic process	I	0	1	0	1	0
GO:1901658	glycosyl compound catabolic process	G	0	1	0	1	0
GO:0033883	pyridoxal phosphatase activity	C	0	1	0	1	0
GO:0046821	extrachromosomal DNA	B	0	1	0	1	0
GO:0035514	DNA demethylase activity	B	0	1	0	1	0
GO:0042430	indole-containing compound metabolic process	C	0	1	0	1	0
GO:0018666	2,4-dichlorophenol 6-monooxygenase activity	H	0	1	0	1	0
GO:0016972	thiol oxidase activity	C	0	1	0	1	0
GO:0033176	proton-transporting V-type ATPase complex	C	0	1	0	1	0
GO:0009850	auxin metabolic process	C	0	1	0	1	0
GO:0009986	cell surface	M, W	0	1	0	1	0
GO:0007166	cell surface receptor signaling pathway	T	0	1	0	1	0
GO:0015848	spermidine transport	Q	0	1	0	1	0
GO:0018175	protein nucleotidylation	O	0	1	0	1	0
GO:0008922	long-chain fatty acid [acyl-carrier-protein] ligase activity	C	0	1	0	1	0
GO:0006568	tryptophan metabolic process	E	0	1	0	1	0
GO:0003850	2-deoxyglucose-6-phosphatase activity	C	0	1	0	1	0
GO:0051156	glucose 6-phosphate metabolic process	G	0	1	0	1	0
GO:0034308	primary alcohol metabolic process	H	0	1	0	1	0
GO:0016477	cell migration	N	0	1	0	1	0
GO:0042435	indole-containing compound biosynthetic process	H, C	0	1	0	1	0
GO:0050583	hydrogen dehydrogenase (NADP+) activity	C	0	1	0	1	0
GO:0007155	cell adhesion	W	0	1	0	1	0
GO:0009072	aromatic amino acid metabolic process	E	0	1	0	1	0
GO:0046219			0	1	0	1	0
GO:0047614	aconitate delta-isomerase activity	C	0	1	0	1	0
GO:1901568	fatty acid derivative metabolic process	I	0	1	0	1	0
GO:0042401	biogenic amine biosynthetic process	C	0	1	0	1	0
GO:0008747	N-acetylneuraminate lyase activity	C	0	1	0	1	0
GO:0120254	olefinic compound metabolic process	C	0	1	0	1	0
GO:0006098	pentose-phosphate shunt	G, C	0	1	0	1	0
GO:0097729	9+2 motile cilium	O	0	1	0	1	0
GO:0016867	intramolecular transferase activity, transferring acyl groups	R	0	1	0	1	0
GO:0051248	negative regulation of protein metabolic process	O	0	1	0	1	0
GO:0046416	D-amino acid metabolic process	E	0	1	0	1	0
GO:0034656	nucleobase-containing small molecule catabolic process	H	0	1	0	1	0
GO:0034399	nuclear periphery	Y	0	1	0	1	0
GO:0098916	anterograde trans-synaptic signaling	T, W	0	0	1	1	0
GO:0016859	cis-trans isomerase activity	R	0	0	1	1	0
GO:0043173	nucleotide salvage	F	0	0	1	1	0
GO:0033353			0	0	1	1	0
GO:0007283	spermatogenesis	D	0	0	1	1	0
GO:0019365	pyridine nucleotide salvage	F, H	0	0	1	1	0
GO:0047075	2,5-dihydroxypyridine 5,6-dioxygenase activity	C	0	0	1	1	0
GO:0007267	cell-cell signaling	T	0	0	1	1	0
GO:0004301	epoxide hydrolase activity	C	0	0	1	1	0
GO:0016861	intramolecular oxidoreductase activity, interconverting aldoses and ketoses	C	0	0	1	1	0
GO:0047680	aryl-acylamidase activity	C	0	0	1	1	0
GO:0042721	TIM22 mitochondrial import inner membrane insertion complex	M, O	0	0	1	1	0
GO:0001894	tissue homeostasis	R	0	0	1	1	0
GO:0030659	cytoplasmic vesicle membrane	U	0	0	1	1	0
GO:0140457	protein demethylase activity	O	0	0	1	1	0
GO:0006601	creatine biosynthetic process	H, C	0	0	1	1	0
GO:0005518	collagen binding	E	0	0	1	1	0
GO:0009410	response to xenobiotic stimulus	T	0	0	1	1	0

GO_term	GO_term_name	COG_LETTER	*N. niacini*	*N. driksii* strains			
			DSM 2923T	179-C4-2-HS	.79-J 1A1 H	AT2.8	V4I25
GO:0046500	S-adenosylmethionine metabolic process	C	0	0	1	1	0
GO:0043094	cellular metabolic compound salvage	C	0	0	1	1	0
GO:0006805	xenobiotic metabolic process	C	0	0	1	1	0
GO:0099572	postsynaptic specialization	W	0	0	1	1	0
GO:0008866	fructuronate reductase activity	G	0	0	1	1	0
GO:0032452	histone demethylase activity	B, O	0	0	1	1	0
GO:0098984	neuron to neuron synapse	W	0	0	1	1	0
GO:0032279	asymmetric synapse	W	0	0	1	1	0
GO:0046034	ATP metabolic process	H	0	0	1	1	0
GO:0009119	ribonucleoside metabolic process	H	0	0	1	1	0
GO:0006600	creatine metabolic process	C	0	0	1	1	0
GO:0008013	beta-catenin binding	K	0	0	1	1	0
GO:0099536	synaptic signaling	T, W	0	0	1	1	0
GO:0060249	anatomical structure homeostasis	R	0	0	1	1	0
GO:0043102	amino acid salvage	C	0	0	1	1	0
GO:0019284			0	0	1	1	0
GO:0019363	pyridine nucleotide biosynthetic process	F, H	0	0	1	1	0
GO:0006113	fermentation	C	0	0	1	1	0
GO:0071466	cellular response to xenobiotic stimulus	T	0	0	1	1	0
GO:0071265	L-methionine biosynthetic process	E, H	0	0	1	1	0
GO:0071900	regulation of protein serine/threonine kinase activity	O	0	0	1	1	0
GO:0007268	chemical synaptic transmission	T, W	0	0	1	1	0
GO:0019145	aminobutyraldehyde dehydrogenase activity	C	0	0	1	1	0
GO:0045735	nutrient reservoir activity	R	0	0	1	1	0
GO:0198738	cell-cell signaling by wnt	T	0	0	1	1	0
GO:0099537	trans-synaptic signaling	T, W	0	0	1	1	0
GO:0048232	male gamete generation	D	0	0	1	1	0
GO:0019509			0	0	1	1	0
GO:0050076	maleate isomerase activity	C	0	0	1	1	0
GO:0098813	nuclear chromosome segregation	D	0	0	1	1	0
GO:0071267	L-methionine salvage	C	0	0	1	1	0
GO:0047929	gluconate dehydratase activity	C	0	0	1	1	0
GO:0030139	endocytic vesicle	U	0	0	1	1	0
GO:0000148	1,3-beta-D-glucan synthase complex	M	0	0	1	1	0
GO:0005261	monoatomic cation channel activity	P	0	0	1	1	0
GO:0070286	axonemal dynein complex assembly	O	1	0	0	0	1
GO:0099116	tRNA 5'-end processing	J	1	0	0	0	1
GO:0007507	heart development	D	1	0	0	0	1
GO:0043773	coenzyme F420-0 gamma-glutamyl ligase activity	H	1	0	0	0	1
GO:0043295	glutathione binding	C	1	0	0	0	1
GO:0048468	cell development	D	1	0	0	0	1
GO:0050464	nitrate reductase (NADPH) activity	C	1	0	0	0	1
GO:0008482	sulfite oxidase activity	C	1	0	0	0	1
GO:0001578	microtubule bundle formation	Z	1	0	0	0	1
GO:0016485	protein processing	O	1	0	0	0	1
GO:0009040	ureidoglycolate dehydrogenase activity	Q	1	0	0	0	1
GO:0004177			1	0	0	0	1
GO:0051606	detection of stimulus	T	1	0	0	0	1
GO:0035082	axoneme assembly	N	1	0	0	0	1
GO:0005763	mitochondrial small ribosomal subunit	O	1	0	0	0	1
GO:0071422	succinate transmembrane transport	C	1	0	0	0	1
GO:0000966	RNA 5'-end processing	A	1	0	0	0	1
GO:0052618	coenzyme F420-0:L-glutamate ligase activity	H	1	0	0	0	1
GO:2000026	regulation of multicellular organismal development	T, D	1	0	0	0	1
GO:0009527	plastid outer membrane	M	1	0	0	0	1
GO:0008537	proteasome activator complex	O	1	0	0	0	1
GO:0008235	metalloexopeptidase activity	O	1	0	0	0	1
GO:0051336	regulation of hydrolase activity	C	1	0	0	0	1
GO:0015996	chlorophyll catabolic process	H	1	0	0	0	1
GO:0019320	hexose catabolic process	H, G	1	0	0	0	1
GO:0015155	lactose transmembrane transporter activity	G	1	0	0	0	1
GO:0019887	protein kinase regulator activity	O	1	0	0	0	1
GO:1902603	carnitine transmembrane transport	E	1	0	0	0	1
GO:0140677	molecular function activator activity	T	1	0	0	0	1
GO:0010038	response to metal ion	T	1	0	0	0	1
GO:0000314	organellar small ribosomal subunit	J	1	0	0	0	1
GO:0009703	nitrate reductase (NADH) activity	C	1	0	0	0	1
GO:0047110	phenylglyoxylate dehydrogenase (acylating) activity	C	1	0	0	0	1
GO:1904018	positive regulation of vasculature development	D	1	0	0	0	1
GO:0060271	cilium assembly	N	1	0	0	0	1
GO:0042167	heme catabolic process	H	1	0	0	0	1
GO:0140318	protein transporter activity	U	1	0	0	0	1
GO:0120036	plasma membrane bounded cell projection organization	R	1	0	0	0	1
GO:0045766	positive regulation of angiogenesis	D	1	0	0	0	1
GO:0043176	amine binding	R	1	0	0	0	1
GO:0019430			0	1	0	0	0
GO:0072350	tricarboxylic acid metabolic process	H	0	1	0	0	0
GO:0006530	asparagine catabolic process	E	0	1	0	0	0

GO_term	GO_term_name	COG_LETTER	*N. niacini*	*N. driksii* strains			
			DSM 2923T	179-C4-2-HS	.79-J 1A1 H	AT2.8	V4I25
GO:0036064	ciliary basal body	M, Z, O	0	1	0	0	0
GO:0007265	Ras protein signal transduction	T	0	1	0	0	0
GO:0004310	farnesyl-diphosphate farnesyltransferase activity	I	0	1	0	0	0
GO:0097428	protein maturation by iron-sulfur cluster transfer	K, O	0	1	0	0	0
GO:0042931	enterobactin transmembrane transporter activity	U	0	1	0	0	0
GO:0001944	vasculature development	D	0	1	0	0	0
GO:0018112	proline racemase activity	E	0	1	0	0	0
GO:0009065	glutamine family amino acid catabolic process	E	0	1	0	0	0
GO:0071451	cellular response to superoxide	T	0	1	0	0	0
GO:0008886	glyceraldehyde-3-phosphate dehydrogenase (NADP+) (non-phosphorylating) activity	C	0	1	0	0	0
GO:0030286	dynein complex	O	0	1	0	0	0
GO:0006740	NADPH regeneration	C	0	1	0	0	0
GO:0000305	response to oxygen radical	T	0	1	0	0	0
GO:0007264	small GTPase mediated signal transduction	T	0	1	0	0	0
GO:0015175	neutral L-amino acid transmembrane transporter activity	E	0	1	0	0	0
GO:0004675	transmembrane receptor protein serine/threonine kinase activity	O	0	1	0	0	0
GO:0006874	intracellular calcium ion homeostasis	P	0	1	0	0	0
GO:0032934	sterol binding	I	0	1	0	0	0
GO:0006541			0	1	0	0	0
GO:0030003	intracellular monoatomic cation homeostasis	R	0	1	0	0	0
GO:0001525	angiogenesis	D	0	1	0	0	0
GO:0000323	lytic vacuole	U	0	1	0	0	0
GO:0006402	mRNA catabolic process	K, A	0	1	0	0	0
GO:0001568	blood vessel development	D	0	1	0	0	0
GO:0005858	axonemal dynein complex	Z	0	1	0	0	0
GO:0006101	citrate metabolic process	H	0	1	0	0	0
GO:0000810	diacylglycerol diphosphate phosphatase activity	C	0	1	0	0	0
GO:0004341	gluconolactonase activity	C	0	1	0	0	0
GO:0035295	tube development	D	0	1	0	0	0
GO:0004311	farnesyltranstransferase activity	I	0	1	0	0	0
GO:0016712	oxidoreductase activity, acting on paired donors, with incorporation or reduction of molecular oxygen, reduced flavin or flavoprotein as one donor, and incorporation of one atom of oxygen	H	0	1	0	0	0
GO:0005875	microtubule associated complex	O	0	1	0	0	0
GO:0048269	methionine adenosyltransferase complex	O	0	1	0	0	0
GO:0034338	short-chain carboxylesterase activity	C	0	1	0	0	0
GO:0006490	oligosaccharide-lipid intermediate biosynthetic process	I, G	0	1	0	0	0
GO:0015370	solute:sodium symporter activity	P	0	1	0	0	0
GO:0015923	mannosidase activity	G	0	1	0	0	0
GO:0071450	cellular response to oxygen radical	T	0	1	0	0	0
GO:0070085	glycosylation	G	0	1	0	0	0
GO:0071986	Ragulator complex	M, U, O	0	1	0	0	0
GO:0018206	peptidyl-methionine modification	O	0	1	0	0	0
GO:0000303	response to superoxide	T	0	1	0	0	0
GO:0006457	protein folding	K, O	0	1	0	0	0
GO:0018662	phenol 2-monooxygenase activity	H	0	1	0	0	0
GO:0070330	aromatase activity	H	0	1	0	0	0
GO:0005343	organic acid:sodium symporter activity	U, C	0	1	0	0	0
GO:0035239	tube morphogenesis	D	0	1	0	0	0
GO:0098609	cell-cell adhesion	W	0	1	0	0	0
GO:0051052	regulation of DNA metabolic process	B	0	1	0	0	0
GO:0006528	asparagine metabolic process	E	0	1	0	0	0
GO:0019199	transmembrane receptor protein kinase activity	O	0	1	0	0	0
GO:0004567	beta-mannosidase activity	G	0	1	0	0	0
GO:0048514	blood vessel morphogenesis	D	0	1	0	0	0
GO:0051996	squalene synthase activity	I	0	1	0	0	0
GO:0017153	sodium:dicarboxylate symporter activity	U, C	0	1	0	0	0
GO:0048585	negative regulation of response to stimulus	T	0	1	0	0	0
GO:0002084	protein depalmitoylation	O	0	0	1	0	0
GO:0098599	palmitoyl hydrolase activity	I	0	0	1	0	0
GO:0030545	signaling receptor regulator activity	T	0	0	1	0	0
GO:0042773	ATP synthesis coupled electron transport	C	0	0	1	0	0
GO:0010346	shoot axis formation	D	0	0	1	0	0
GO:0071013	catalytic step 2 spliceosome	O	0	0	1	0	0
GO:0022008	neurogenesis	D	0	0	1	0	0
GO:0061617	MICOS complex	M, O	0	0	1	0	0
GO:0016886	ligase activity, forming phosphoric ester bonds	R	0	0	1	0	0
GO:0018459	carveol dehydrogenase activity	Q	0	0	1	0	0
GO:0010223	secondary shoot formation	D	0	0	1	0	0
GO:0034702	ion channel complex	M, O	0	0	1	0	0
GO:0005789	endoplasmic reticulum membrane	M	0	0	1	0	0
GO:0043408	regulation of MAPK cascade	T	0	0	1	0	0
GO:0009060	aerobic respiration	C	0	0	1	0	0
GO:0042157	lipoprotein metabolic process	O	0	0	1	0	0
GO:0014069	postsynaptic density	W	0	0	1	0	0
GO:0018457	perillyl-alcohol dehydrogenase activity	Q	0	0	1	0	0
GO:0035601	protein deacylation	O	0	0	1	0	0

GO_term	GO_term_name	COG_LETTER	*N. niacini*	*N. driksii* strains			
			DSM 2923T	179-C4-2-HS	.79-J 1A1 H:	AT2.8	V4I25
GO:0015453	oxidoreduction-driven active transmembrane transporter activity	C	0	0	1	0	0
GO:1901600	strigolactone metabolic process	C	0	0	1	0	0
GO:0048018	receptor ligand activity	T	0	0	1	0	0
GO:0008410	CoA-transferase activity	H	0	0	1	0	0
GO:0019948	SUMO activating enzyme activity	O	0	0	1	0	0
GO:0050661			0	0	1	0	0
GO:0047739	cephalosporin-C deacetylase activity	C	0	0	1	0	0
GO:0030133	transport vesicle	M, U	0	0	1	0	0
GO:0030546	signaling receptor activator activity	T	0	0	1	0	0
GO:0010647	positive regulation of cell communication	T	0	0	1	0	0
GO:0022900	electron transport chain	C	0	0	1	0	0
GO:0003972	RNA ligase (ATP) activity	A	0	0	1	0	0
GO:0012501	programmed cell death	D	0	0	1	0	0
GO:0006119	oxidative phosphorylation	C	0	0	1	0	0
GO:0009967	positive regulation of signal transduction	T	0	0	1	0	0
GO:0006672	ceramide metabolic process	C	0	0	1	0	0
GO:0030163	protein catabolic process	O	0	0	1	0	0
GO:0042176	regulation of protein catabolic process	O	0	0	1	0	0
GO:0017119	Golgi transport complex	O	0	0	1	0	0
GO:1905347	endodeoxyribonuclease complex	O	0	0	1	0	0
GO:0016708	oxidoreductase activity, acting on paired donors, with incorporation or reduction of molecular oxygen, NAD(P)H as one donor, and incorporation of two atoms of oxygen into one donor	I	0	0	1	0	0
GO:1901601	strigolactone biosynthetic process	H	0	0	1	0	0
GO:0007399	nervous system development	D	0	0	1	0	0
GO:0018200	peptidyl-glutamic acid modification	O	0	0	1	0	0
GO:0042775	mitochondrial ATP synthesis coupled electron transport	C	0	0	1	0	0
GO:0019500	cyanide catabolic process	C	0	0	1	0	0
GO:0019787	ubiquitin-like protein transferase activity	U	0	0	1	0	0
GO:0050388	uronate dehydrogenase activity	C	0	0	1	0	0
GO:0042159	lipoprotein catabolic process	O	0	0	1	0	0
GO:0006090	pyruvate metabolic process	H	0	0	1	0	0
GO:0022842	narrow pore channel activity	U	0	0	1	0	0
GO:0023056	positive regulation of signaling	T	0	0	1	0	0
GO:0050878	regulation of body fluid levels	T	0	0	1	0	0
GO:1901652	response to peptide	T	0	0	1	0	0
GO:0018710	acetone carboxylase activity	C	0	0	1	0	0
GO:0006687	glycosphingolipid metabolic process	I, G	0	0	1	0	0
GO:0022904	respiratory electron transport chain	C	0	0	1	0	0
GO:0008452	RNA ligase activity	A	0	0	1	0	0
GO:0006914	autophagy	O	0	0	1	0	0
GO:0010243	response to organonitrogen compound	T	0	0	1	0	0
GO:0016841	ammonia-lyase activity	C	0	0	1	0	0
GO:0098793	presynapse	W	0	0	1	0	0
GO:0008219	cell death	D	0	0	1	0	0
GO:0061919	process utilizing autophagic mechanism	O	0	0	1	0	0
GO:0097060	synaptic membrane	M, W	0	0	1	0	0
GO:0043434	response to peptide hormone	T	0	0	1	0	0
GO:0019205	nucleobase-containing compound kinase activity	P, T	0	0	1	0	0
GO:0050664	oxidoreductase activity, acting on NAD(P)H, oxygen as acceptor	C	0	0	1	0	0
GO:0048476	Holliday junction resolvase complex	O	0	0	1	0	0
GO:0045177	apical part of cell	M	0	0	1	0	0
GO:0046514	ceramide catabolic process	C	0	0	1	0	0
GO:0051861	glycolipid binding	I, G	0	0	1	0	0
GO:0009611	response to wounding	T	0	0	1	0	0
GO:0071014	post-mRNA release spliceosomal complex	Y, O	0	0	1	0	0
GO:0008417	fucosyltransferase activity	G	0	0	1	0	0
GO:0008474	palmitoyl-(protein) hydrolase activity	H, I, O	0	0	1	0	0
GO:0034703	cation channel complex	M, O	0	0	1	0	0
GO:0019499	cyanide metabolic process	C	0	0	1	0	0
GO:0046479	glycosphingolipid catabolic process	G	0	0	1	0	0
GO:0043785	cinnamoyl-CoA:phenyllactate CoA-transferase activity	H	0	0	1	0	0
GO:0019068	virion assembly	X	0	0	1	0	0
GO:0042734	presynaptic membrane	M, W	0	0	1	0	0
GO:0008641	ubiquitin-like modifier activating enzyme activity	O	0	0	1	0	0
GO:0071498	cellular response to fluid shear stress	T	0	0	1	0	0
GO:0048699	generation of neurons	D	0	0	1	0	0
GO:0019646	aerobic electron transport chain	C	0	0	1	0	0
GO:1903509	liposaccharide metabolic process	I, G	0	0	1	0	0
GO:0045333	cellular respiration	C	0	0	1	0	0
GO:0008360	regulation of cell shape	M	0	0	0	1	0
GO:0009206	purine ribonucleoside triphosphate biosynthetic process	H, C	0	0	0	1	0
GO:0019684	photosynthesis, light reaction	C	0	0	0	1	0
GO:0035304	regulation of protein dephosphorylation	O	0	0	0	1	0
GO:0006023	aminoglycan biosynthetic process	G	0	0	0	1	0
GO:0009141	nucleoside triphosphate metabolic process	H	0	0	0	1	0
GO:0047828	D-lyxose ketol-isomerase activity	G	0	0	0	1	0
GO:0009018	sucrose phosphorylase activity	G	0	0	0	1	0

GO_term	GO_term_name	COG_LETTER	*N. niacini*	*N. driksii* strains			
			DSM 2923T	179-C4-2-HS	.79-J 1A1 H:	AT2.8	V4I25
GO:0008535	respiratory chain complex IV assembly	C	0	0	0	1	0
GO:1905114	cell surface receptor signaling pathway involved in cell-cell signaling	R	0	0	0	1	0
GO:0019534	toxin transmembrane transporter activity	V, U	0	0	0	1	0
GO:0044038	cell wall macromolecule biosynthetic process	M	0	0	0	1	0
GO:0051051	negative regulation of transport	T	0	0	0	1	0
GO:0000278	mitotic cell cycle	Y	0	0	0	1	0
GO:0015986			0	0	0	1	0
GO:0042759	long-chain fatty acid biosynthetic process	I	0	0	0	1	0
GO:0015791	polyol transmembrane transport	Q	0	0	0	1	0
GO:0006040	amino sugar metabolic process	G	0	0	0	1	0
GO:0072599	establishment of protein localization to endoplasmic reticulum	O	0	0	0	1	0
GO:0061980	regulatory RNA binding	A	0	0	0	1	0
GO:0016108	tetraterpenoid metabolic process	I	0	0	0	1	0
GO:0000902	cell morphogenesis	D	0	0	0	1	0
GO:0017004	cytochrome complex assembly	C	0	0	0	1	0
GO:0009176	pyrimidine deoxyribonucleoside monophosphate metabolic process	H	0	0	0	1	0
GO:0043025	neuronal cell body	R	0	0	0	1	0
GO:0016116	carotenoid metabolic process	I	0	0	0	1	0
GO:0033617	mitochondrial cytochrome c oxidase assembly	C	0	0	0	1	0
GO:0045273	respiratory chain complex II	O	0	0	0	1	0
GO:0022603	regulation of anatomical structure morphogenesis	T, D	0	0	0	1	0
GO:0010008	endosome membrane	M, U	0	0	0	1	0
GO:0034458	3'-5' RNA helicase activity	A	0	0	0	1	0
GO:0050269	coniferyl-aldehyde dehydrogenase activity	C	0	0	0	1	0
GO:0006024	glycosaminoglycan biosynthetic process	G	0	0	0	1	0
GO:0009142	nucleoside triphosphate biosynthetic process	H, C	0	0	0	1	0
GO:0019665	anaerobic amino acid catabolic process	C	0	0	0	1	0
GO:0044242	cellular lipid catabolic process	I	0	0	0	1	0
GO:0009273	peptidoglycan-based cell wall biogenesis	M	0	0	0	1	0
GO:0003975	UDP-N-acetylglucosamine-dolichyl-phosphate N-acetylglucosaminephosphotransferase activity	P, T	0	0	0	1	0
GO:0070178	D-serine metabolic process	E	0	0	0	1	0
GO:0016311	dephosphorylation	C	0	0	0	1	0
GO:0022622	root system development	D	0	0	0	1	0
GO:0051864	histone H3K36 demethylase activity	C	0	0	0	1	0
GO:0045047	protein targeting to ER	O	0	0	0	1	0
GO:0016101	diterpenoid metabolic process	I	0	0	0	1	0
GO:0051668	localization within membrane	U	0	0	0	1	0
GO:0015140	malate transmembrane transporter activity	C	0	0	0	1	0
GO:0009199	ribonucleoside triphosphate metabolic process	H	0	0	0	1	0
GO:0042602	riboflavin reductase (NADPH) activity	C	0	0	0	1	0
GO:0008753	NADPH dehydrogenase (quinone) activity	C	0	0	0	1	0
GO:0006869	lipid transport	I	0	0	0	1	0
GO:0004517	nitric-oxide synthase activity	H	0	0	0	1	0
GO:0098794	postsynapse	W	0	0	0	1	0
GO:0031594	neuromuscular junction	W	0	0	0	1	0
GO:0019666	nitrogenous compound fermentation	C	0	0	0	1	0
GO:0004645	1,4-alpha-oligoglucan phosphorylase activity	G	0	0	0	1	0
GO:0006470	protein dephosphorylation	O	0	0	0	1	0
GO:0007610	behavior	R	0	0	0	1	0
GO:0009145	purine nucleoside triphosphate biosynthetic process	H, C	0	0	0	1	0
GO:0009205	purine ribonucleoside triphosphate metabolic process	H	0	0	0	1	0
GO:0090481	pyrimidine nucleotide-sugar transmembrane transport	F	0	0	0	1	0
GO:1901359	tungstate binding	P	0	0	0	1	0
GO:0022604	regulation of cell morphogenesis	T, D	0	0	0	1	0
GO:0016055	Wnt signaling pathway	T	0	0	0	1	0
GO:0006754	ATP biosynthetic process	H	0	0	0	1	0
GO:0033204	ribonuclease P RNA binding	J	0	0	0	1	0
GO:0052729	dimethylglycine N-methyltransferase activity	H	0	0	0	1	0
GO:0016160	amylase activity	G	0	0	0	1	0
GO:0044309	neuron spine	M	0	0	0	1	0
GO:0009201	ribonucleoside triphosphate biosynthetic process	H, C	0	0	0	1	0
GO:0007033	vacuole organization	U	0	0	0	1	0
GO:1902600	proton transmembrane transport	P	0	0	0	1	0
GO:0036348	hydantoin racemase activity	Q	0	0	0	1	0
GO:0004409	homoaconitate hydratase activity	C	0	0	0	1	0
GO:0033588	elongator holoenzyme complex	O	0	0	0	1	0
GO:0005881	cytoplasmic microtubule	Z	0	0	0	1	0
GO:0009219	pyrimidine deoxyribonucleotide metabolic process	F	0	0	0	1	0
GO:0042178	xenobiotic catabolic process	C	0	0	0	1	0
GO:0042162	telomeric DNA binding	B	0	0	0	1	0
GO:0035303	regulation of dephosphorylation	T, C	0	0	0	1	0
GO:0015979	photosynthesis	C	0	0	0	1	0
GO:0005874	microtubule	Z	0	0	0	1	0
GO:0055082	intracellular chemical homeostasis	R	0	0	0	1	0
GO:0009144	purine nucleoside triphosphate metabolic process	H	0	0	0	1	0

GO_term	GO_term_name	COG_LETTER	*N. niacini*	*N. driksii* strains			
			DSM 2923T	179-C4-2-HS	.79-J 1A1 H:	AT2.8	V4I25
GO:0050498	oxidoreductase activity, acting on paired donors, with incorporation or reduction of molecular oxygen, with 2-oxoglutarate as one donor, and the other dehydrogenated	C	0	0	0	1	0
GO:0051223	regulation of protein transport	O	0	0	0	1	0
GO:0004336	galactosylceramidase activity	G	0	0	0	1	0
GO:0000270			0	0	0	1	0
GO:0060070	canonical Wnt signaling pathway	T	0	0	0	1	0
GO:0015985			0	0	0	1	0
GO:0006658	phosphatidylserine metabolic process	C	0	0	0	1	0
GO:0070589	cellular component macromolecule biosynthetic process	R	0	0	0	1	0
GO:0008186	ATP-dependent activity, acting on RNA	C	0	0	0	1	0
GO:0050133	N6-hydroxylysine O-acetyltransferase activity	I	0	0	0	1	0
GO:0048364	root development	D	0	0	0	1	0
GO:0010154	fruit development	D	0	0	0	1	0
GO:0072657	protein localization to membrane	O	0	0	0	1	0
GO:0009252			0	0	0	1	0
GO:0072669	tRNA-splicing ligase complex	O	0	0	0	1	0
GO:0043197	dendritic spine	W	0	0	0	1	0
GO:0006584	catecholamine metabolic process	C	0	0	0	1	0
GO:0019662	non-glycolytic fermentation	C	0	0	0	1	0
GO:0097718	disordered domain specific binding	O	0	0	0	1	0
